# Author Correction: Extracellular vesicle-based targeted protein degradation platform for multiple extracellular proteins

**DOI:** 10.1038/s44321-026-00439-z

**Published:** 2026-05-19

**Authors:** Bide Tong, Xiaoguang Zhang, Dingchao Zhu, Yulei Wang, Junyu Wei, Zixuan Ou, Huaizhen Liang, Hanpeng Xu, Zhengdong Zhang, Jie Lei, Xingyu Zhou, Di Wu, Yu Song, Kun Wang, Xiaobo Feng, Lei Tan, Zhiwei Liao, Cao Yang

**Affiliations:** 1 Department of Orthopaedics, Union Hospital, Tongji Medical College, Huazhong University of Science and Technology, Wuhan, China; 2 Shenzhen Huazhong University of Science and Technology Research institute, Shenzhen, China; 3 Department of Histology and Embryology, Tongji Medical College, Huazhong University of Science and Technology, Wuhan, China

## Abstract

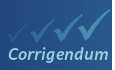

**Correction to:** *EMBO Molecular Medicine* (2026) 18:759–794. 10.1038/s44321-025-00371-8 | Published online 12 January 2026

The authors contacted the journal after discovering that the author contribution statement was missing and that an error was present in the β-actin blot in Figure EV1P. The authors provided the raw data for the affected figure, which the journal has reviewed. The journal agrees to implement the following corrections.

**The co-first author statement is corrected**.

**Figure EV1P is withdrawn and replaced.**

**Figure EV1P:**
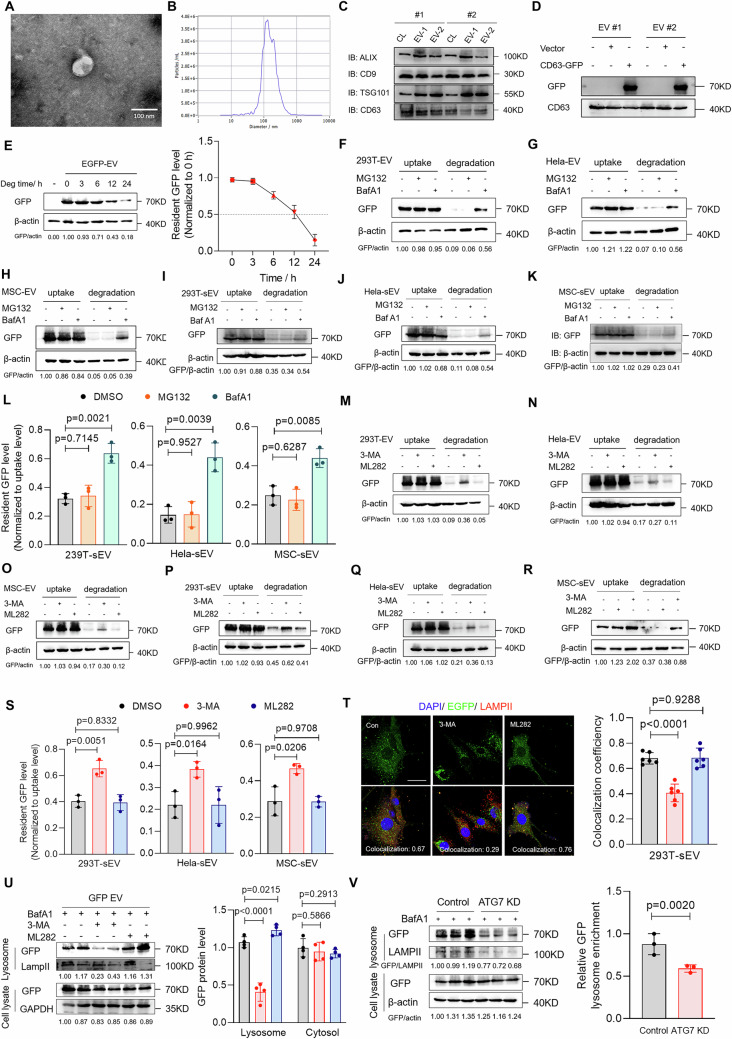
Incorrect published figure.

**Corresponding raw data is published with this author correction**.

The co-first author contribution statement has been corrected to read: “**B.T., X.Z., D.Z., and Y.W. contributed equally to this work**.”

**Author statement:**

1. Author Contributions

The statement indicating that the first four authors contributed equally as co-first authors was deleted due to formatting issues during the final publication process. It was present in the author contribution section in the earlier versions of the submission.

2. Figure EV1P

After careful rechecking of our experimental data, we found that the β-actin blot image in Figure EV1p was an incorrect image. We have now replaced it with the correct image and verified that no other panels or data are affected.

**Figure EV2:**
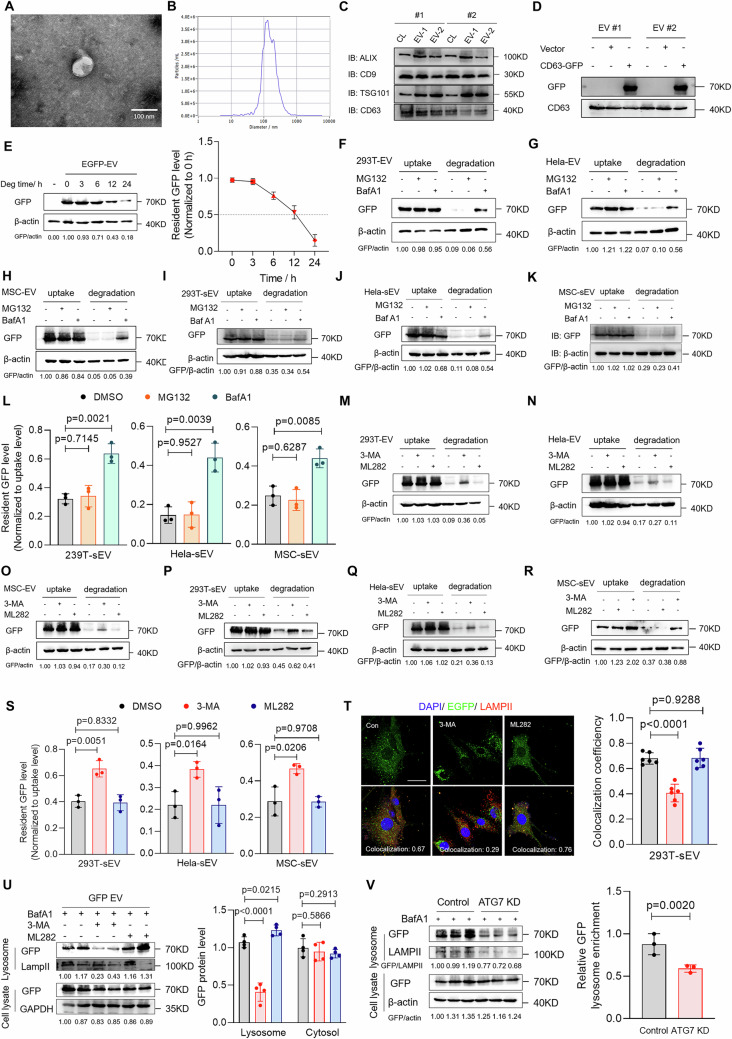
Corrected.

This change does not alter any results, interpretations, or conclusions of the study.

## Supplementary information

Source Data

