# Supplementary material for: Extracellular vesicle-based targeted protein degradation platform for multiple extracellular proteins
Source: EMBO Mol Med. 2026 Jan 12;18(2):759–94. doi: 10.1038/s44321-025-00371-8 (PMC12905291; doi:10.1038/s44321-025-00371-8)
Supplement: Supplementary file 1 — Appendix [file 44321_2025_371_MOESM1_ESM.pdf]

Appendix

EV-based targeted protein degradation platform for multiple extracellular targeted proteins degradation

Bide Tong[1,2#], Xiaoguang Zhang[1#], Dingchao Zhu[1#], Yulei Wang[3#], Junyu Wei[1], Zixuan Ou[1], Huaizhen Liang[1], Hanpeng Xu[1], Zhengdong Zhang[1], Jie Lei[1], Xingyu Zhou[1], Di Wu[1], Yu Song[1], Kun Wang[1], Xiaobo Feng[1], Lei Tan[1*], Zhiwei Liao[1,2*], Cao Yang[1,2*]

[1] Department of Orthopaedics, Union Hospital, Tongji Medical College, Huazhong University of Science and Technology, Wuhan, China.

[2] Shenzhen Huazhong University of Science and Technology Research institute, Shenzhen, China.

[3] Department of Histology and Embryology, Tongji Medical College, Huazhong University of Science and Technology, Wuhan, China.

Context	Page
Appendix Figure S1-9	2-14

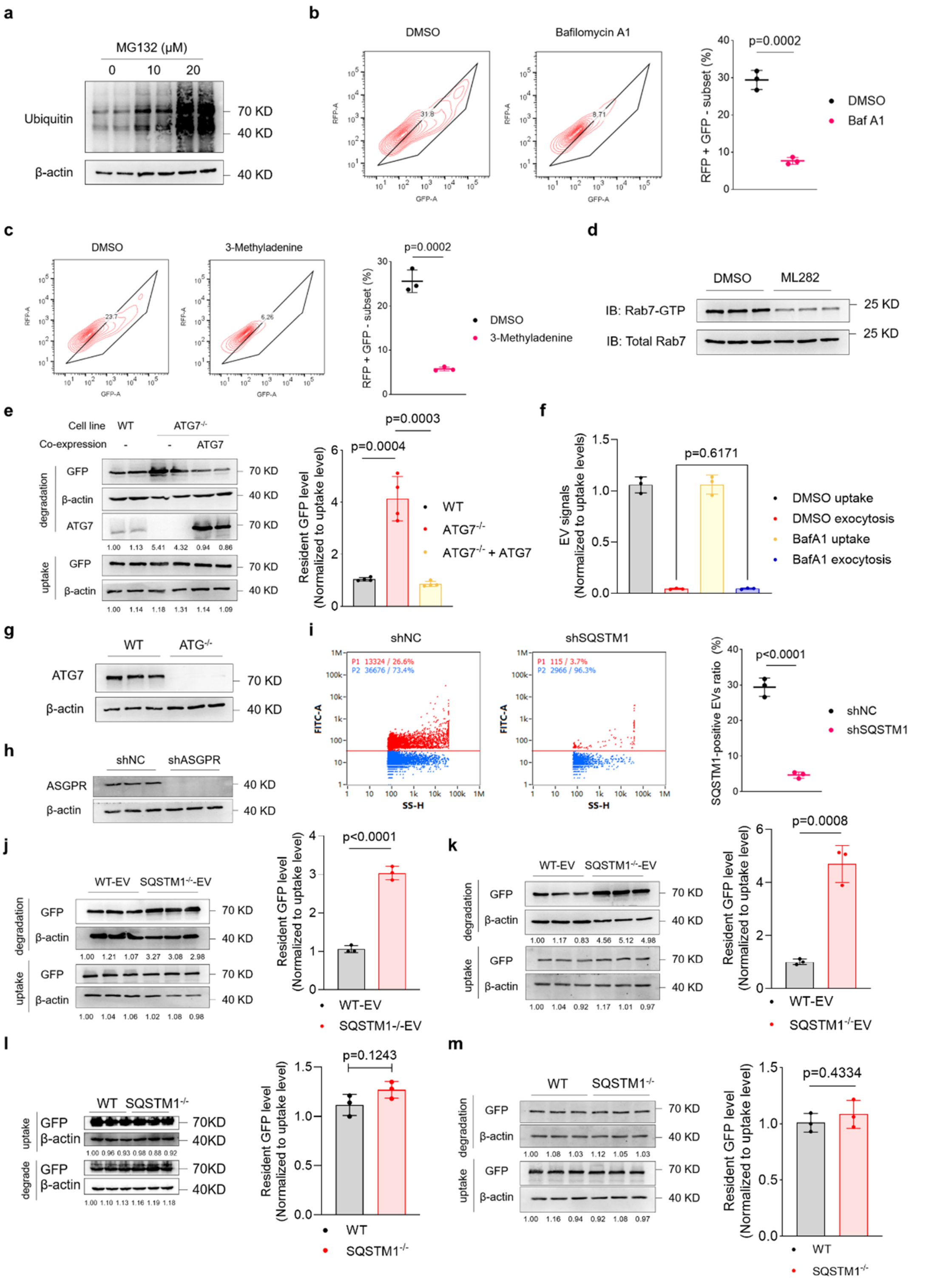

Appendix Figure S1

a HeLa cells were treated with 0, 10, and 20 μM MG132 for 12 hours, and the levels of protein ubiquitination were assessed using Western blot analysis (*n*=2); **b** Hela cells expressing GFP-RFP-LC3 were treated with DMSO or 50 nM BafA1 for 12 hours, and the intracellular GFP and RFP signals were detected using flow cytometry (*n*=3); **c** Hela cells expressing GFP-RFP-LC3 were treated with DMSO or 5 mM 3-Methyladenine for 12 hours, and the intracellular GFP and RFP signals were detected using flow cytometry (*n*=3); **d** HeLa cells were treated with 20 μM ML282

for 12 hours. The levels of total Rab7 and active Rab7 (Rab7-GTP) were evaluated using Western blot with Rab7 antibody and Rab7-GTP-specific antibody, respectively (*n*=3); **e** Representative immunoblot images and quantitative analysis of the resident GFP level of HEK293T-derived EVs in the wild type, ATG7$^{-/-}$ and ATG7 rescue Hela cell lines (*n*=3); **f** The exocytosis levels of CFSE-labeled EVs were measured in both the DMSO and BafA1-pretreated group, with the uptake level at 12 hours used to standardize the CFSE-EV signal within each group (*n*=3). **g** The expression levels of ATG7 protein in wild-type and ATG7 KO cell lines was detected by western blotting (*n*=3); **h** The ASGPR levels in HepG2 cells after shASGPR treatment were detected by western blotting, and shScramble was used as a negative control (*n*=3); **i** The proportion of SQSTM1-positive EVs in the shNC and shSQSTM1 groups were quantitatively detected using NanoFCM (*n*=3); **j-k** Representative immunoblot images and quantitative analysis of the resident GFP levels of wild type and *SQSTM1*$^{-/-}$ EVs in the HEK293T (j) and nucleus pulposus cells (k) (*n*=3); **l-m** Representative immunoblot images and quantitative analysis of the resident GFP level of HEK293T (l) and Hela (m)-derived GFP-EVs in the wild type and *SQSTM1*$^{-/-}$ Hela cell lines (*n*=3). Date were analysed by unpaired two-tailed t-tests (**b, c, e, f, i, j, k, l, m**). Data were shown as mean ± SD. Each *n* in **b, c, e, f, i, j, k, l, m** is biological independent samples.

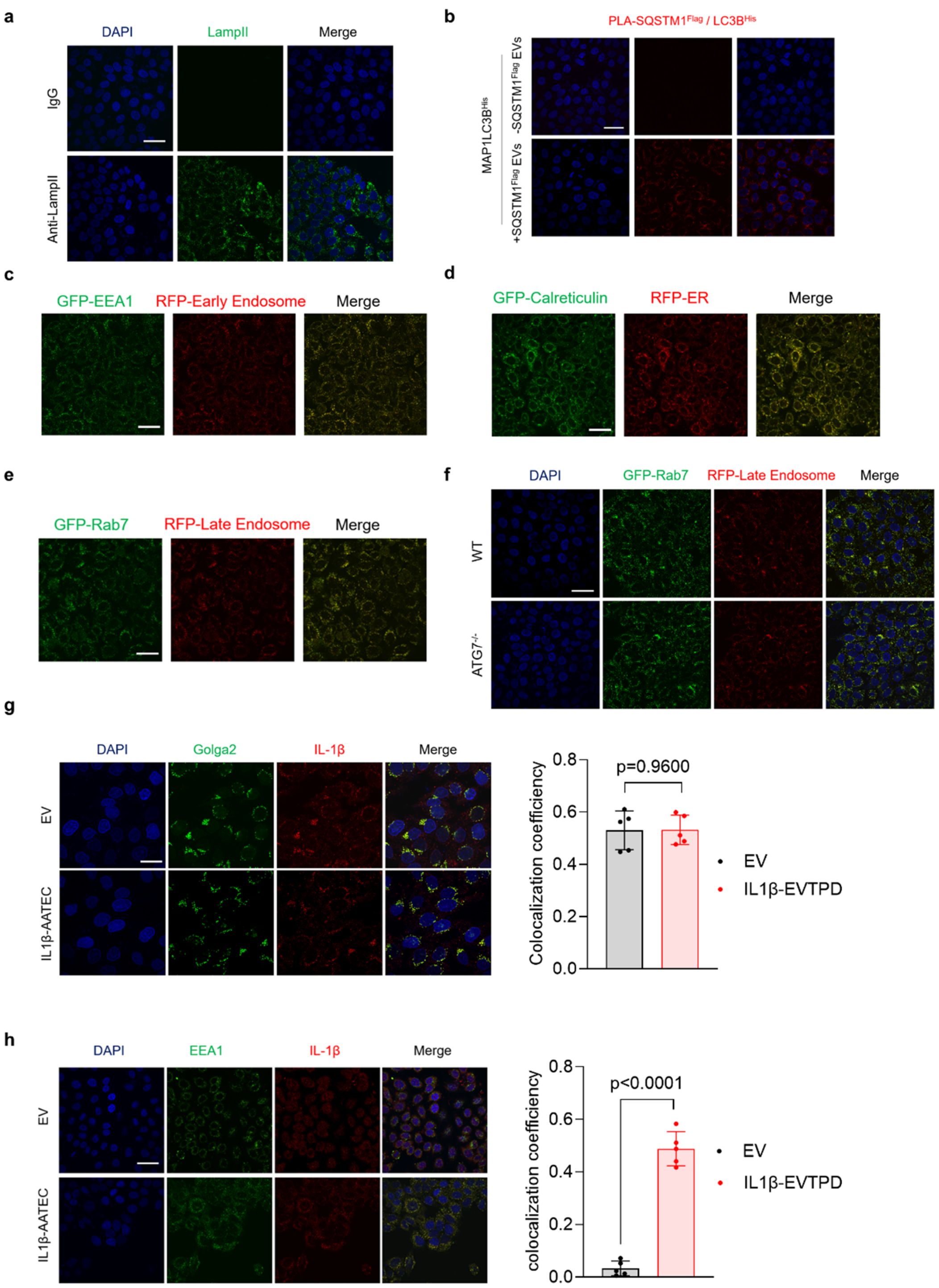

Appendix Figure S2

a Representative fluorescence images show the localization of LampII in Hela cells. The negative control group used IgG antibody as the primary antibody (Scale bar: 10 µm); **b** PLA assay were used to detect the interaction sites (Red) of EV-Flag-SQSTM1 and intracellular His-MAP1LC3B in Hela cells. The group without SQSTM1Flag-EVs addition was set as the negative control group (Scale bar: 10µm); **c** Representative fluorescence image of Hela cells co-expressing GFP-EEA1 and RFP-Early Endosome (Scale bar: 10µm); **d** Representative fluorescence image of Hela cells co-expressing GFP-Calreticulin and RFP-ER (Scale bar: 10µm); **e** Representative fluorescence image of Hela cells co-expressing GFP-Rab7 and RFP-Late Endosome (Scale bar: 10µm); **f** Representative

fluorescence images show the localization of GFP-Rab7, RFP-Late Endosome in wild type and *ATG7*$^{-/-}$ Hela cells (Scale bar: 10 μm); **g** Fluorescence colocalization analysis of IL1b and Golga2 in Hela cells after EV or IL1b-EVTPD treatment for 24 h (Scale bar: 10 μm) (*n*=5); **h** Fluorescence colocalization analysis of IL1b and EEA1 in Hela cells after EV or IL1b-EVTPD treatment for 24 h (Scale bar: 10 μm) (*n*=5). Date were analysed by unpaired two-tailed t-tests (**g, h**). Data were shown as mean ± SD. Each *n* in **g, h** is biological independent samples.

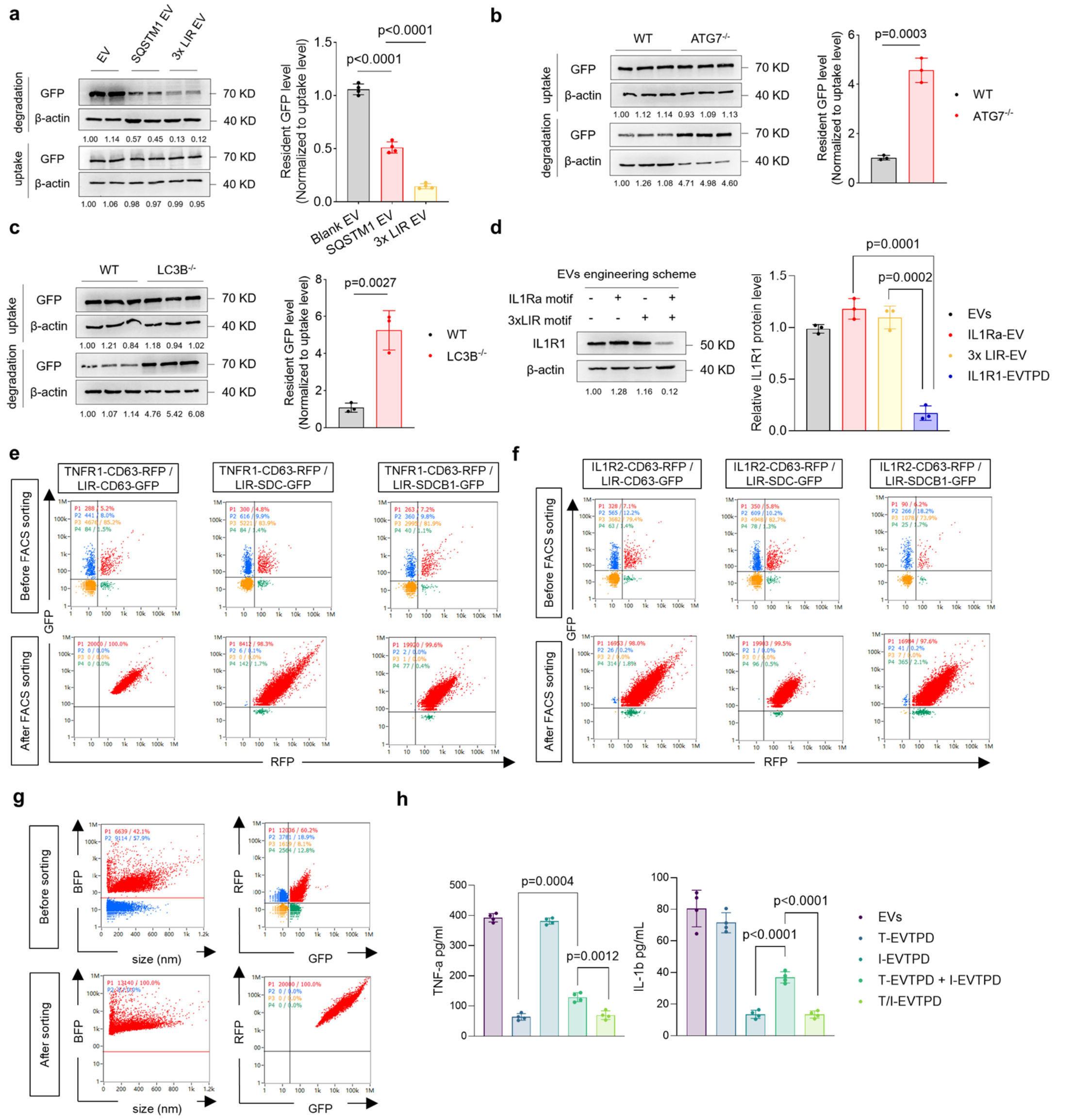

Appendix Figure S3

a Representative immunoblot images and quantitative analysis of the resident GFP level of blank, SQSTM1 overexpression and 3x LIR motifs engineering EVs in Hela cells (*n*=3); **b-c** Representative immunoblot images and quantitative analysis of the resident GFP level of 3x LIR motifs-loaded EVs in the wild type, *ATG7*$^{-/-}$ (a) and *MAP1LC3B1*$^{-/-}$ (b) Hela cell lines (*n*=3)**; d** Protein level analysis of IL1R1 levels in Hela cells pretreated with blank EVs, IL1Ra-loaded EVs, 3xLIR-loaded EVs and IL1R1-EVTPD (loaded with both IL1Ra and 3xLIR motifs) for 24 h (n=3)**; e** The loading efficiency of degradation signals (GFP) and targeting signals (RFP) on TNFa-EVTPD before and after fluorescence-activated sorting were detected using NanoFCM; **e** The loading efficiency of degradation signals (GFP) and targeting signals (RFP) on IL1b-EVTPD before and after fluorescence-activated sorting were detected using NanoFCM; **g** The loading efficiency of degradation signals (BFP) and targeting signals (RFP and GFP) on engineering EVs before and after fluorescence-activated sorting were detected using NanoFCM; **h** ELISAs were used to detect the degradation of TNF-a and IL-1b by blank EVs, TNFa-EVTPD, IL1b-EVTPD, mix pool of TNFa-EVTPD and IL1b-EVTPD, T/I-EVTPD at the concentration of 10 × 10^3 per cell after coincubation

with NPCs for 24 h (*n*=4). Date were analysed by unpaired two-tailed t-tests (**a, b, c, d, h**). Data were shown as mean ± SD. Each *n* in **a, b, c, d, h** is biological independent samples.

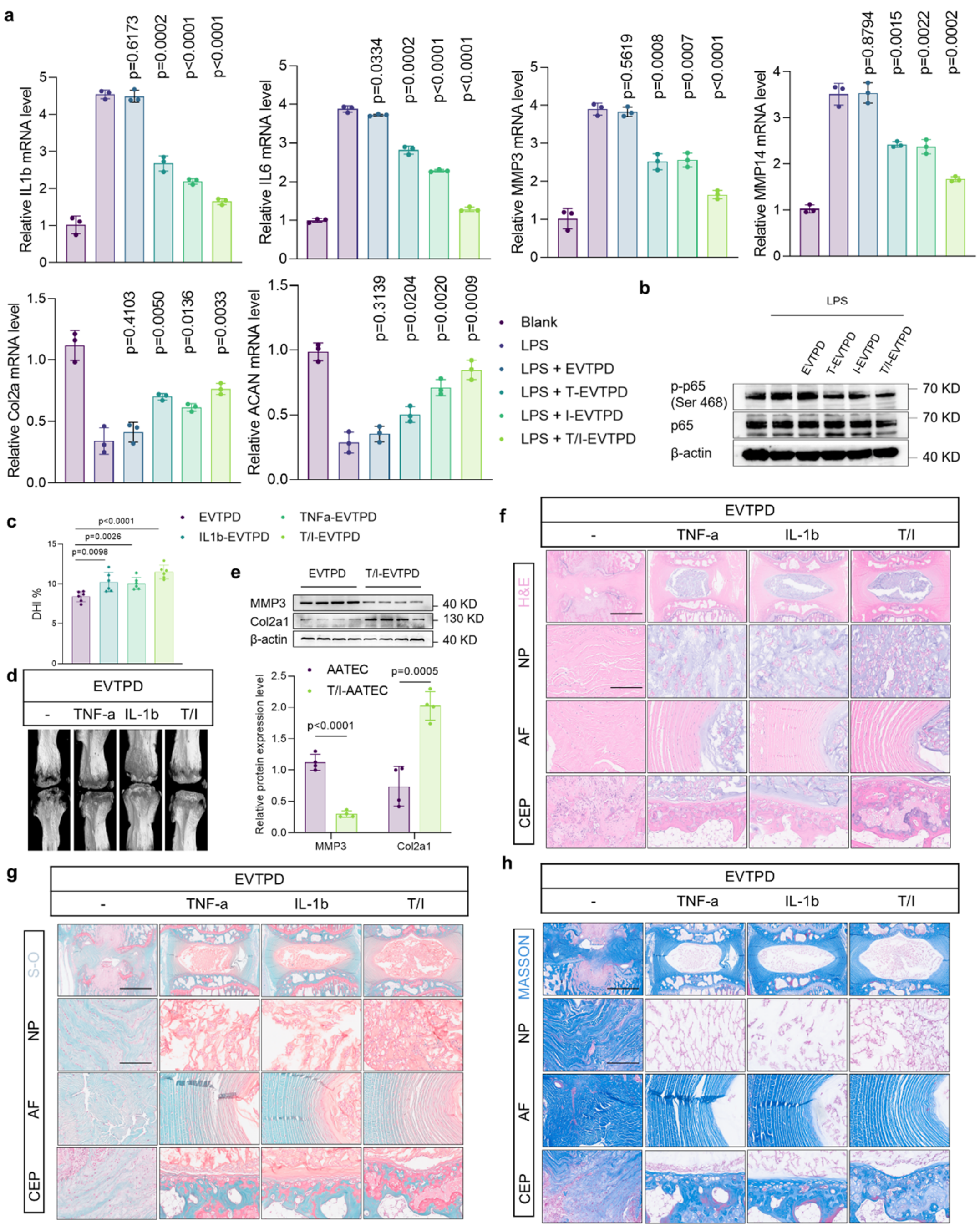

Appendix Figure S4

a The mRNA level of inflammatory cytokines (IL-1b, IL6), matrix metalloproteinases (MMP3, MMP14) and extracellular matrix (collagen II, aggrecan) in LPS pre-conditioned NPCs treated with EVTPD, T-EVTPD, I-EVTPD and T/I-EVTPD for 24 h were detected by Real-time PCR (*n*=3); **b** The level of phosphorylation of p65 in LPS pre-conditioned NPCs treated with EVTPD, T-EVTPD, I-EVTPD and T/I-EVTPD for 24 h were examined by western blot; **c** Quantitative analysis of disc height index (DHI) of intervertebral disc in EVTPD, rIL1b-EVTPD, rTNFa-EVTPD and rT/I-EVTPD groups (*n*=6); **d** Micro-CT imaging of intervertebral disc in EVTPD, rIL1b-EVTPD, rTNFa-EVTPD and rT/I-EVTPD groups; **e** Western blot assay detected the expression of MMP3, Col2a1 of nucleus pulposus tissues in EVTPD and T/I EVTPD groups (*n*=4); **f** HE staining of intervertebral disc in EVTPD, rIL1b-EVTPD, rTNFa-EVTPD and T/I-EVTPD groups (Scale bar:

50 μm; 5 μm); **g** S-O staining of intervertebral disc in EVTPD, rIL1b-EVTPD, rTNFa-EVTPD and T/I-EVTPD groups (Scale bar: 50 μm; 5 μm); **h** Masson staining of intervertebral disc in EVTPD, rIL1b-EVTPD, rTNFa-EVTPD and T/I-EVTPD groups (Scale bar: 50 μm; 5 μm);. Date were analysed by unpaired two-tailed t-tests (**a, c, e**). Data were shown as mean ± SD. Each *n* in **c, e** is an individual rat, *n* in **a** is biological independent samples.

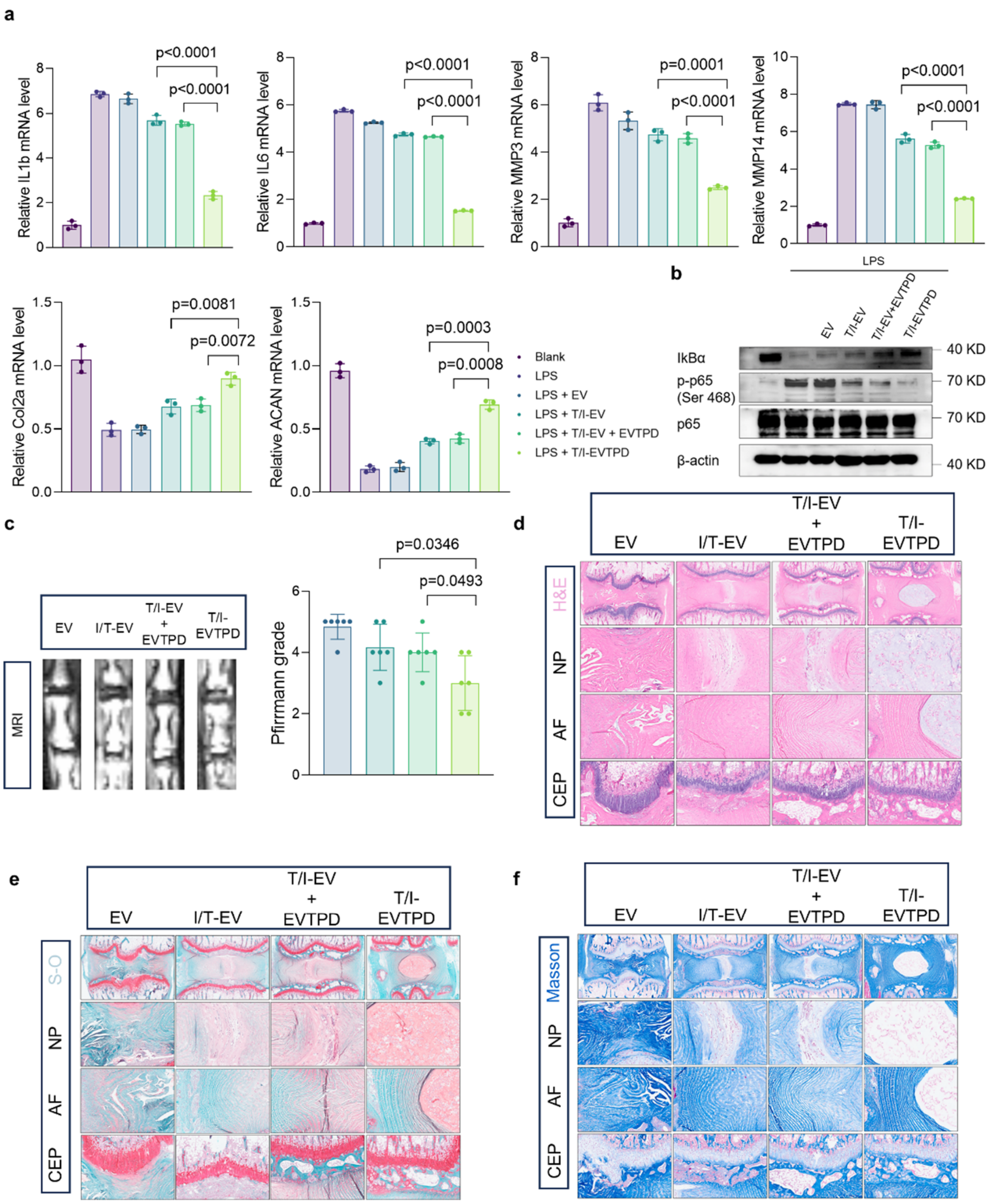

Appendix Figure S5

a The mRNA level of inflammatory cytokines (IL-1b, IL6), matrix metalloproteinases (MMP3, MMP14) and extracellular matrix (collagen II, aggrecan) in LPS pre-conditioned NPCs treated with EV, T/I-EV, mixture of T/I-EV and EVTPD, T/I-EVTPD for 24 h were detected by Real-time PCR (*n*=3); **b** The level of IkBa and phosphorylation of p65 in LPS pre-conditioned NPCs treated with EV, T/I-EV, mixture of T/I-EV and EVTPD, T/I-EVTPD for 24 h were detected by western blot; **c-f** Rat co7/8 IVDs following needle puncture were treated with EVs, rI/T-EVs, mixture of rI/T-EVs and EVTPD, rI/T-EVTPD. MRI images and Piffrrmann grades of rat co7/8 IVDs in different groups were shown in (c) while co8/9 as control (*n*=6); H&E (d), S-O (e), Masson (f) staining of rat IVDs were shown (Scale bar: 50 um; 5um);. Date were analysed by unpaired two-

tailed t-tests (**a, c**). Data were shown as mean ± SD. Each *n* in **c** is an individual rat, *n* in **a** is biological independent samples.

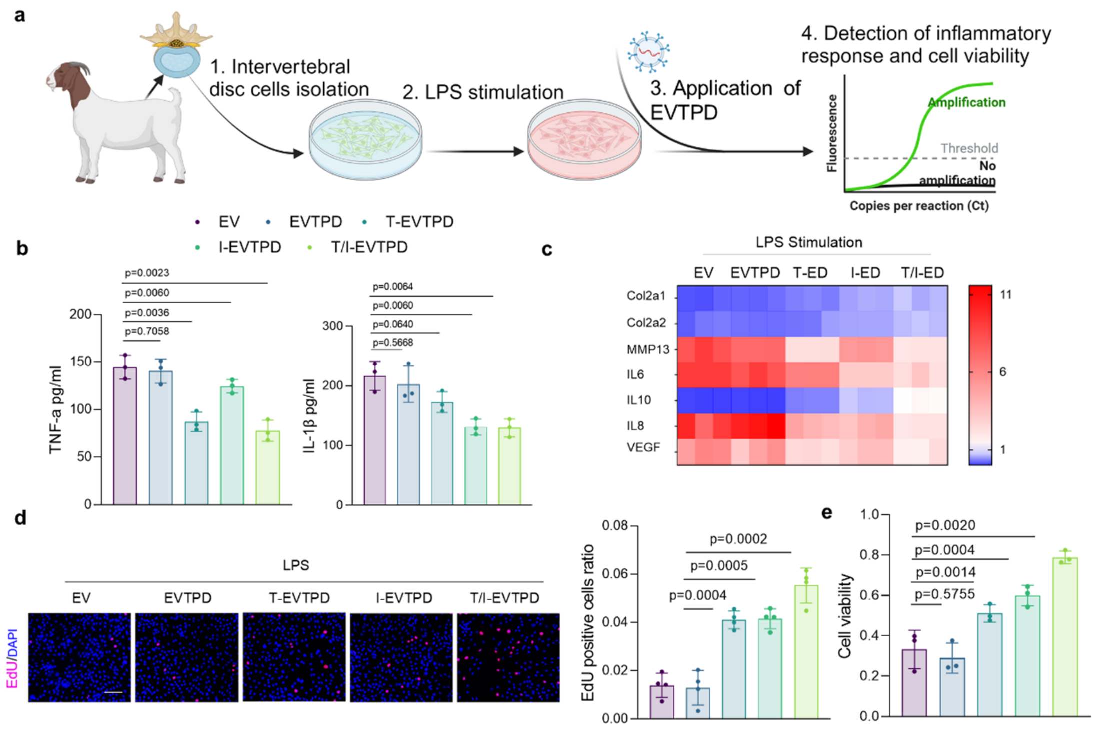

Appendix Figure S6

a Schematic diagram of goat nucleus pulposus cell isolation and experiments *in vitro*. gNPCs were pre-treated with LPS at final concentration of 1 μg/mL for 24h. Then, EVTPDs were applied to treat gNPCs for 24 h before detection of inflammatory responses as well as cell viability **b** ELISA was used to detect the degradation of gTNF-a and gIL-1b in the supernatant of gNPCs after co-incubation with EV, g-EVTPD, gT-EVTPD, gI-EVTPD and gT/I-EVTPD for 24 h (*n*=4); **c** RT-qPCR showed the expression levels of inflammatory cytokines (IL6, IL10, IL8, VEGF) and collagen II (Col2a1, Col2a2) of gNPCs treated with EV, g-EVTPD, gT-EVTPD, gI-EVTPD and gT/I-EVTPD for 24 h (*n*=3); **d** EdU assay was applied to detect the proliferation levels of gNPCs in each treated groups (*n*=4) (Scale bar: 10 um); **e** CCK8 assay was used to monitor the cell viability of NPCs in each treated groups (*n*=3). Date were analysed by unpaired two-tailed t-tests (**b, d, e**). Data were shown as mean ± SD. Each *n* in **b, c, d, e** is biological independent samples. Schematics in **a** was created using BioRender (BioRender. com).

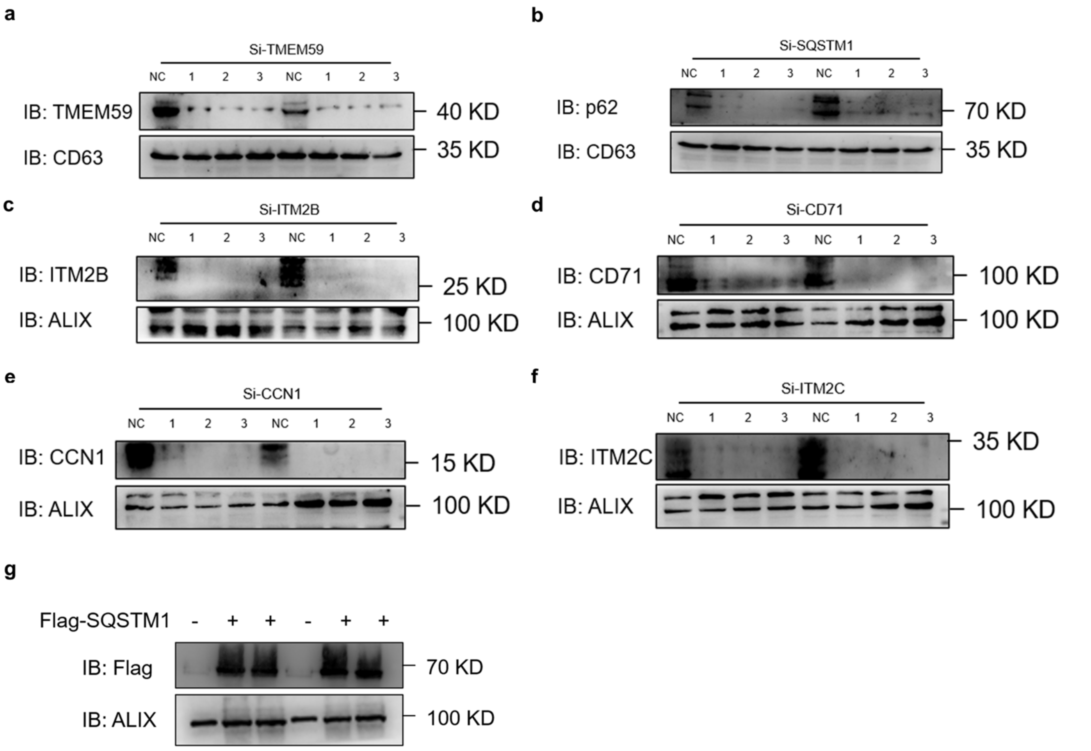

Appendix Figure S7

a-f Western blot assay detected knockdown efficiency in shRNA-pretreated HEK293T derived EVs; **g** Western blot assay detected overexpression of Flag-SQSTM1 in HEK293T derived EVs. **h** Western blot assay detected knockdown of MAP1LC3B in NPCs.

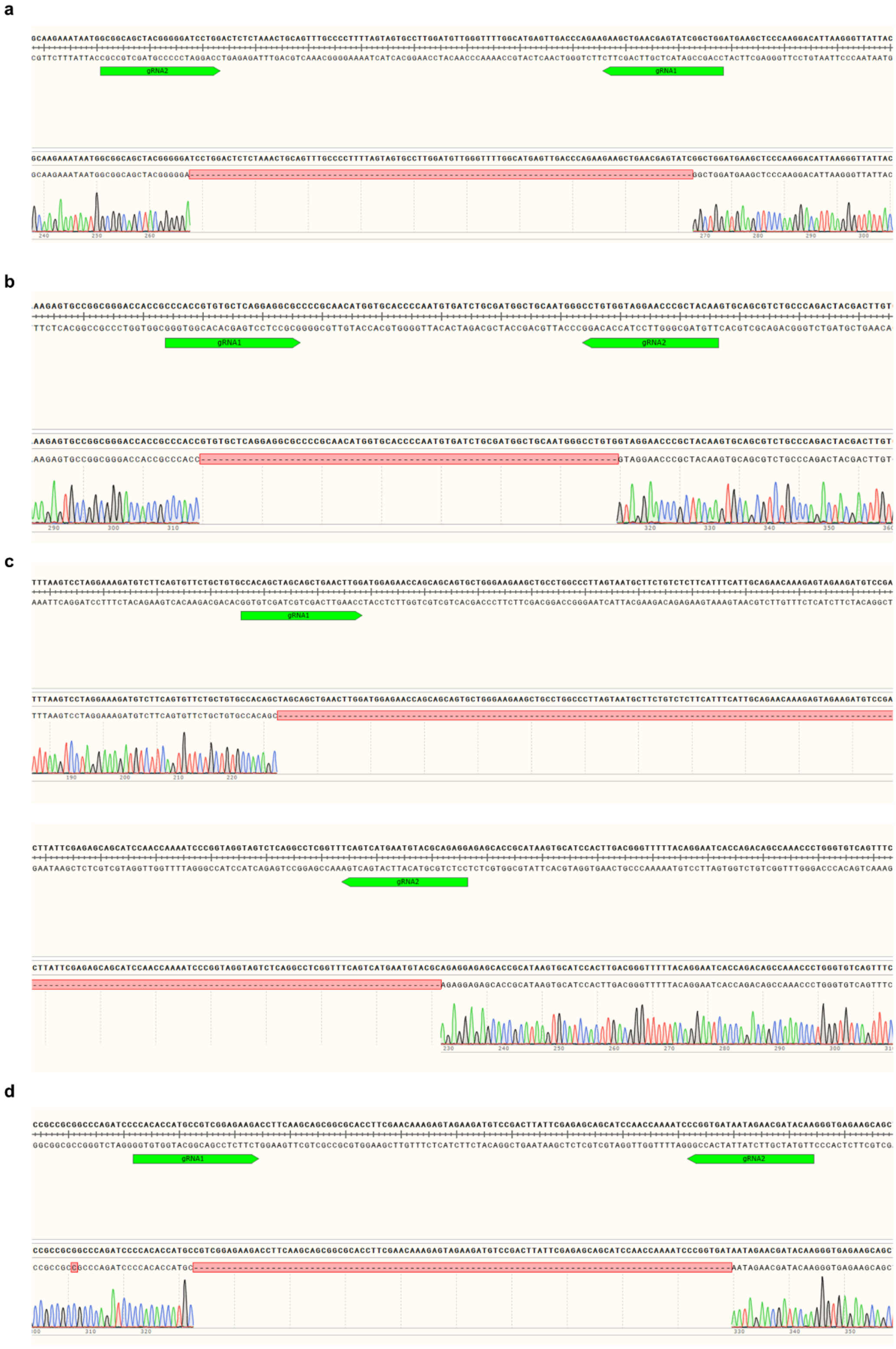

Appendix Figure S8

a-d Sanger sequencing of the knockout fragment of ATG7 KO (a), SQSTM1 KO (b), MAP1LC3B1 (c), MAP1LC3B2 KO (b) cell lines. The corresponding wild-type DNA sequences were aligned.

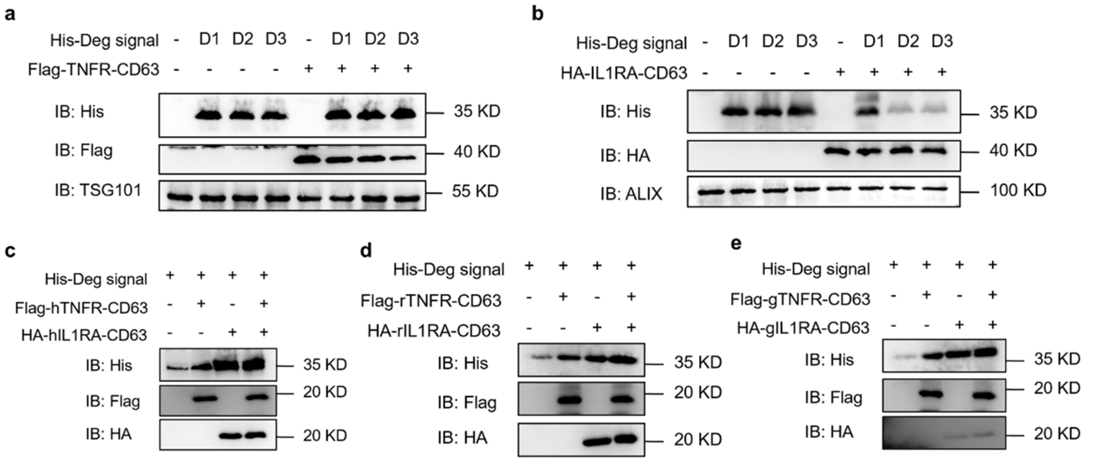

Appendix Figure S9

a Constructing of EVTPD targeting human TNF-a fused with different degradation signals; **b** Constructing of EVTPD targeting human IL-1b fused with different degradation signals; **c** Constructing of EVTPD targeting human TNF-a and IL-1b; **d** Constructing of EVTPD targeting rat TNF-a and IL-1b; **e** Constructing of degradation EVs targeting goat TNF-a and IL-1b.
