# Supplementary figures and images for: Extracellular vesicle-based targeted protein degradation platform for multiple extracellular proteins (part 1 of 2)
Source: EMBO Mol Med. 2026 Jan 12;18(2):759–94. doi: 10.1038/s44321-025-00371-8 (PMC12905291; doi:10.1038/s44321-025-00371-8)

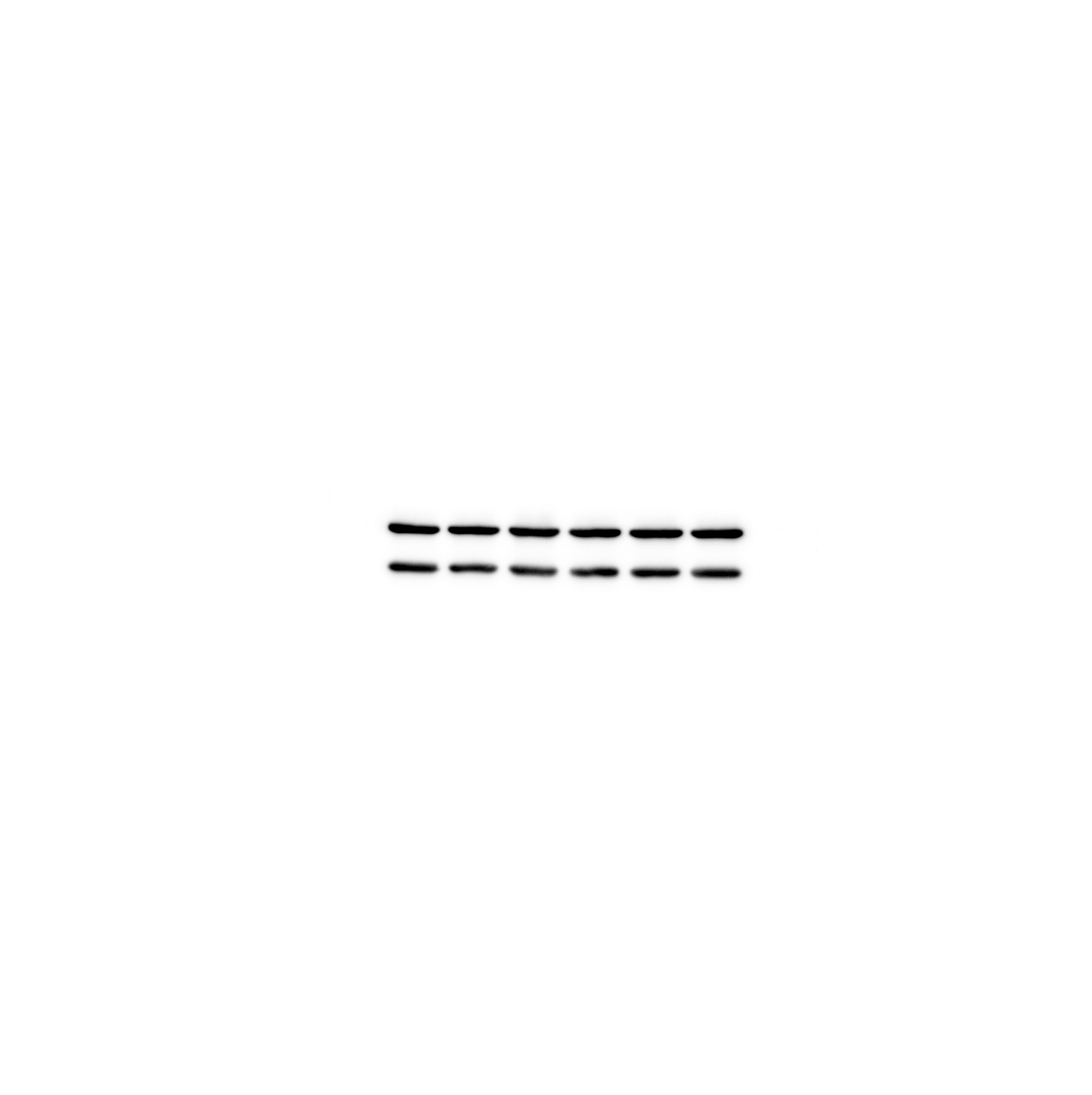

Supplement: Supplementary file 7 — Source data Fig. 1 [file 44321_2025_371_MOESM7_ESM.zip › Figure 1/Fig. 1d/Fig. 1d Deg-actin.tif]

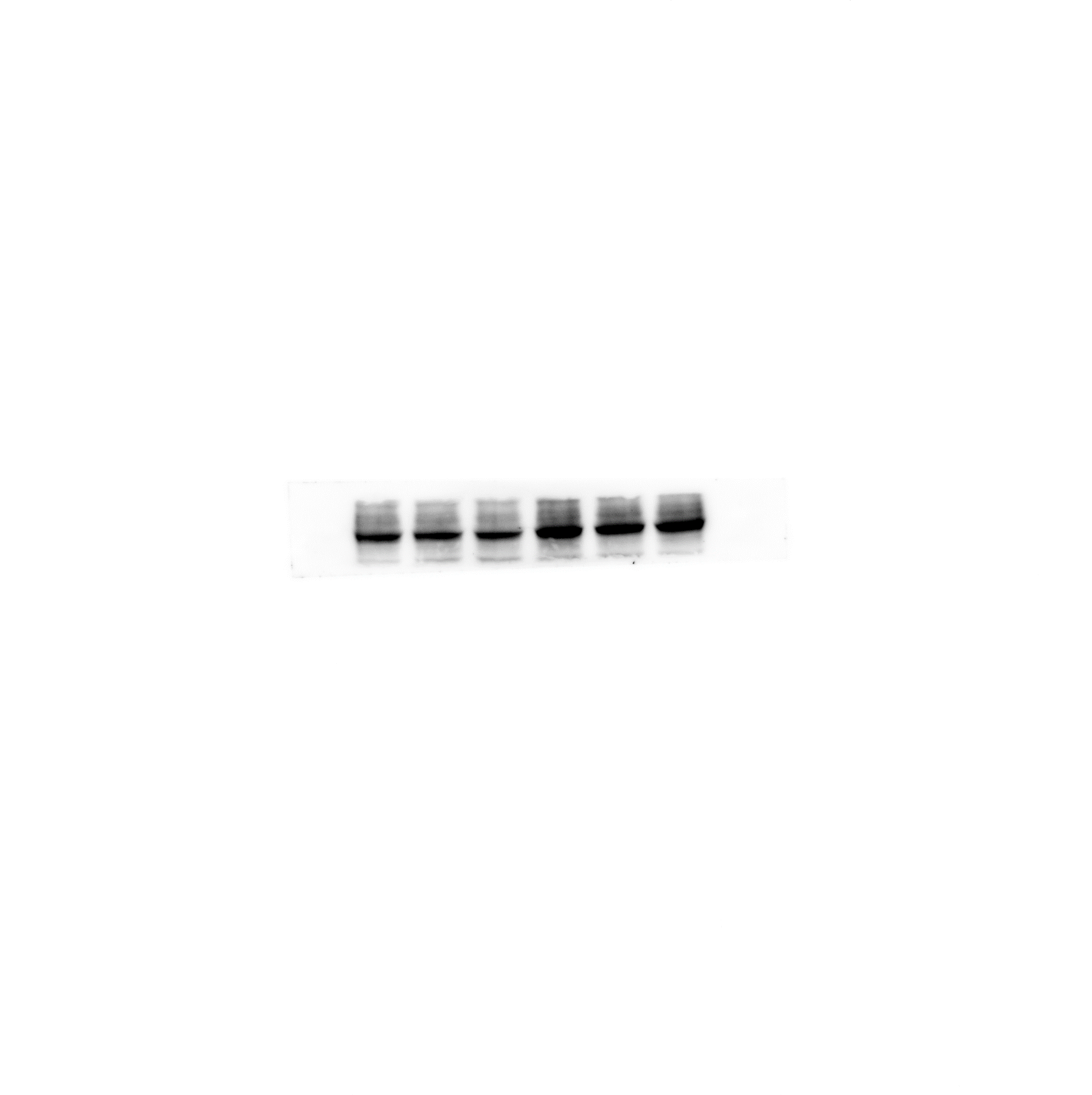

Supplement: Supplementary file 7 — Source data Fig. 1 [file 44321_2025_371_MOESM7_ESM.zip › Figure 1/Fig. 1d/Fig. 1d Deg-GFP.tif]

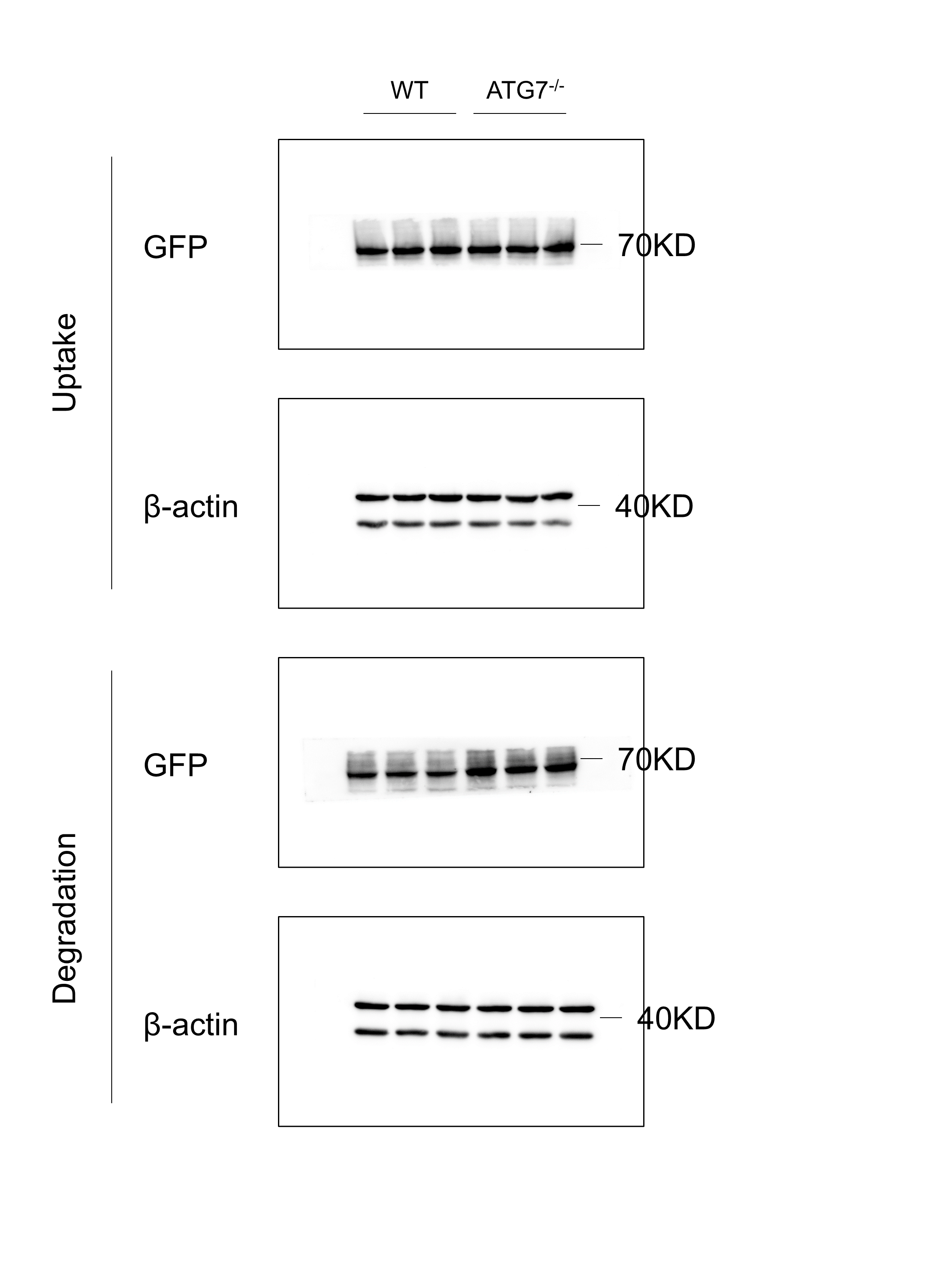

Supplement: Supplementary file 7 — Source data Fig. 1 [file 44321_2025_371_MOESM7_ESM.zip › Figure 1/Fig. 1d/Fig. 1d summary plus label.tif]

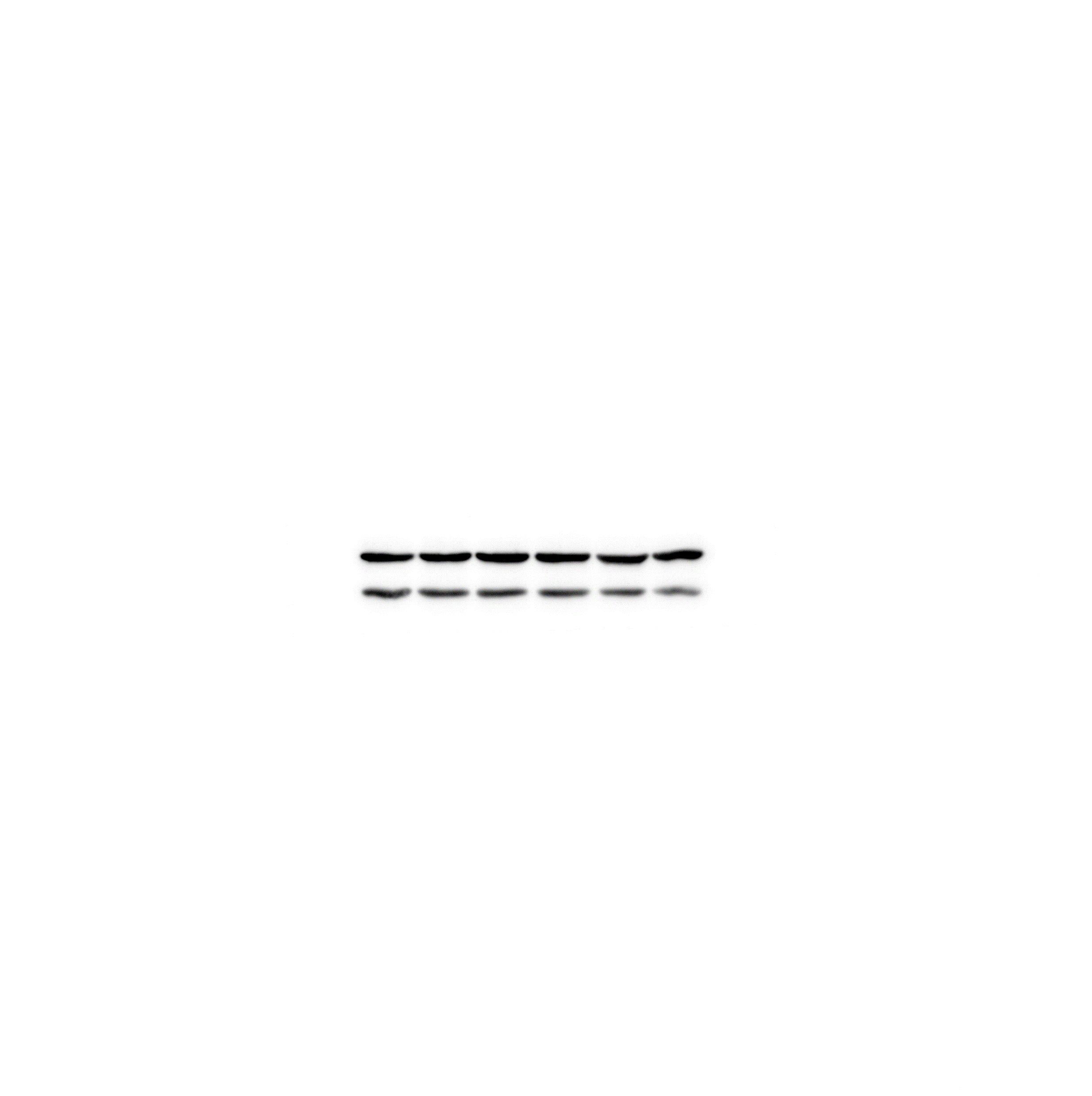

Supplement: Supplementary file 7 — Source data Fig. 1 [file 44321_2025_371_MOESM7_ESM.zip › Figure 1/Fig. 1d/Fig. 1d Uptake-actin.tif]

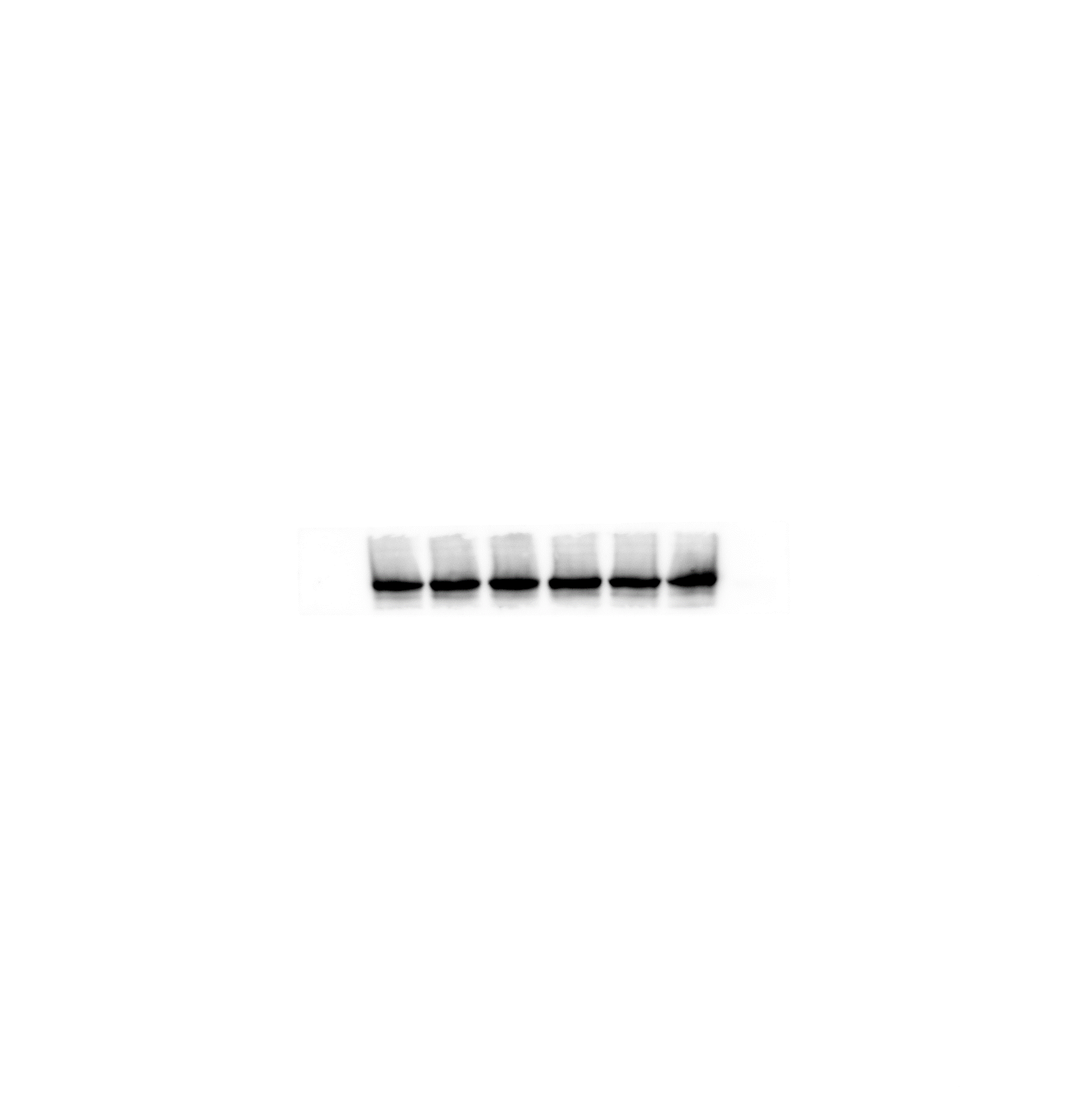

Supplement: Supplementary file 7 — Source data Fig. 1 [file 44321_2025_371_MOESM7_ESM.zip › Figure 1/Fig. 1d/Fig. 1d Uptake-GFP.tif]

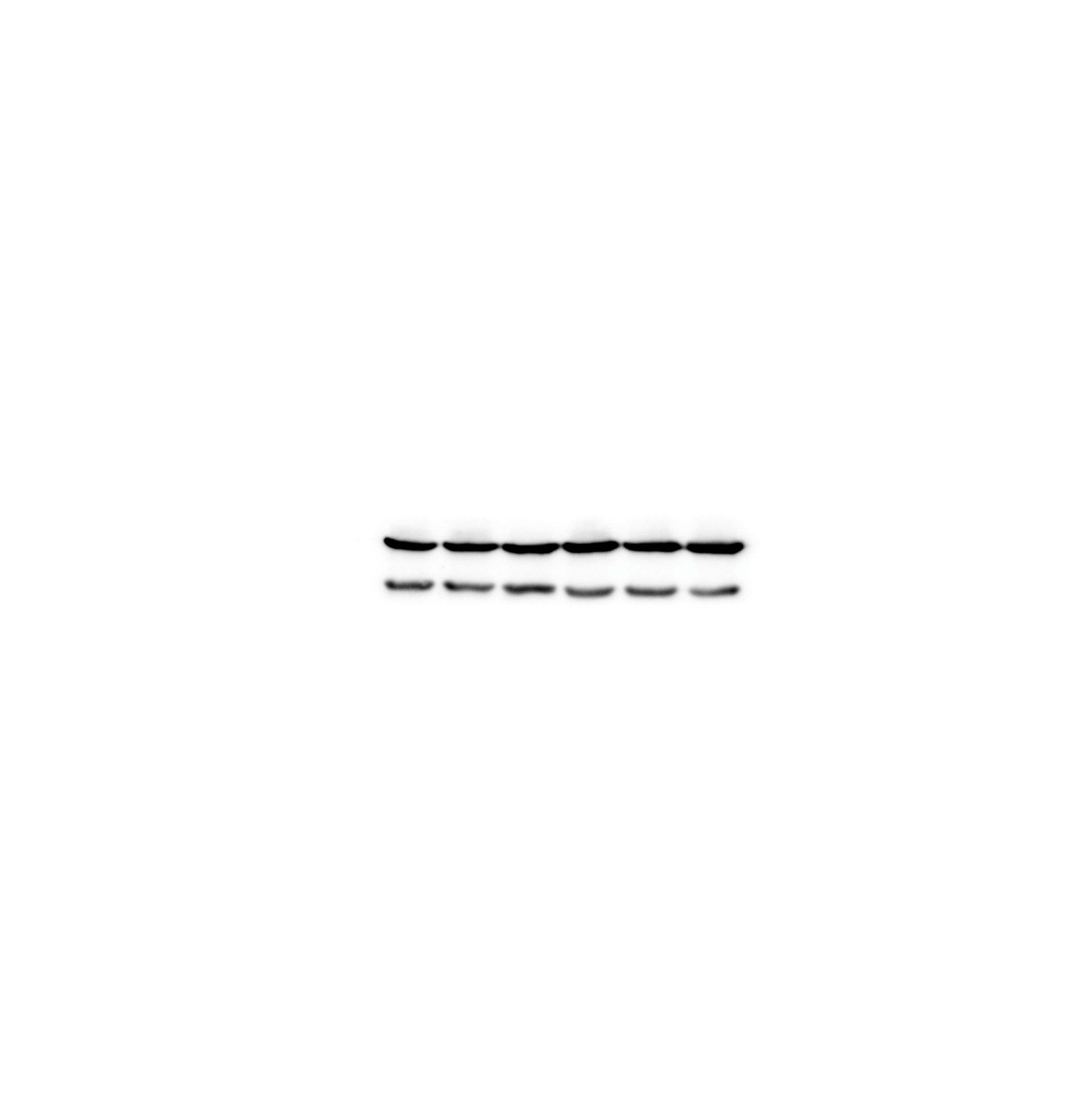

Supplement: Supplementary file 7 — Source data Fig. 1 [file 44321_2025_371_MOESM7_ESM.zip › Figure 1/Fig. 1e/Fig. 1e Cell lysate-actin.tif]

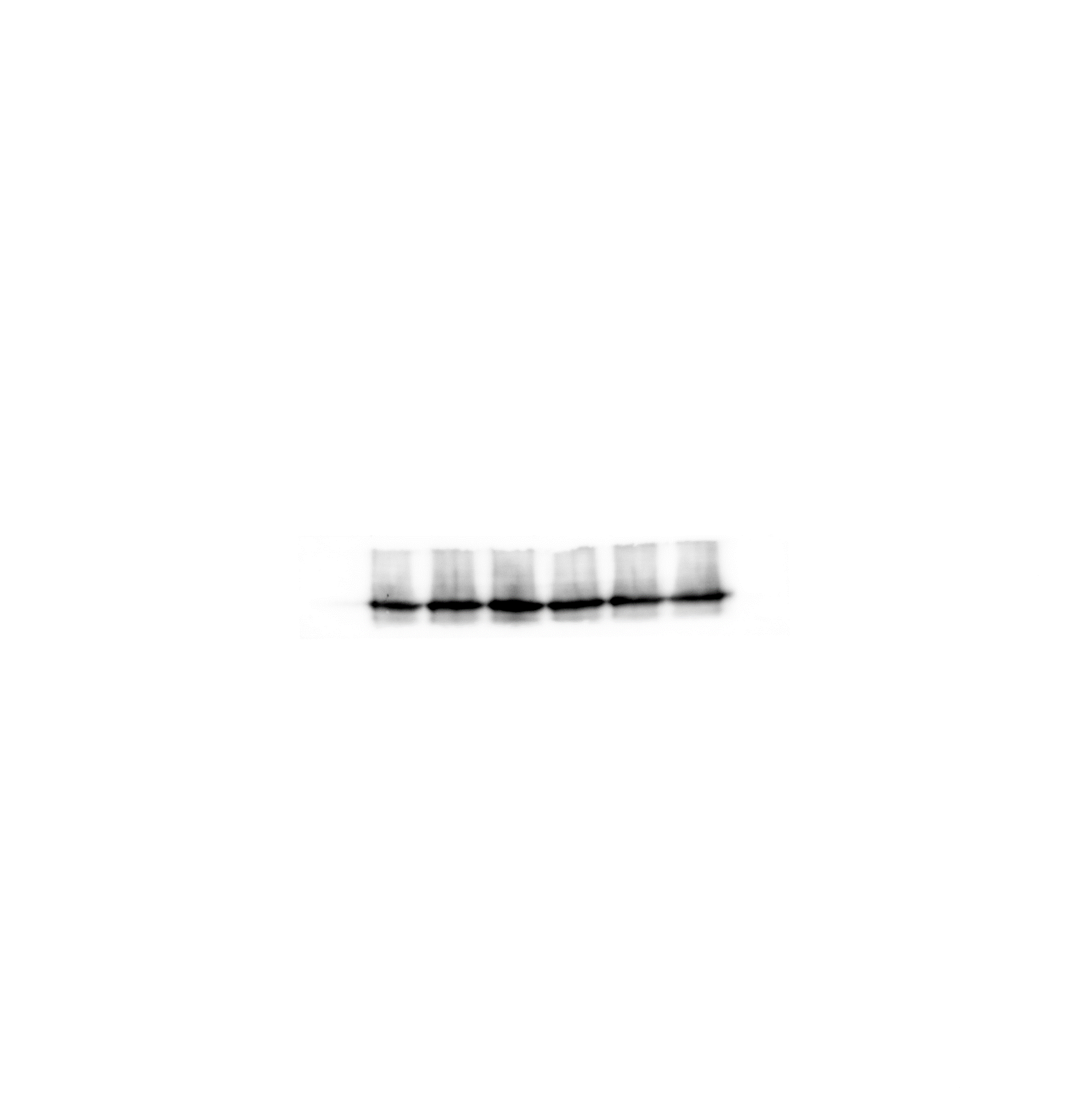

Supplement: Supplementary file 7 — Source data Fig. 1 [file 44321_2025_371_MOESM7_ESM.zip › Figure 1/Fig. 1e/Fig. 1e Cell lysate-GFP.tif]

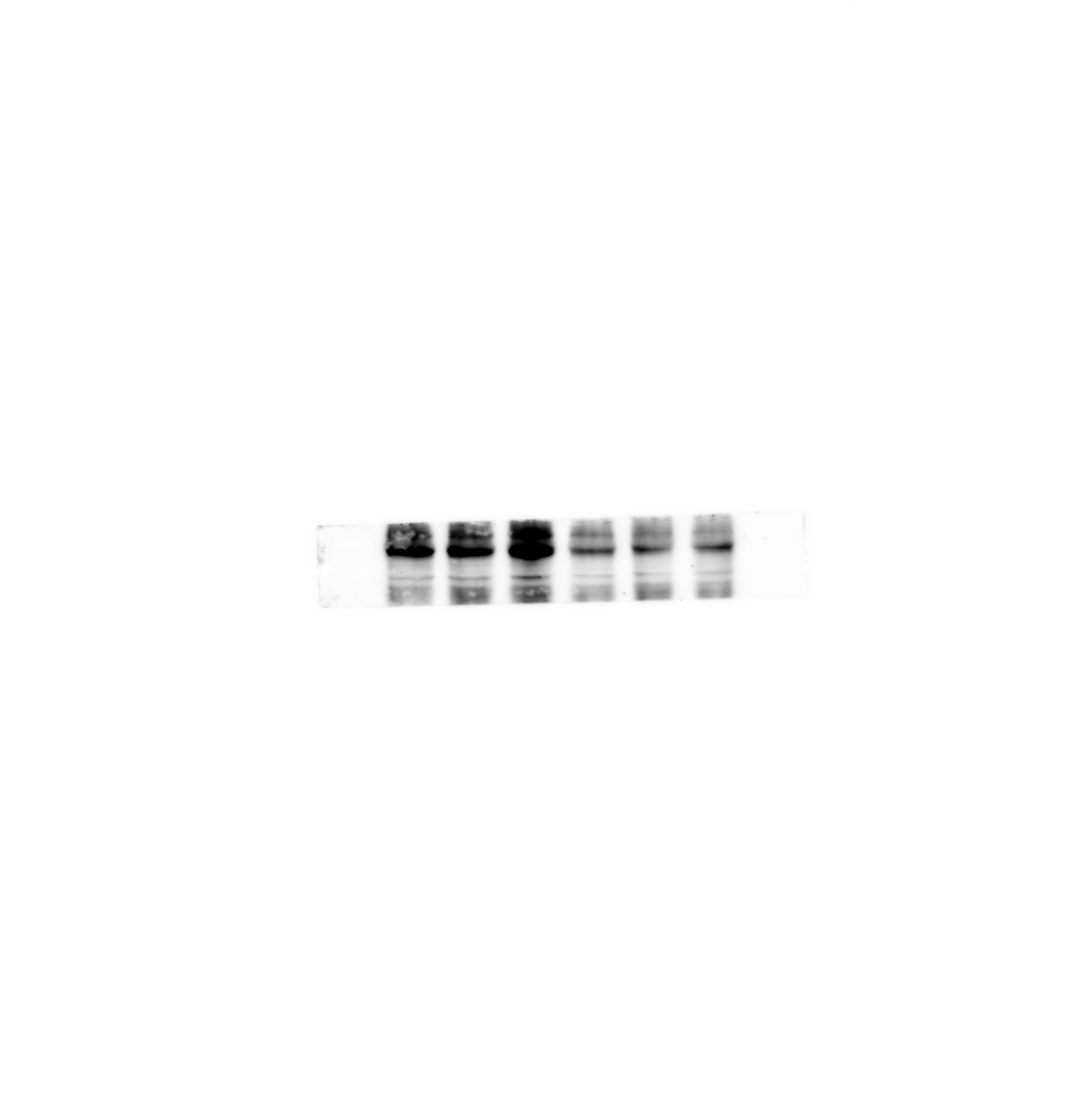

Supplement: Supplementary file 7 — Source data Fig. 1 [file 44321_2025_371_MOESM7_ESM.zip › Figure 1/Fig. 1e/Fig. 1e Lysosome-GFP.tif]

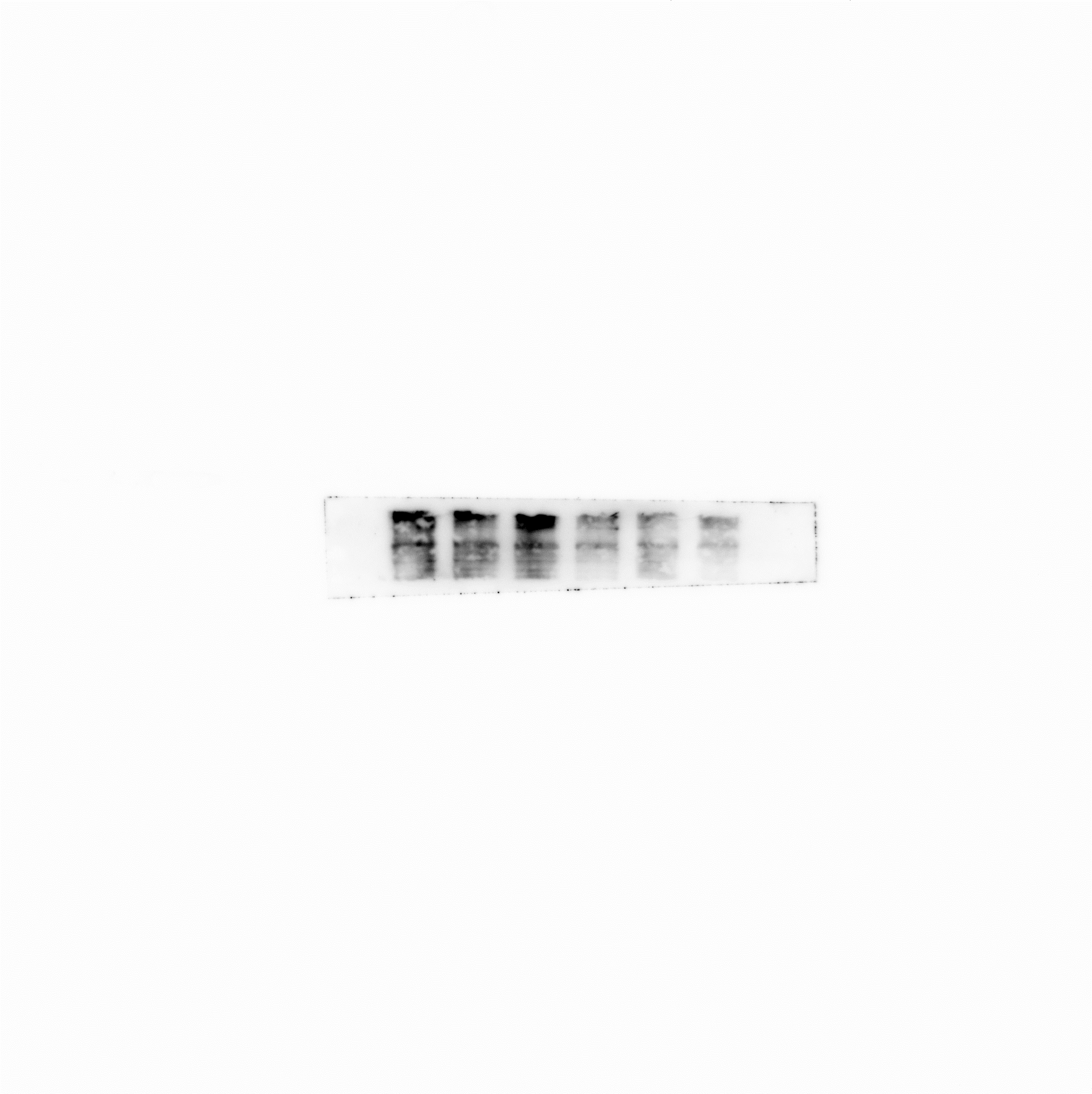

Supplement: Supplementary file 7 — Source data Fig. 1 [file 44321_2025_371_MOESM7_ESM.zip › Figure 1/Fig. 1e/Fig. 1e Lysosome-LampII.tif]

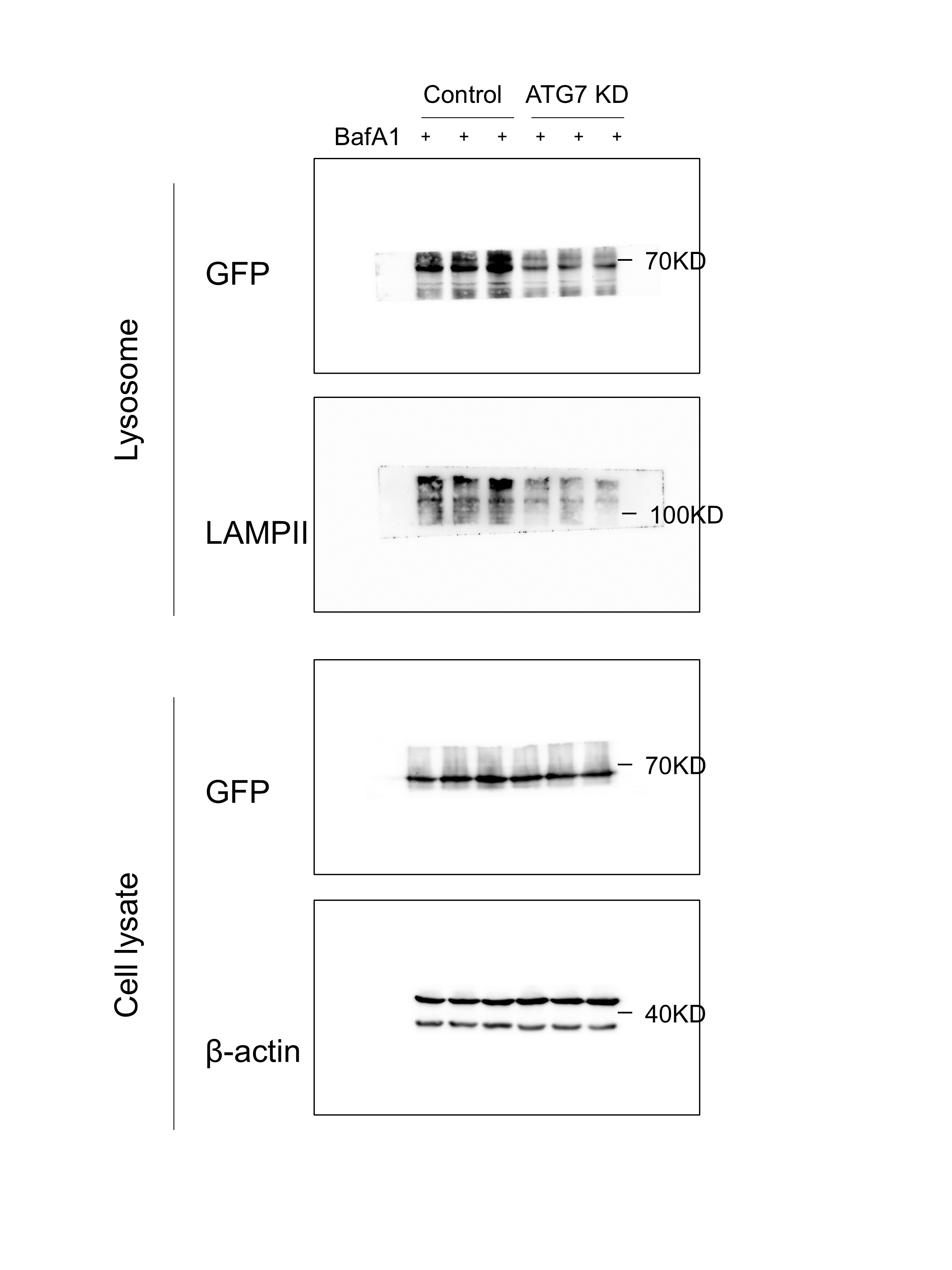

Supplement: Supplementary file 7 — Source data Fig. 1 [file 44321_2025_371_MOESM7_ESM.zip › Figure 1/Fig. 1e/Fig. 1e summary plus label .tif]

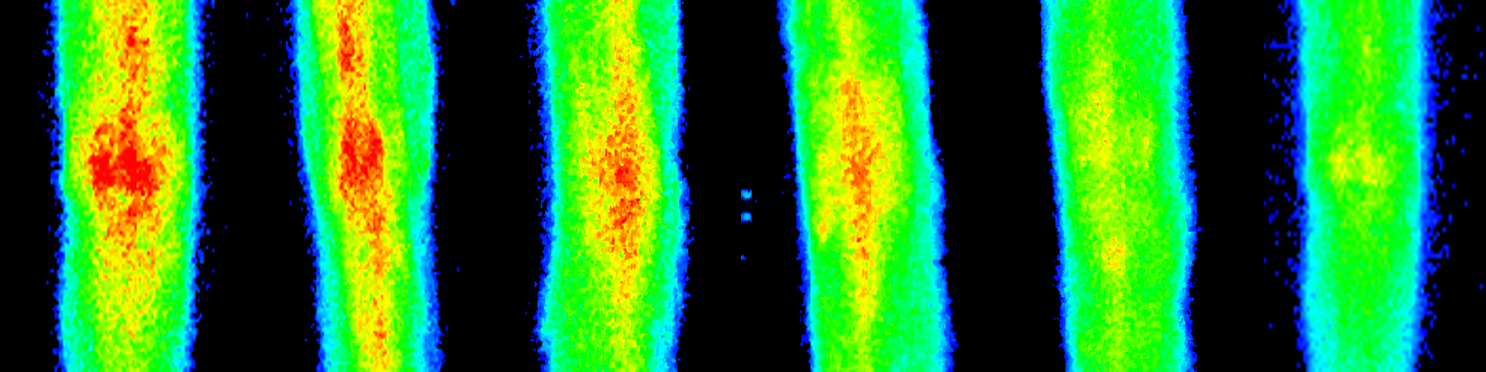

Supplement: Supplementary file 7 — Source data Fig. 1 [file 44321_2025_371_MOESM7_ESM.zip › Figure 1/Fig. 1f/3-MA 0-20d.tif]

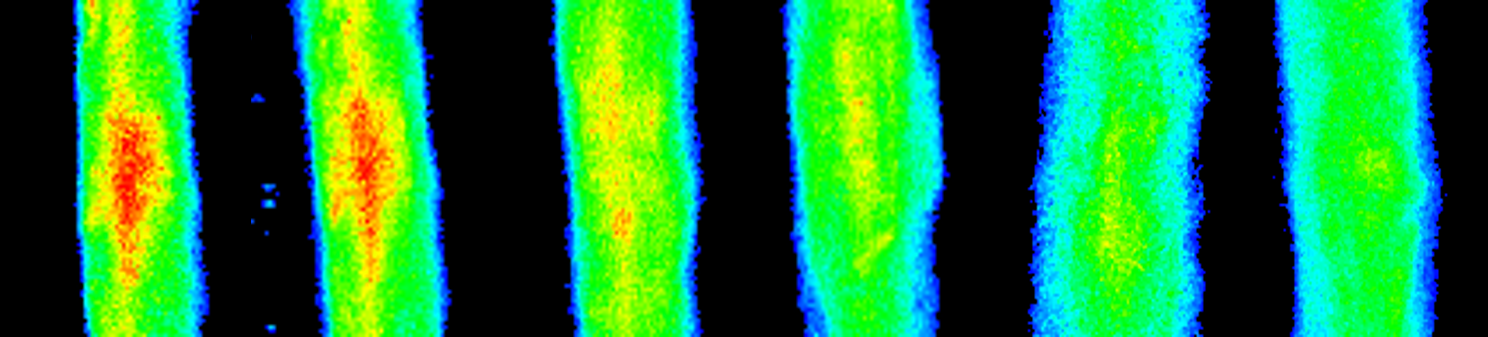

Supplement: Supplementary file 7 — Source data Fig. 1 [file 44321_2025_371_MOESM7_ESM.zip › Figure 1/Fig. 1f/Control 0-20d.tif]

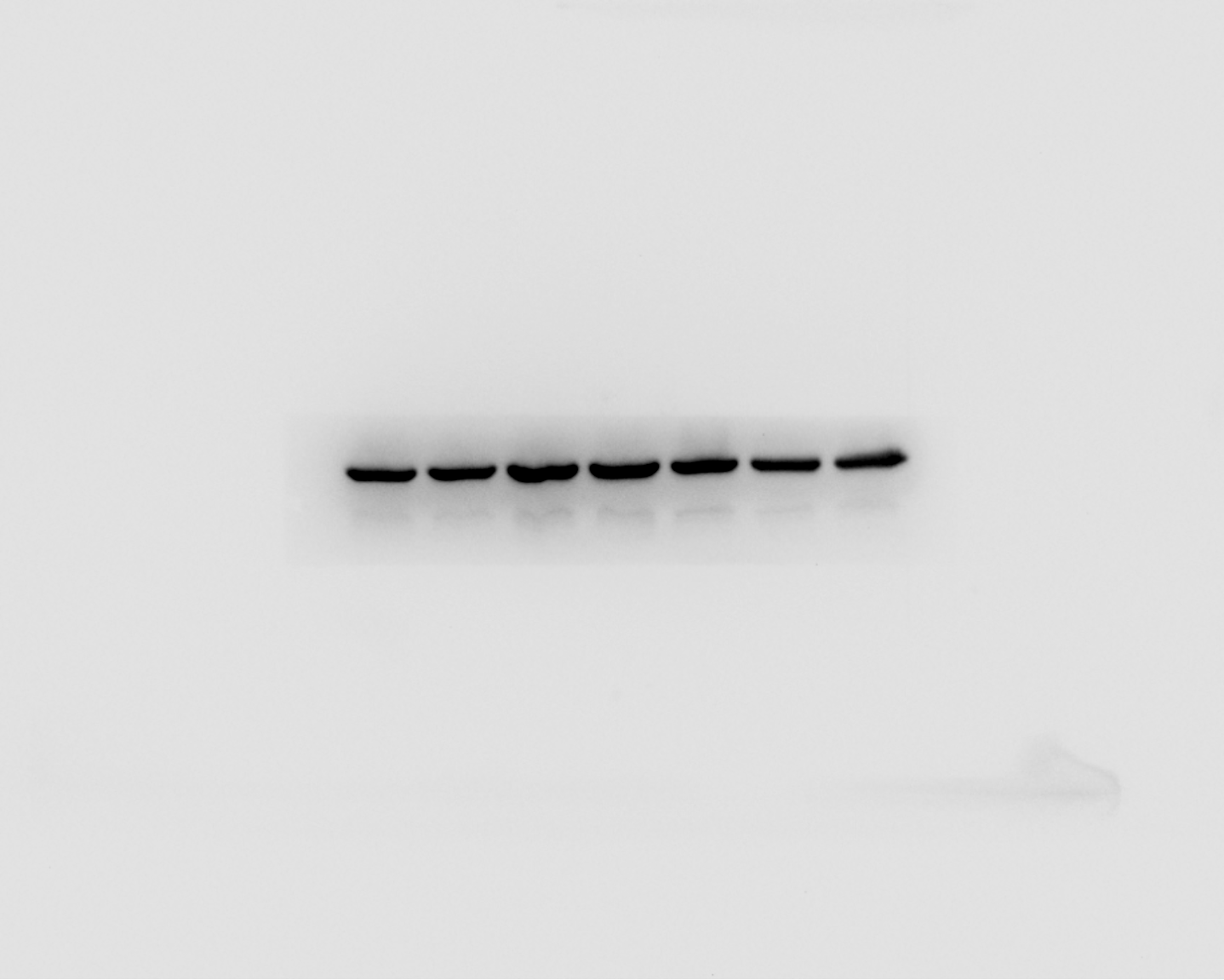

Supplement: Supplementary file 8 — Source data Fig. 2 [file 44321_2025_371_MOESM8_ESM.zip › Figure 2/Fig. 2e/Fig. 2e actin.tif]

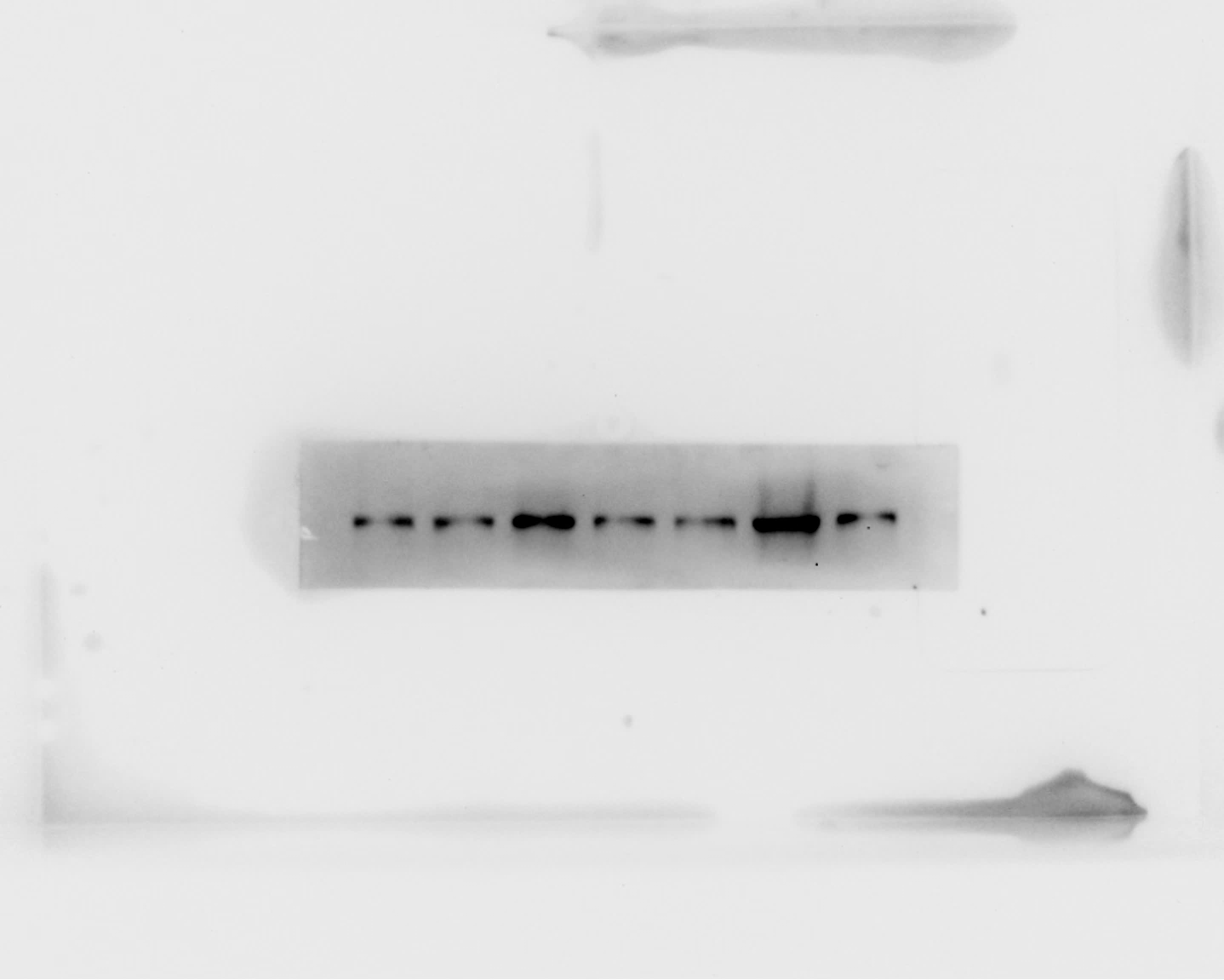

Supplement: Supplementary file 8 — Source data Fig. 2 [file 44321_2025_371_MOESM8_ESM.zip › Figure 2/Fig. 2e/Fig. 2e GFP.tif]

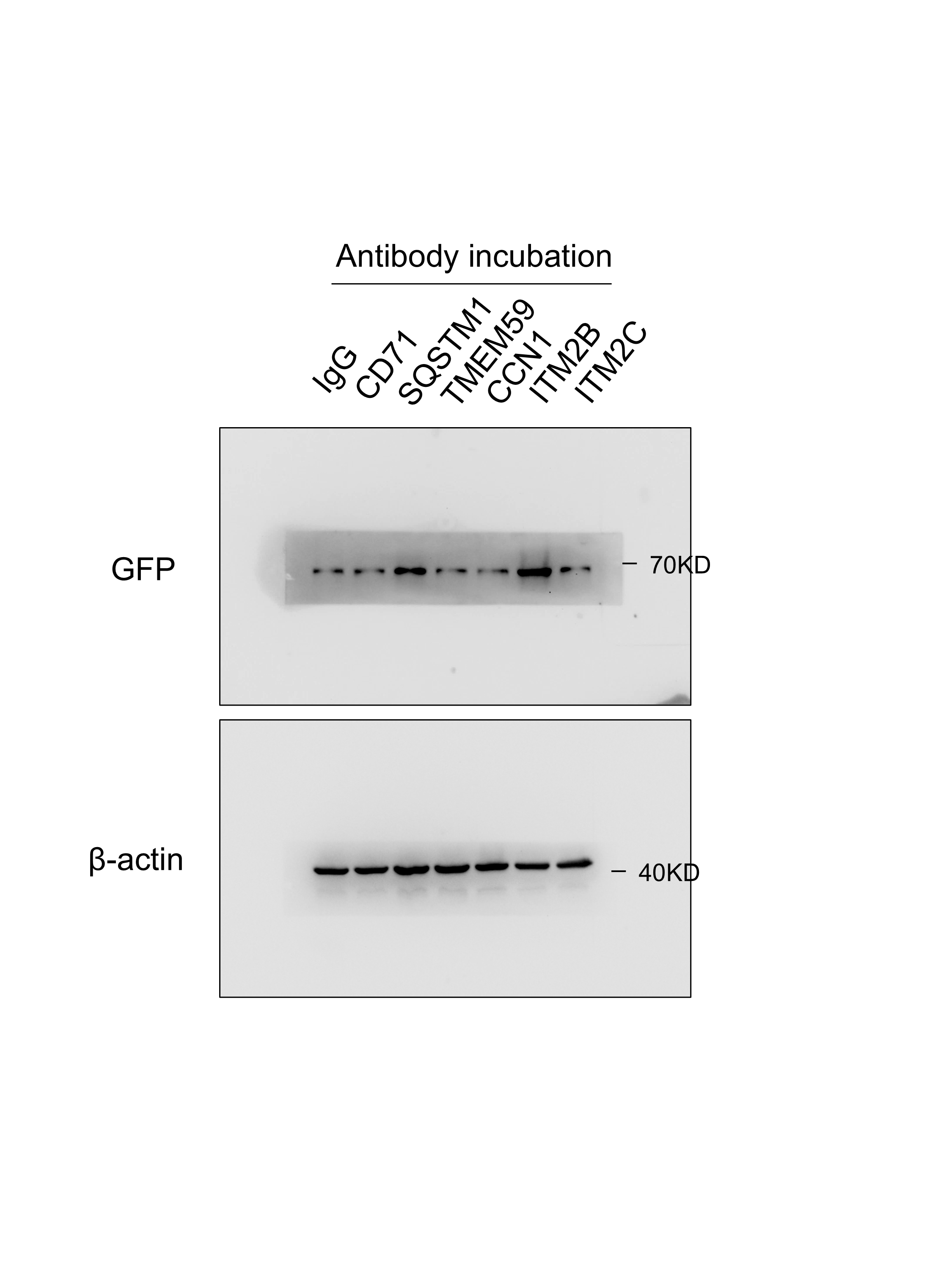

Supplement: Supplementary file 8 — Source data Fig. 2 [file 44321_2025_371_MOESM8_ESM.zip › Figure 2/Fig. 2e/Fig. 2e summary plus label.tif]

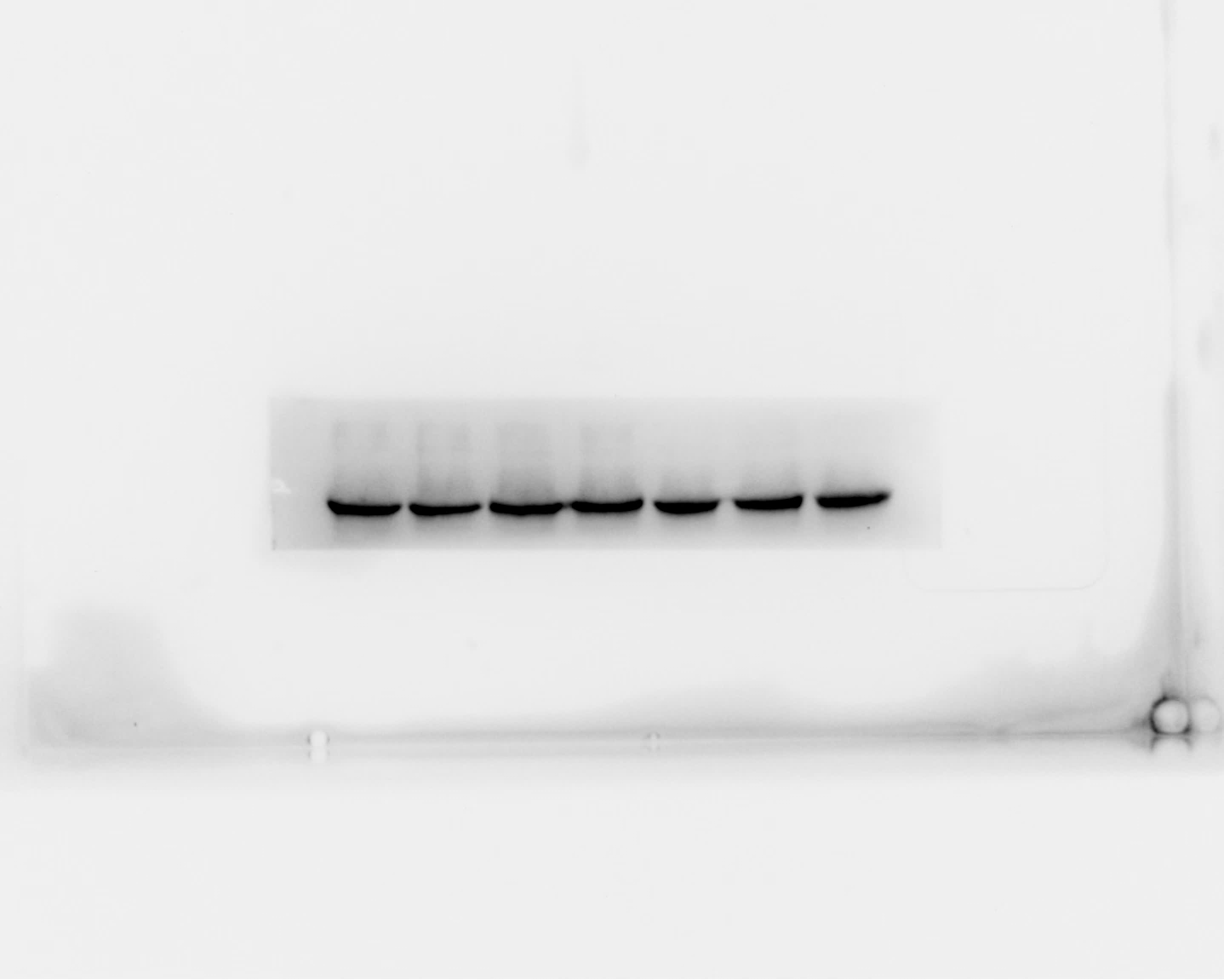

Supplement: Supplementary file 8 — Source data Fig. 2 [file 44321_2025_371_MOESM8_ESM.zip › Figure 2/Fig. 2f/Fig. 2f actin.tif]

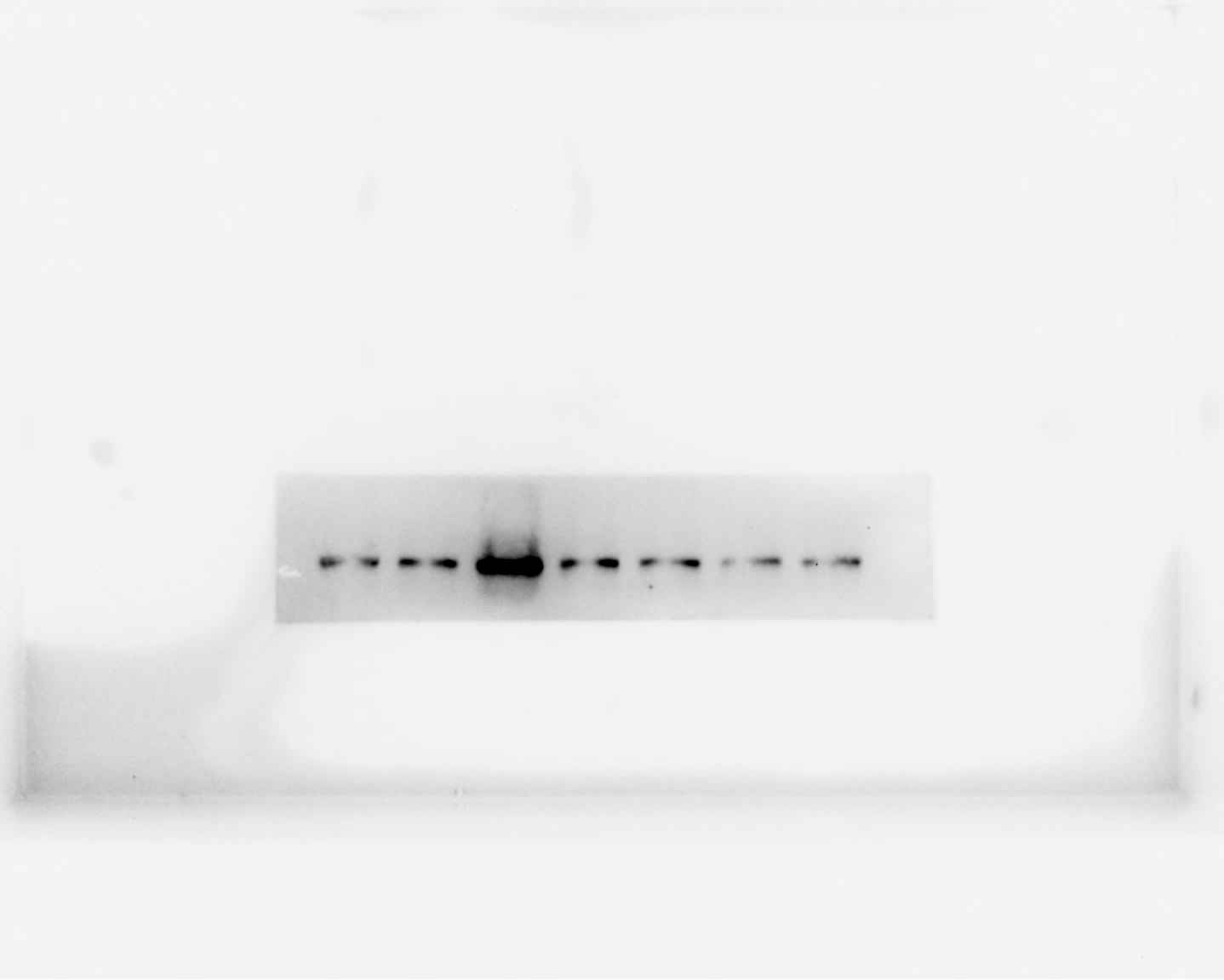

Supplement: Supplementary file 8 — Source data Fig. 2 [file 44321_2025_371_MOESM8_ESM.zip › Figure 2/Fig. 2f/Fig. 2f GFP.tif]

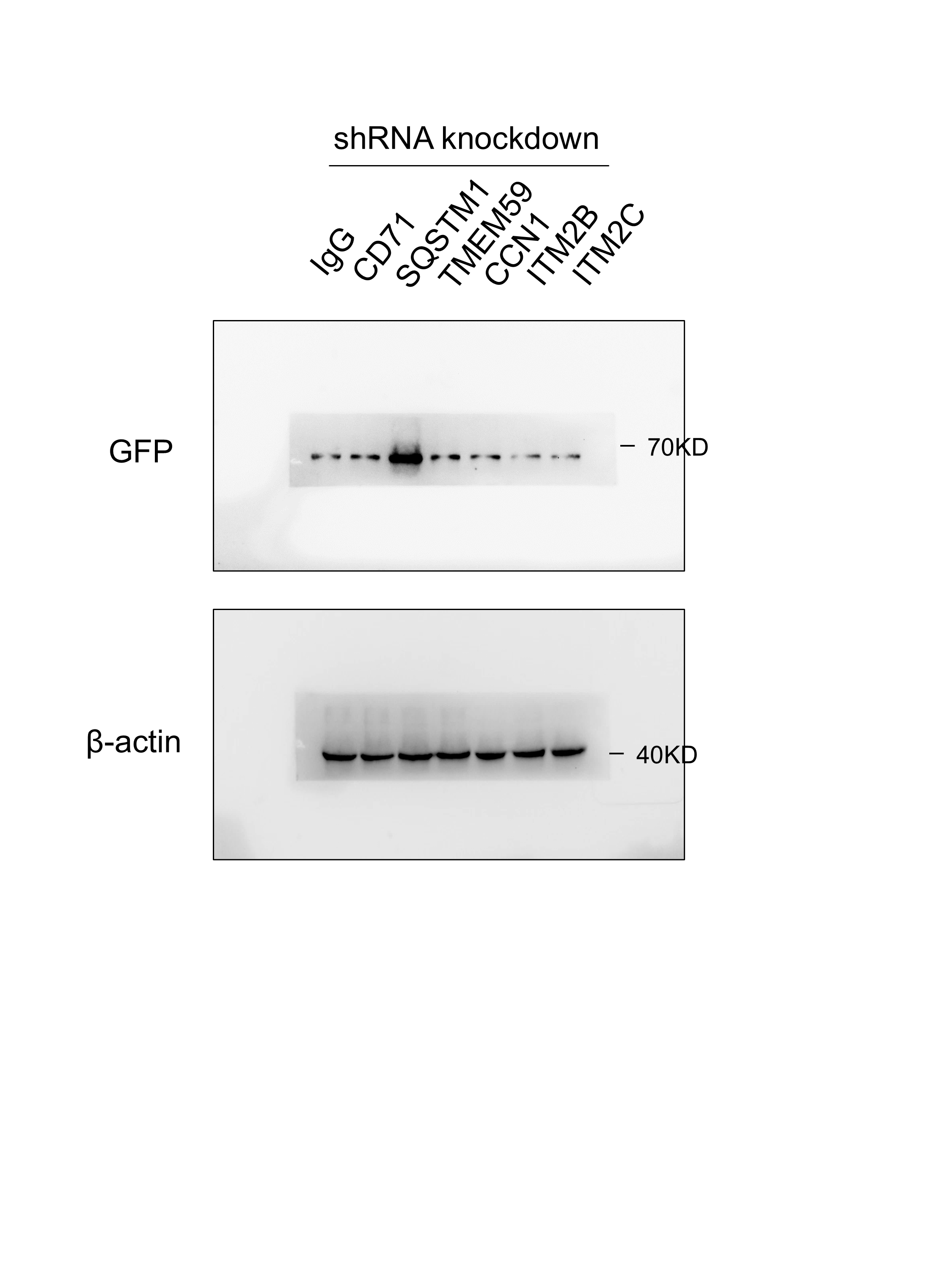

Supplement: Supplementary file 8 — Source data Fig. 2 [file 44321_2025_371_MOESM8_ESM.zip › Figure 2/Fig. 2f/Fig. 2f summary plus label.tif]

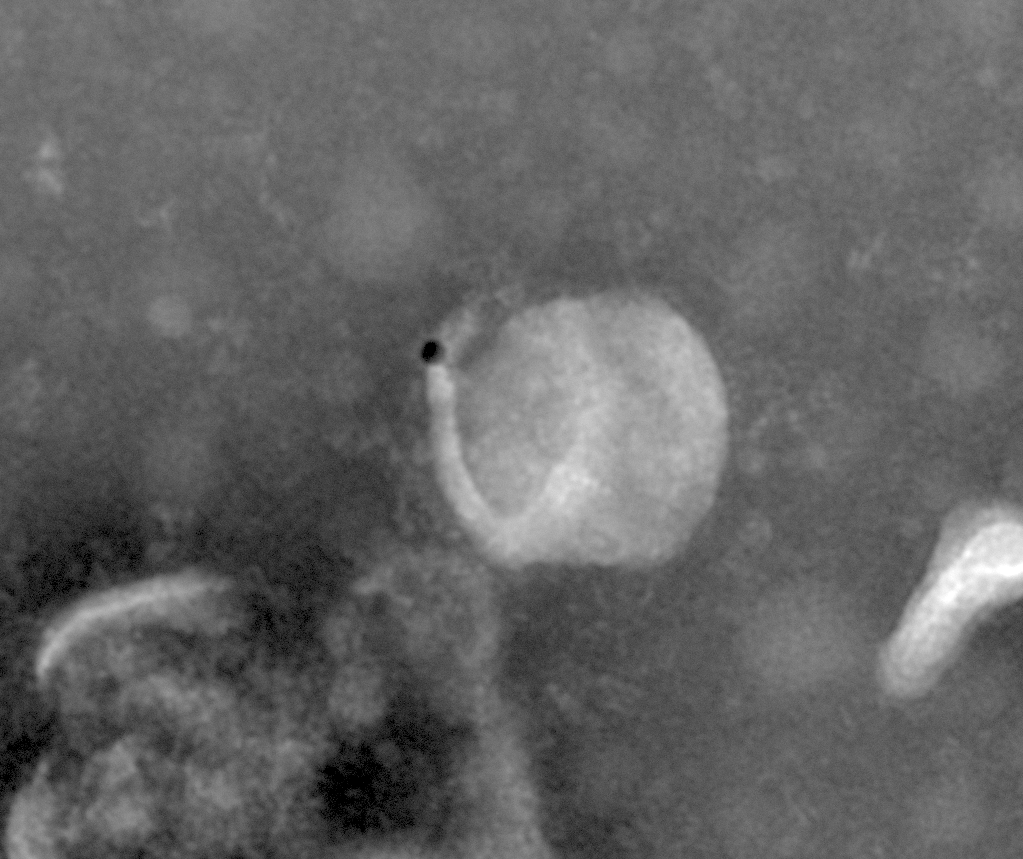

Supplement: Supplementary file 8 — Source data Fig. 2 [file 44321_2025_371_MOESM8_ESM.zip › Figure 2/Fig. 2g/Fig. 2g.tif]

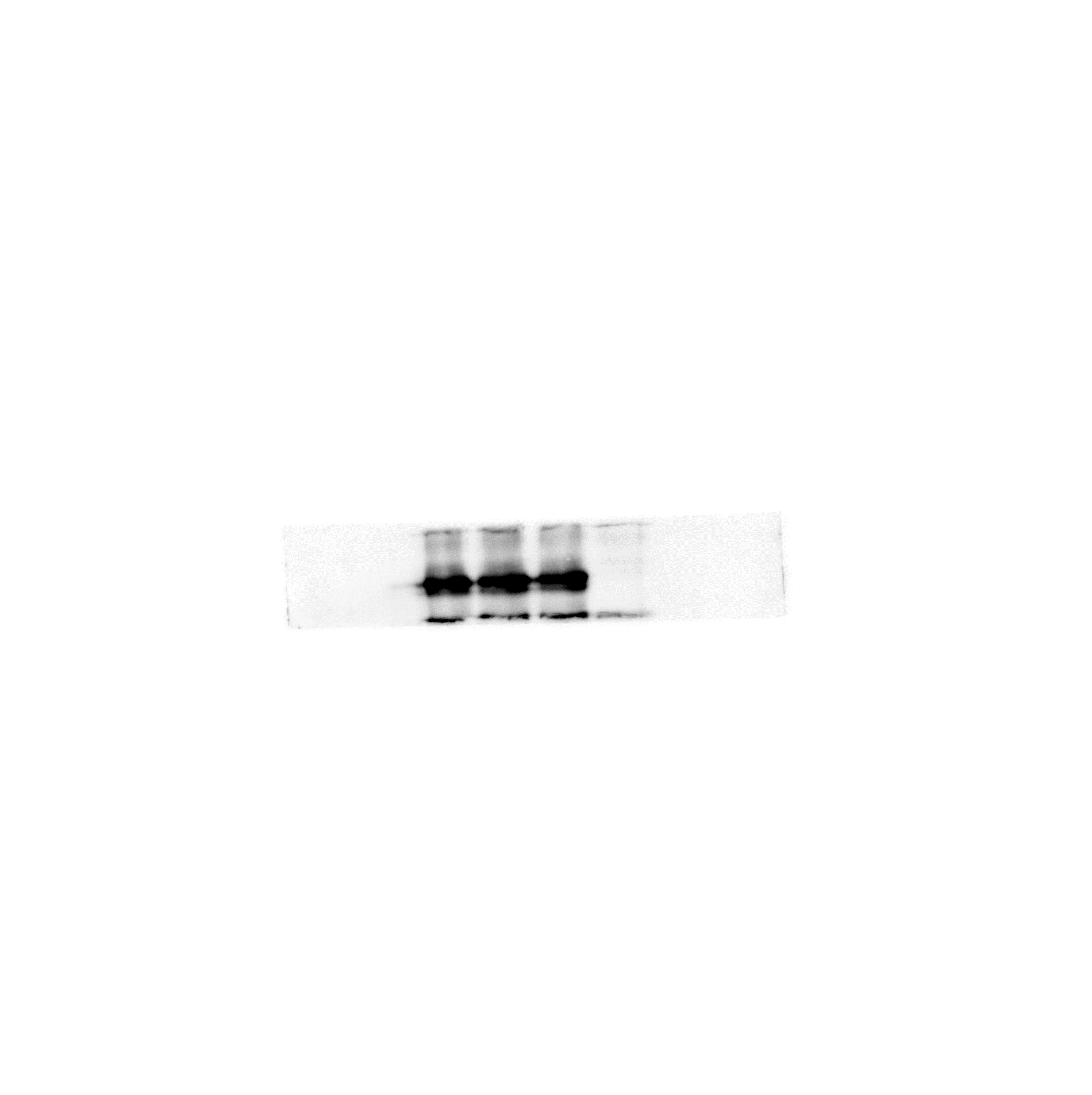

Supplement: Supplementary file 8 — Source data Fig. 2 [file 44321_2025_371_MOESM8_ESM.zip › Figure 2/Fig. 2h/Fig. 2h ALIX.tif]

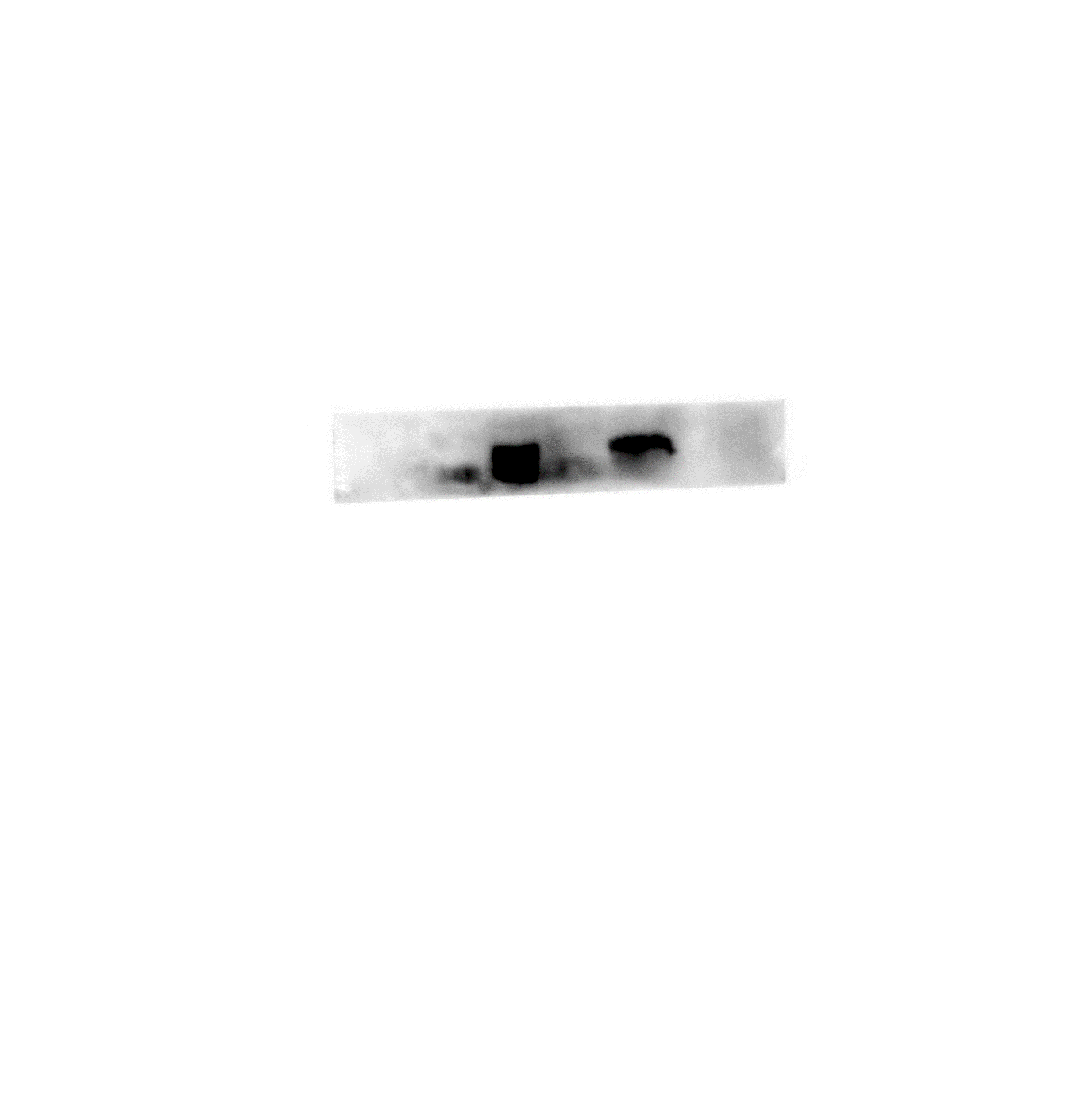

Supplement: Supplementary file 8 — Source data Fig. 2 [file 44321_2025_371_MOESM8_ESM.zip › Figure 2/Fig. 2h/Fig. 2h CD63.tif]

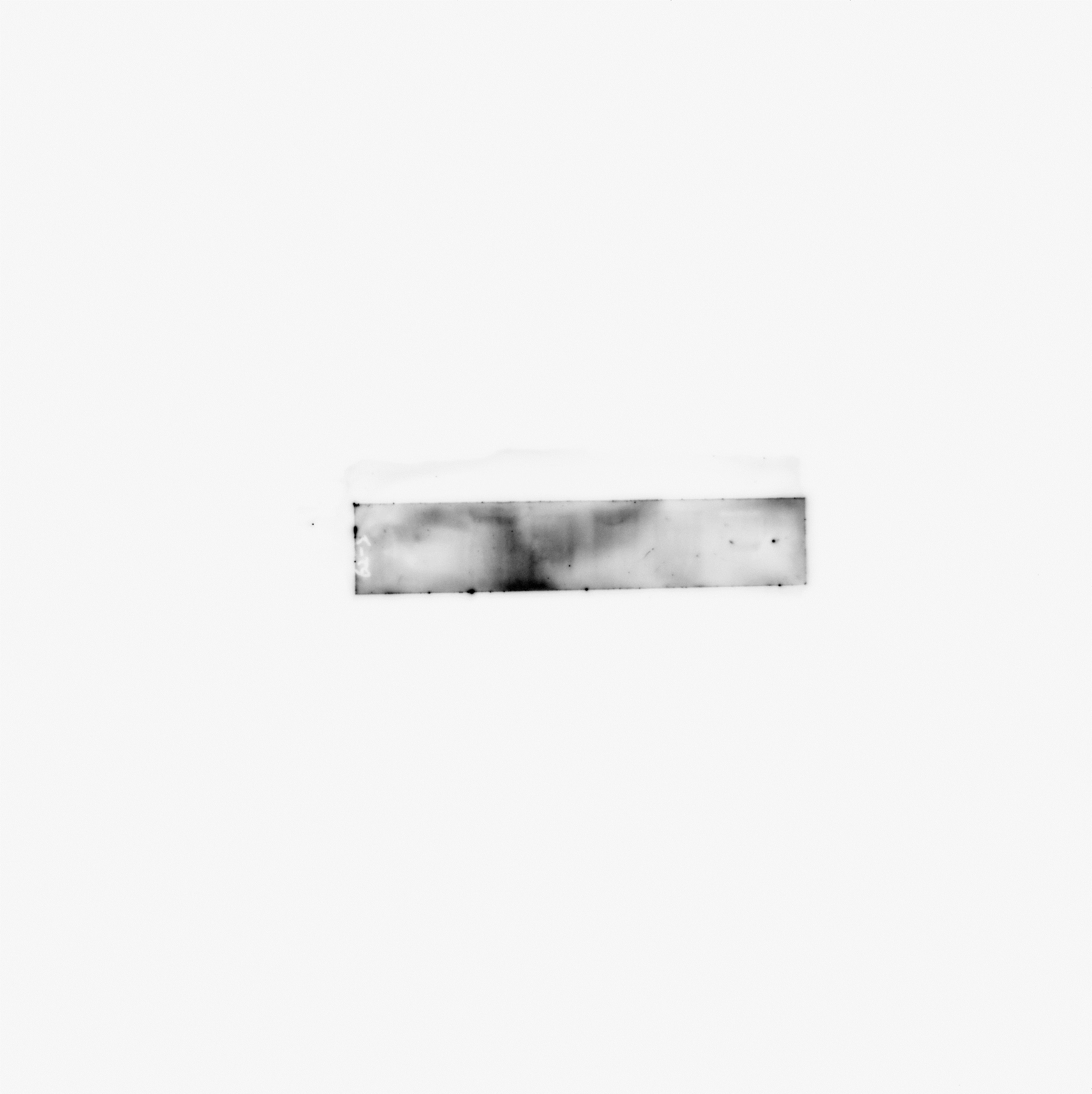

Supplement: Supplementary file 8 — Source data Fig. 2 [file 44321_2025_371_MOESM8_ESM.zip › Figure 2/Fig. 2h/Fig. 2h SQSTM1.tif]

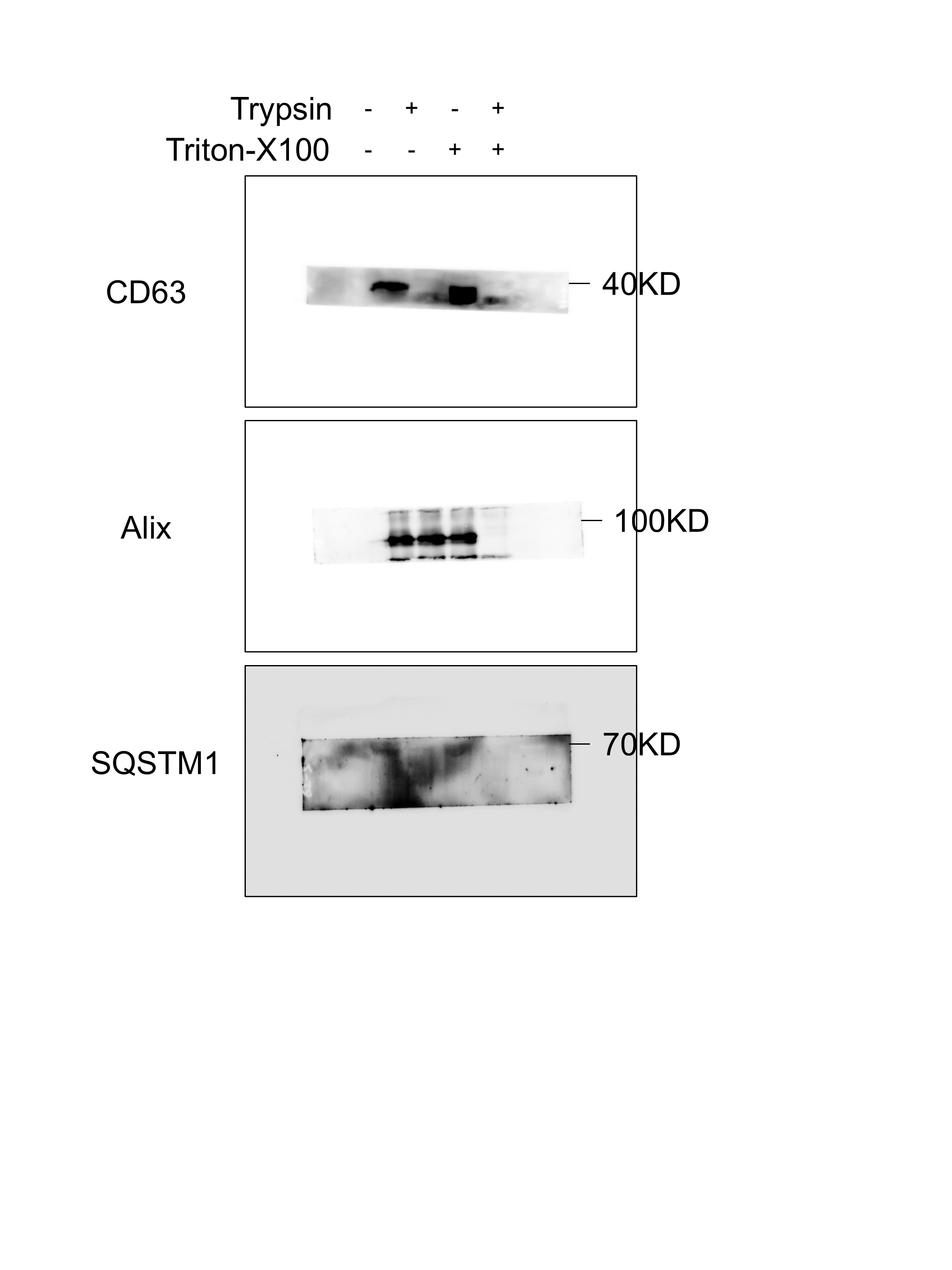

Supplement: Supplementary file 8 — Source data Fig. 2 [file 44321_2025_371_MOESM8_ESM.zip › Figure 2/Fig. 2h/Fig. 2h Summary plus label.tif]

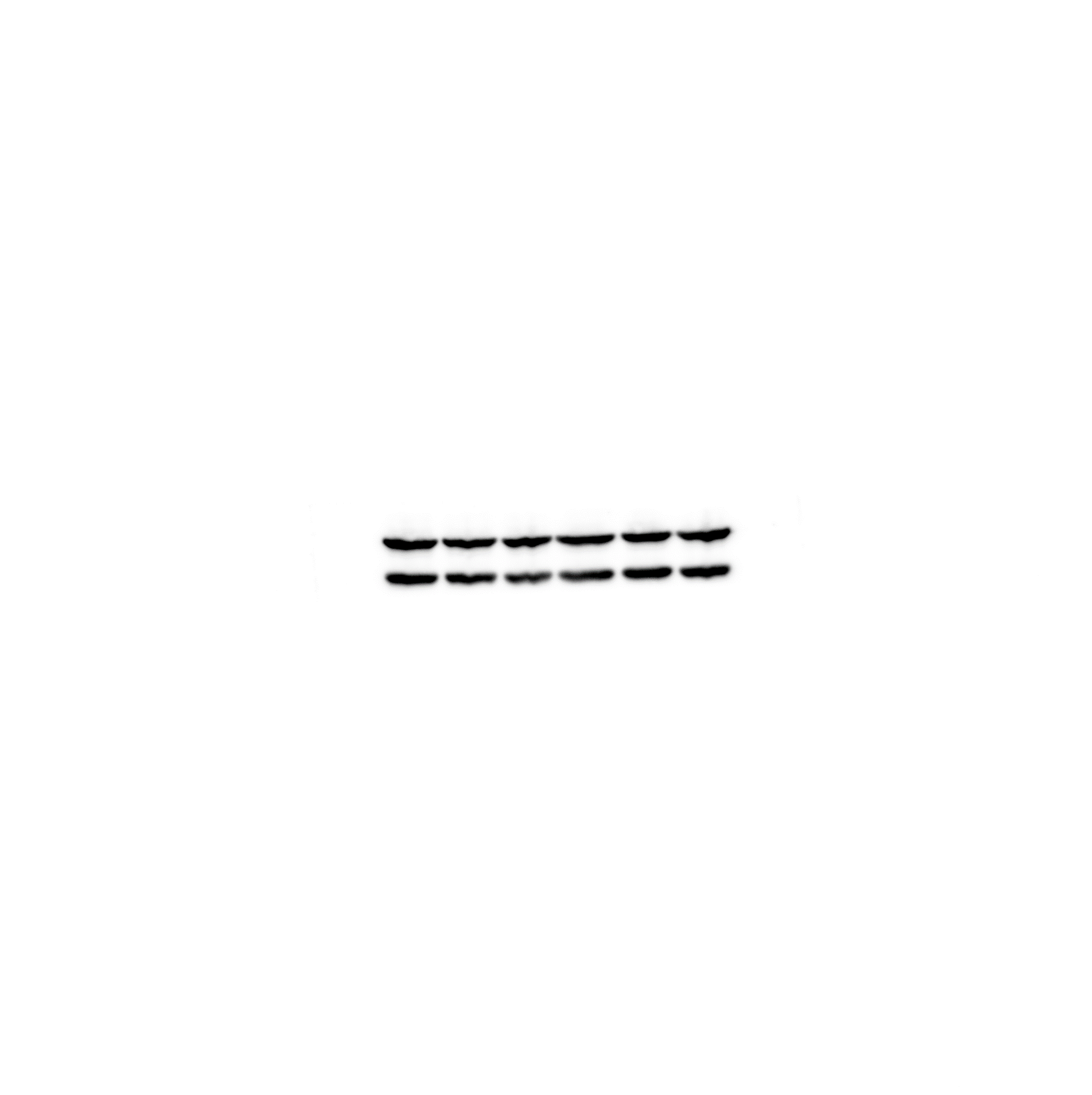

Supplement: Supplementary file 8 — Source data Fig. 2 [file 44321_2025_371_MOESM8_ESM.zip › Figure 2/Fig. 2i/Fig. 2i Deg-actin.tif]

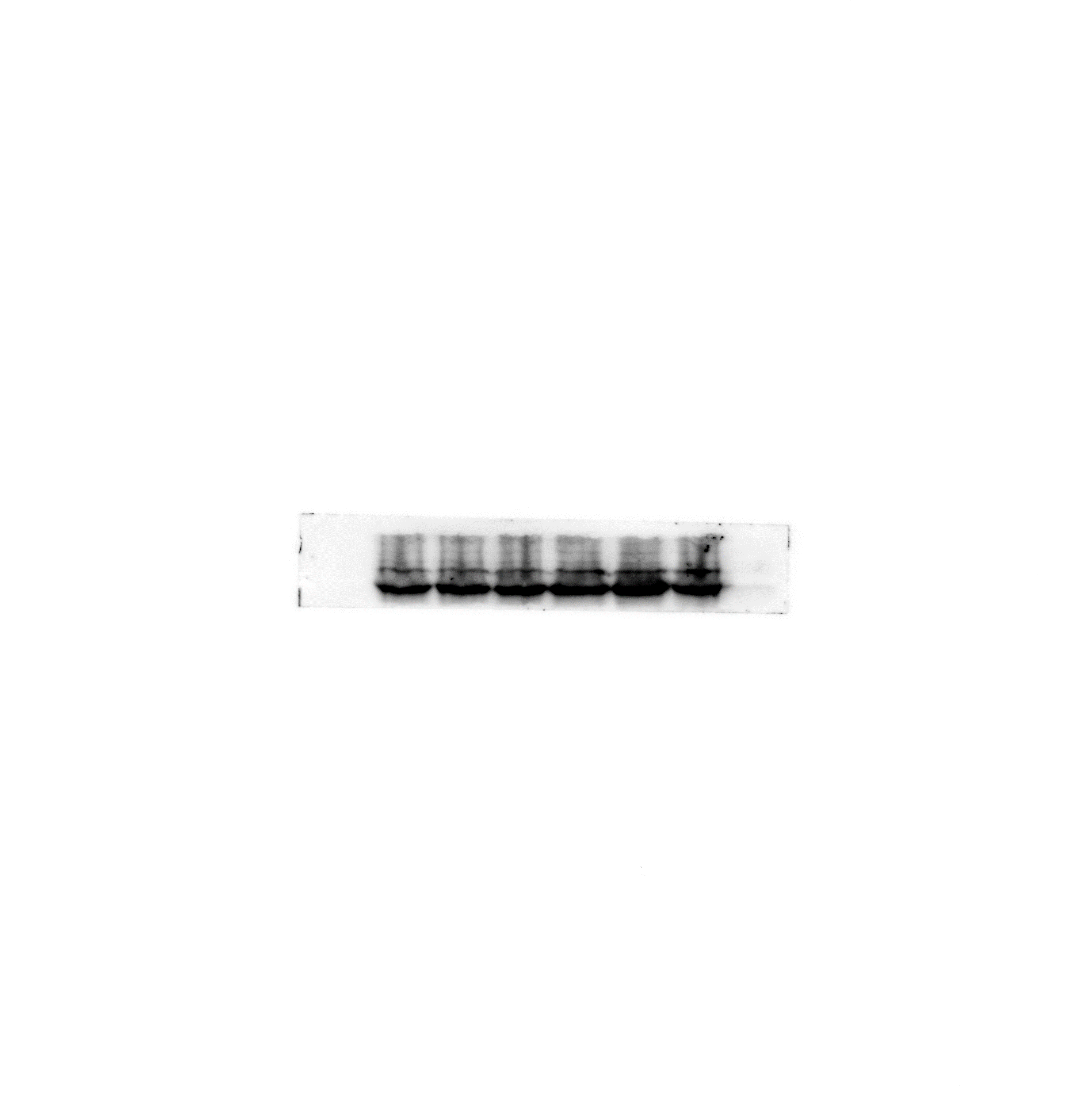

Supplement: Supplementary file 8 — Source data Fig. 2 [file 44321_2025_371_MOESM8_ESM.zip › Figure 2/Fig. 2i/Fig. 2i Deg-GFP.tif]

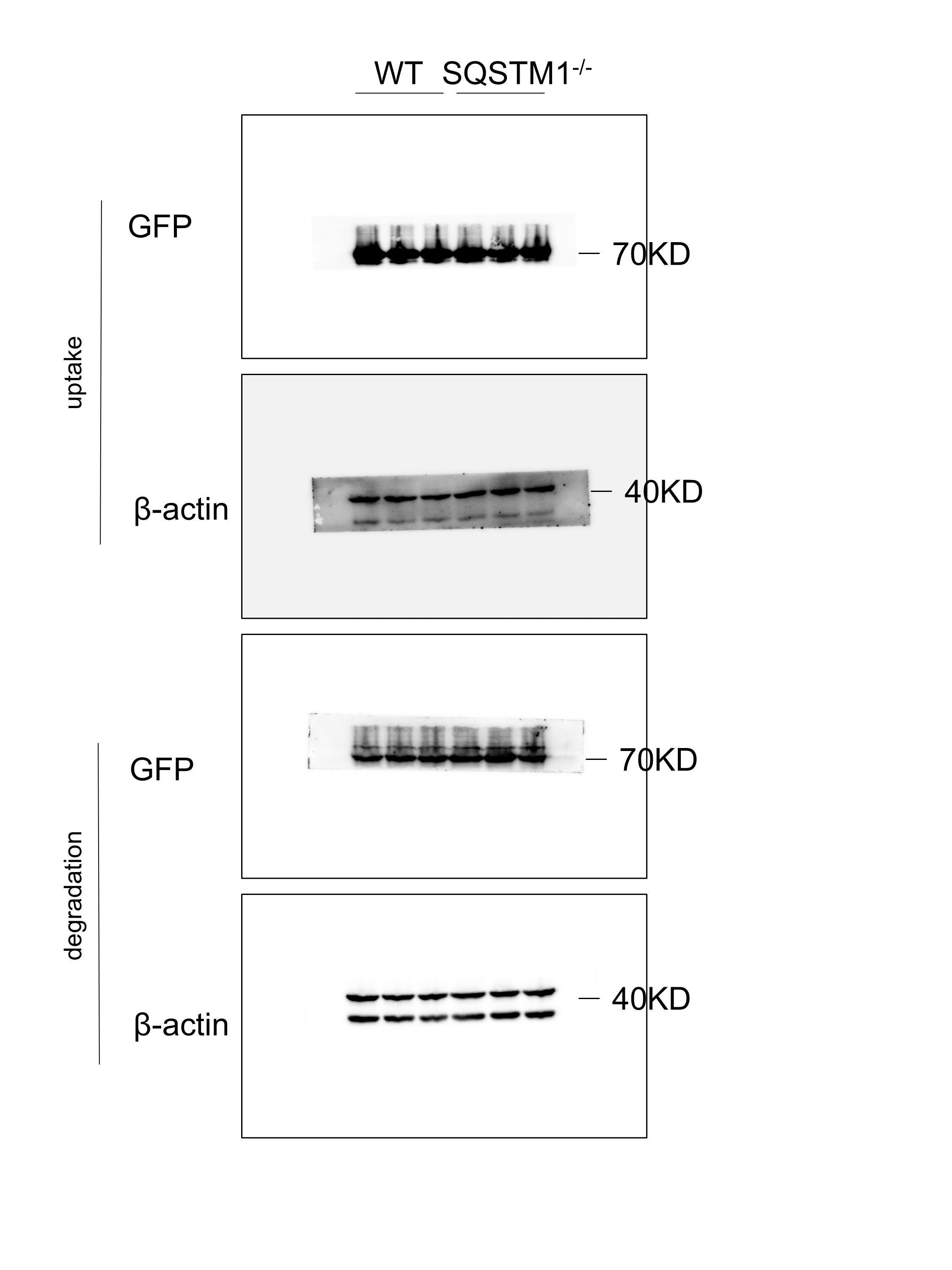

Supplement: Supplementary file 8 — Source data Fig. 2 [file 44321_2025_371_MOESM8_ESM.zip › Figure 2/Fig. 2i/Fig. 2i summary plus label.tif]

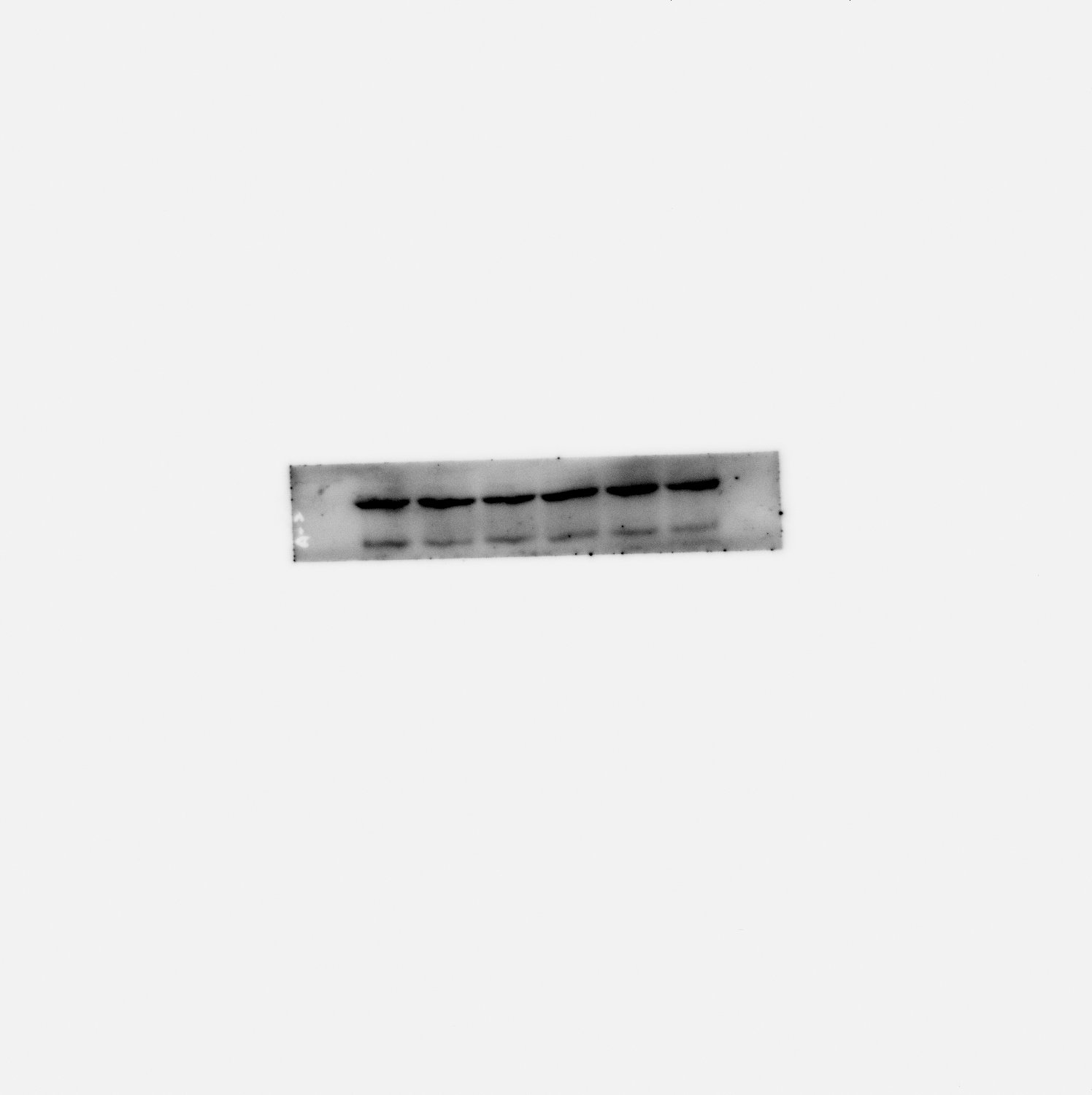

Supplement: Supplementary file 8 — Source data Fig. 2 [file 44321_2025_371_MOESM8_ESM.zip › Figure 2/Fig. 2i/Fig. 2i Uptake-actin.tif]

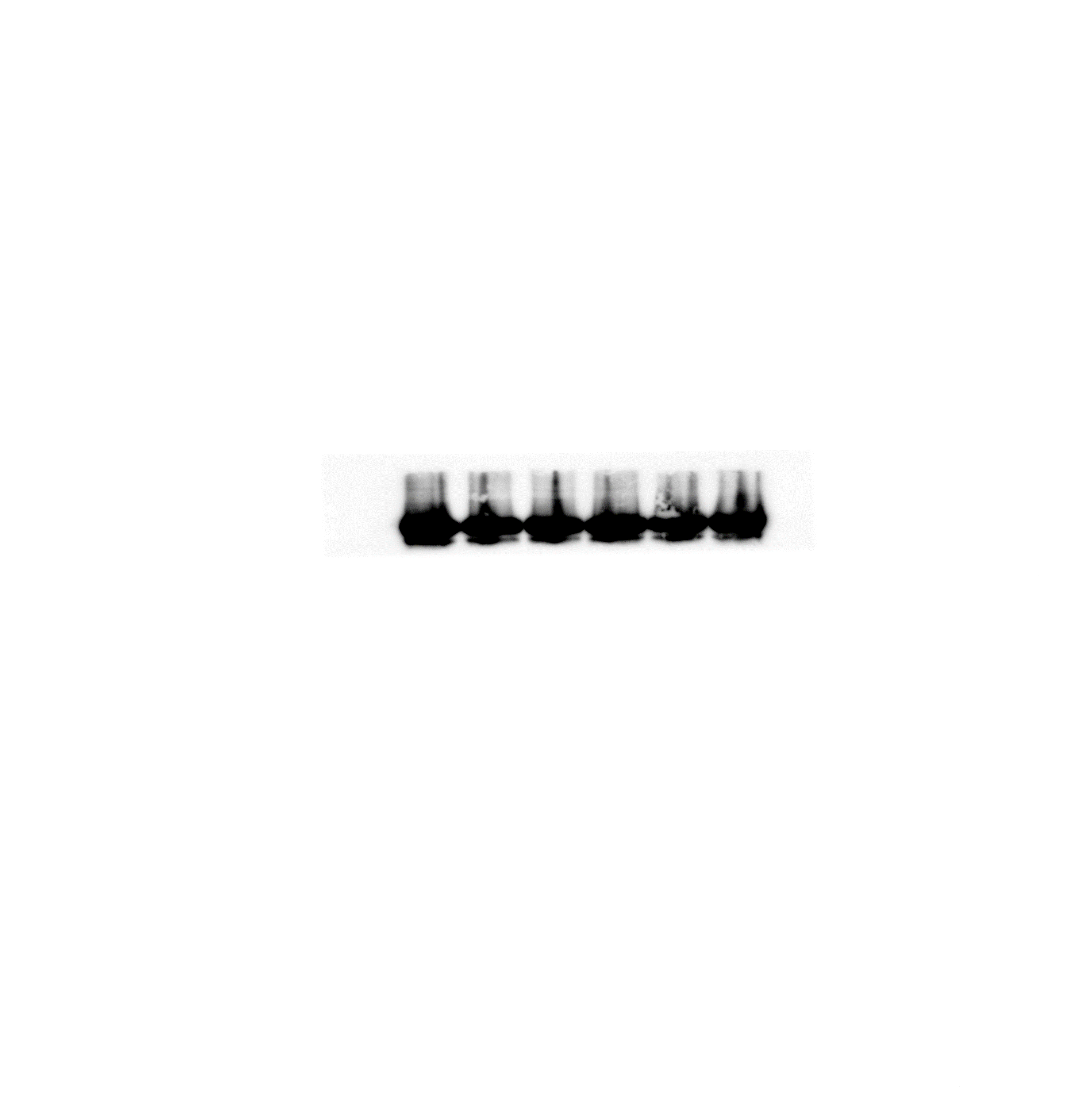

Supplement: Supplementary file 8 — Source data Fig. 2 [file 44321_2025_371_MOESM8_ESM.zip › Figure 2/Fig. 2i/Fig. 2i Uptake-GFP.tif]

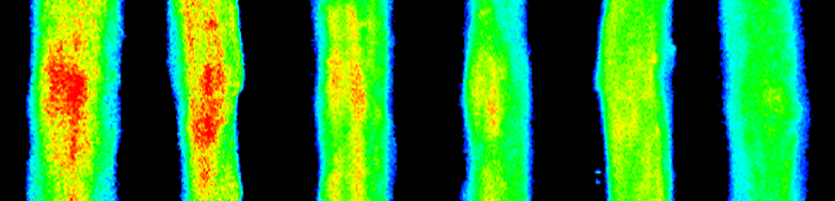

Supplement: Supplementary file 8 — Source data Fig. 2 [file 44321_2025_371_MOESM8_ESM.zip › Figure 2/Fig. 2k/Fig. 2k Con-EV 0-20d.tif]

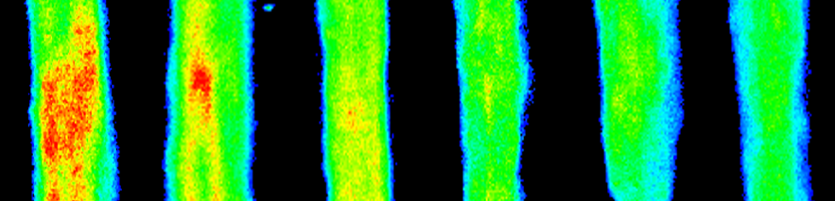

Supplement: Supplementary file 8 — Source data Fig. 2 [file 44321_2025_371_MOESM8_ESM.zip › Figure 2/Fig. 2k/Fig. 2k SQSTM1-EV 0-20d.tif]

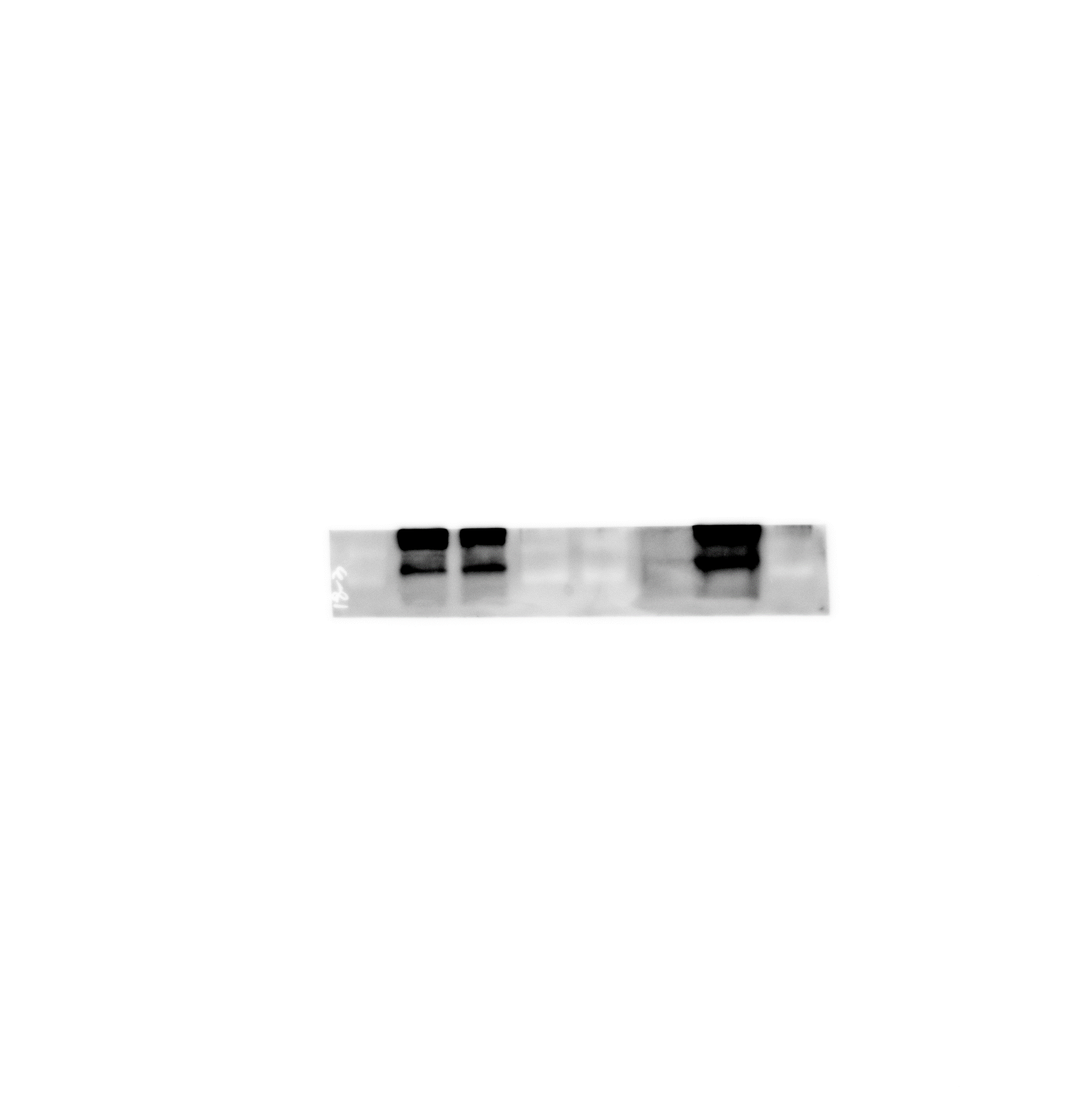

Supplement: Supplementary file 9 — Source data Fig. 3 [file 44321_2025_371_MOESM9_ESM.zip › Figure 3/Fig. 3d/Fig. 3d-Left-Flag.tif]

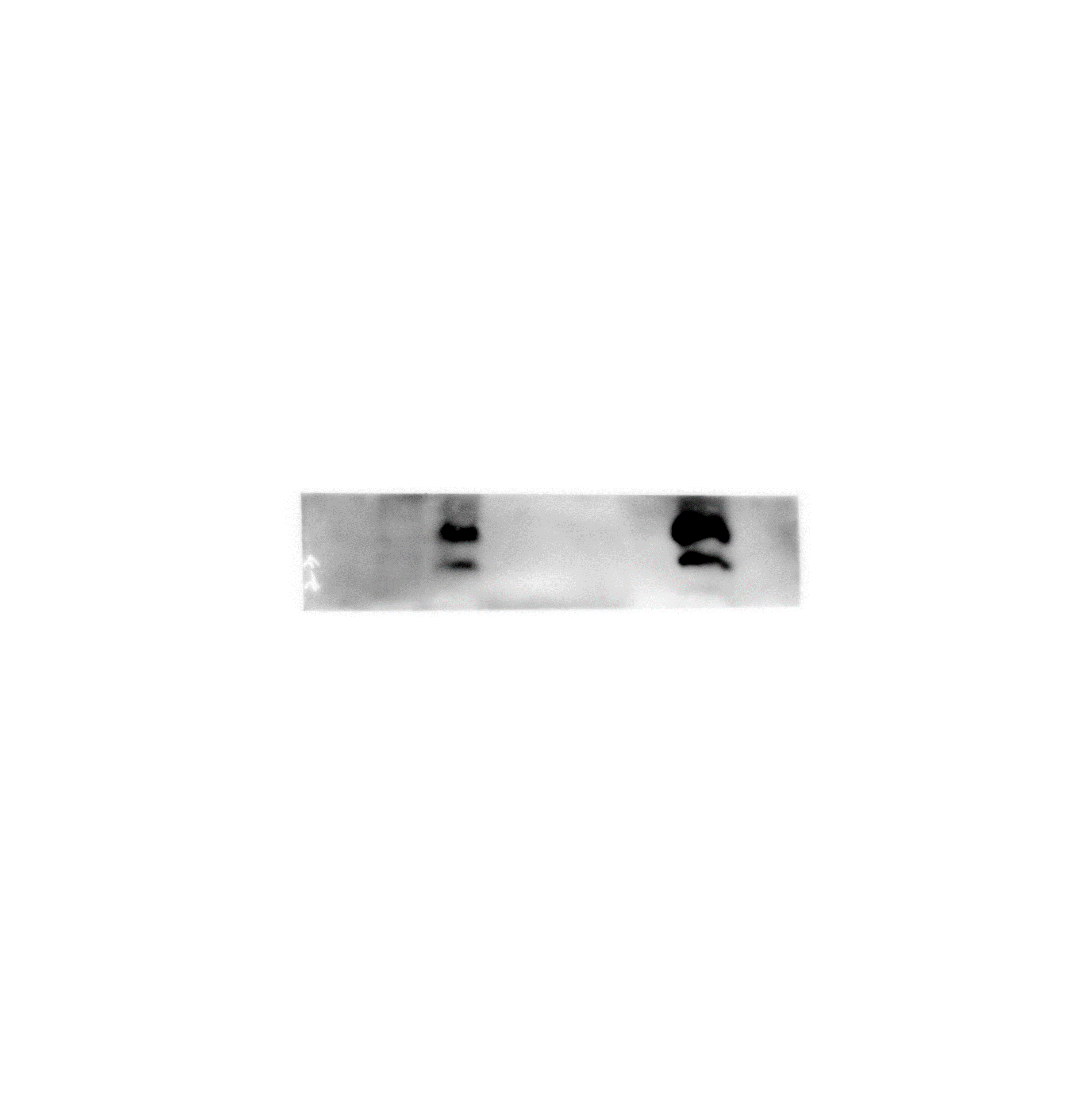

Supplement: Supplementary file 9 — Source data Fig. 3 [file 44321_2025_371_MOESM9_ESM.zip › Figure 3/Fig. 3d/Fig. 3d-Left-His.tif]

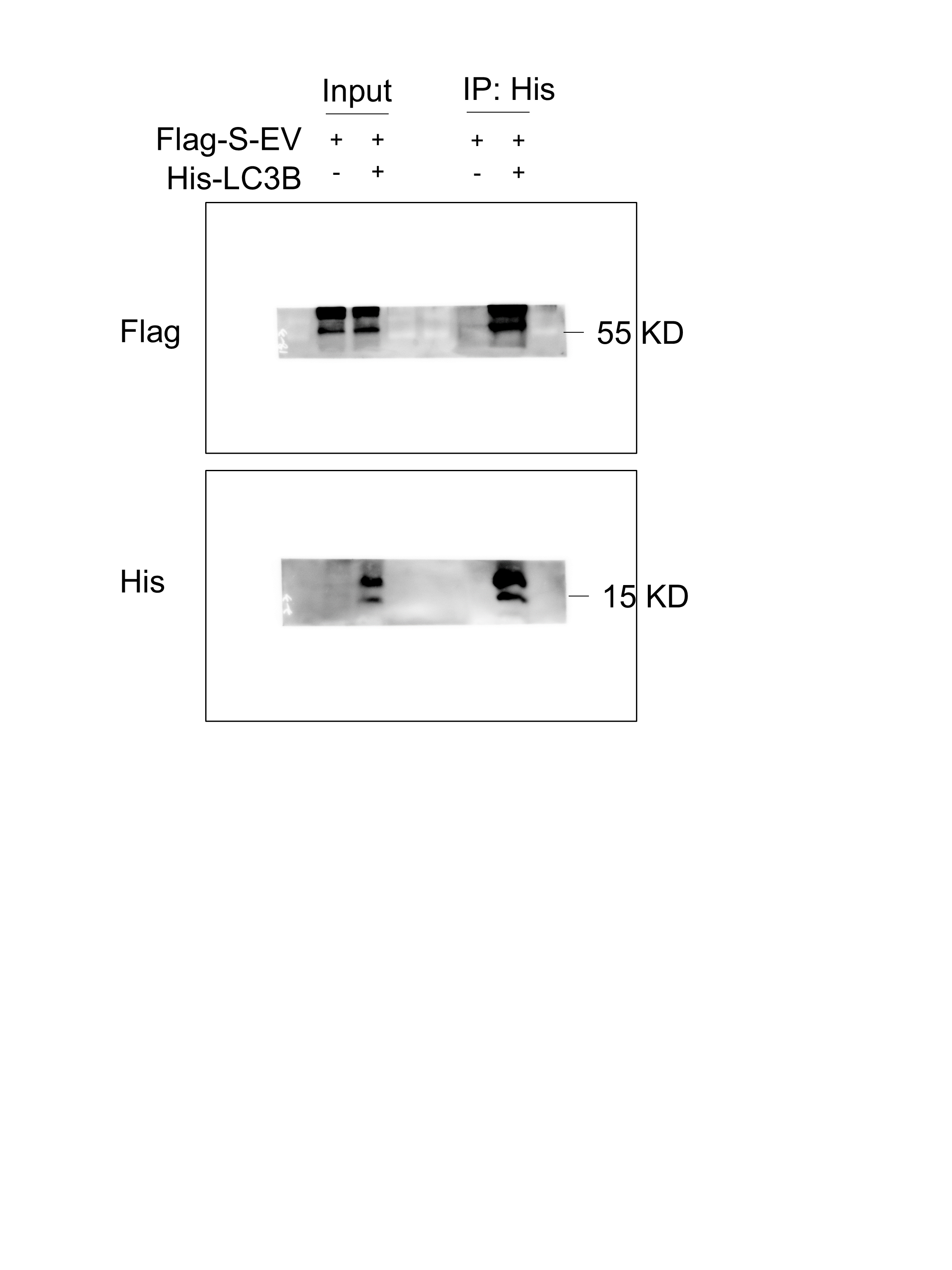

Supplement: Supplementary file 9 — Source data Fig. 3 [file 44321_2025_371_MOESM9_ESM.zip › Figure 3/Fig. 3d/Fig. 3d-Left-Summary plus label.tif]

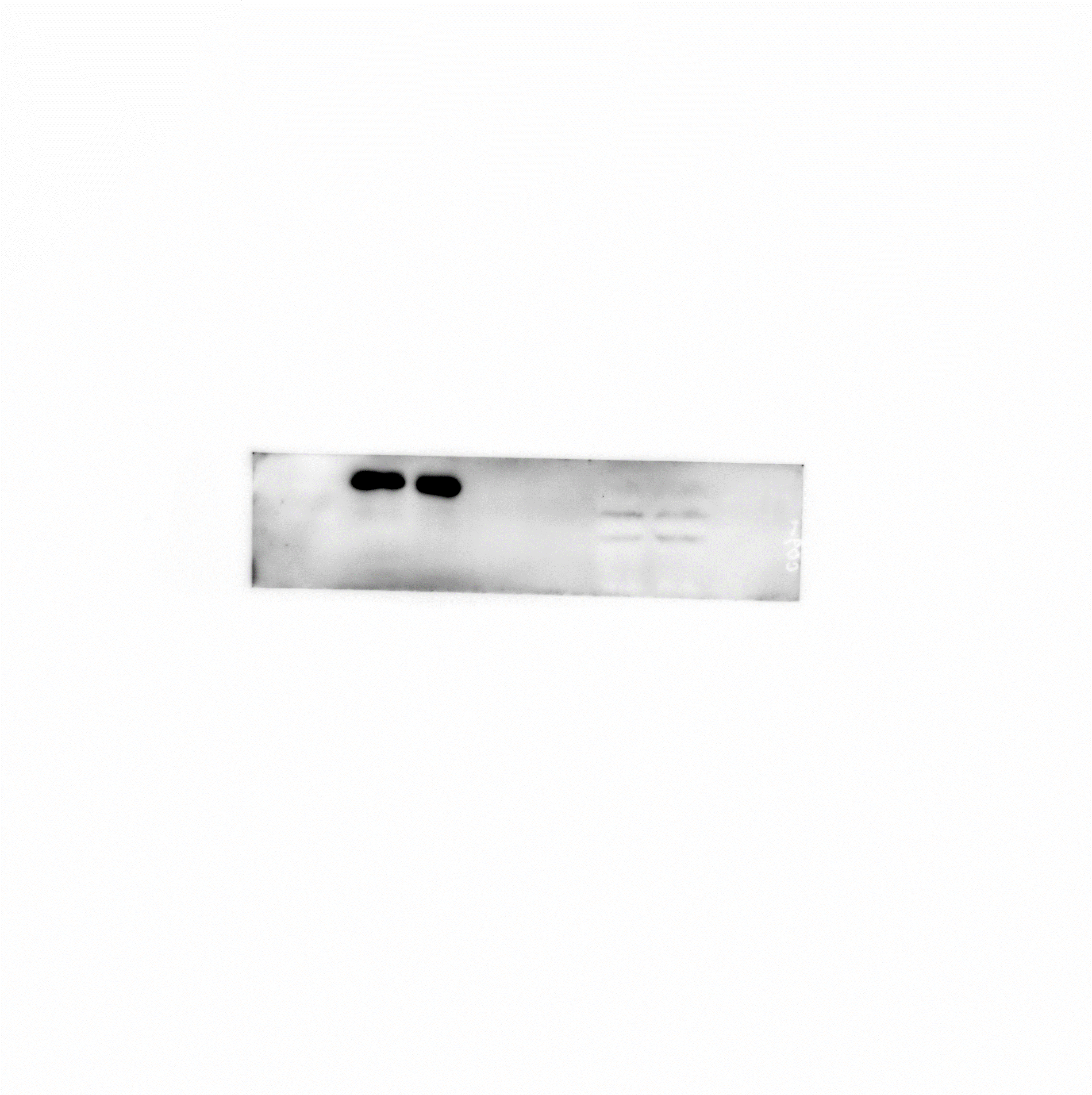

Supplement: Supplementary file 9 — Source data Fig. 3 [file 44321_2025_371_MOESM9_ESM.zip › Figure 3/Fig. 3d/Fig. 3d-Right-Flag.tif]

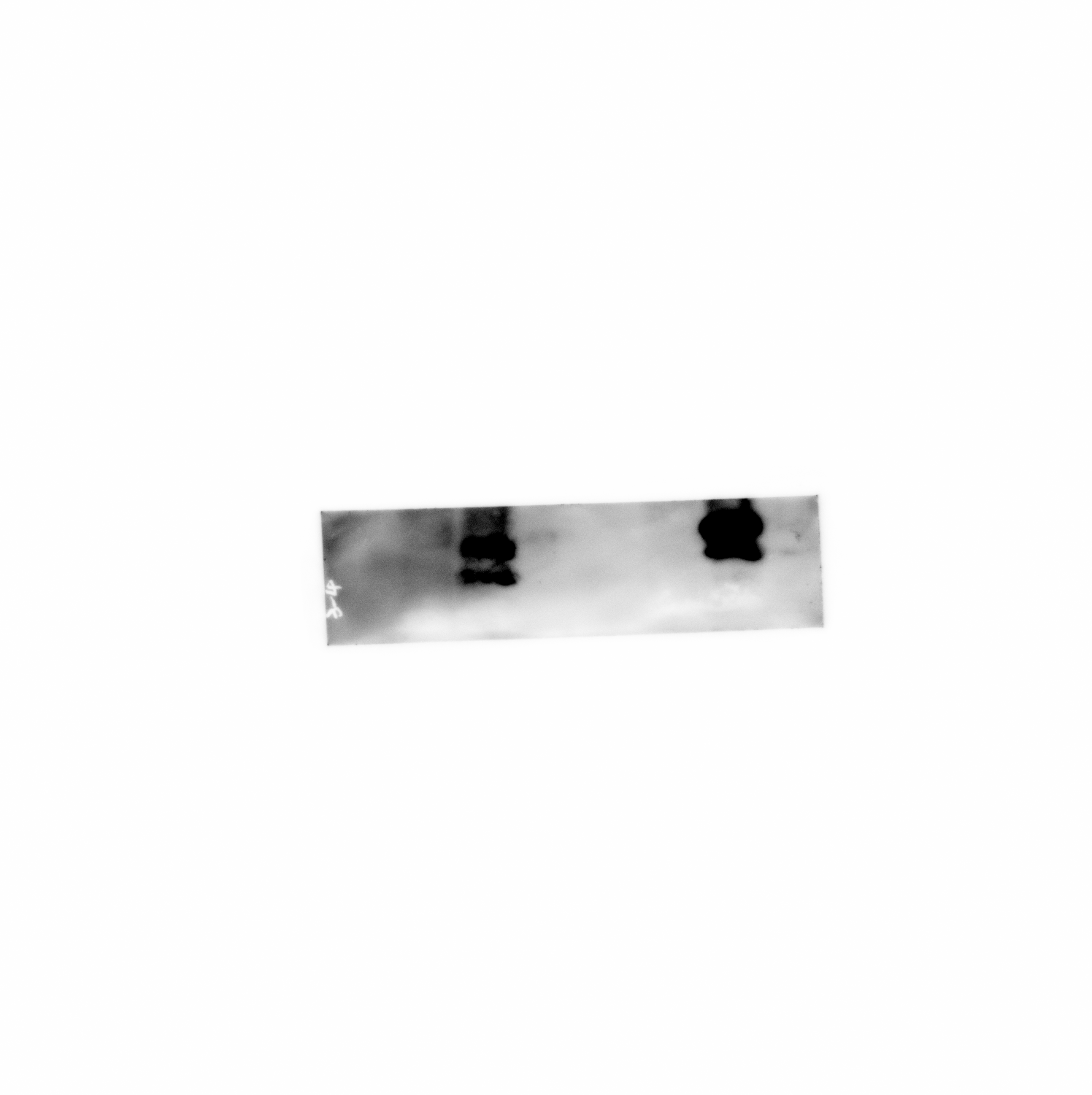

Supplement: Supplementary file 9 — Source data Fig. 3 [file 44321_2025_371_MOESM9_ESM.zip › Figure 3/Fig. 3d/Fig. 3d-Right-His.tif]

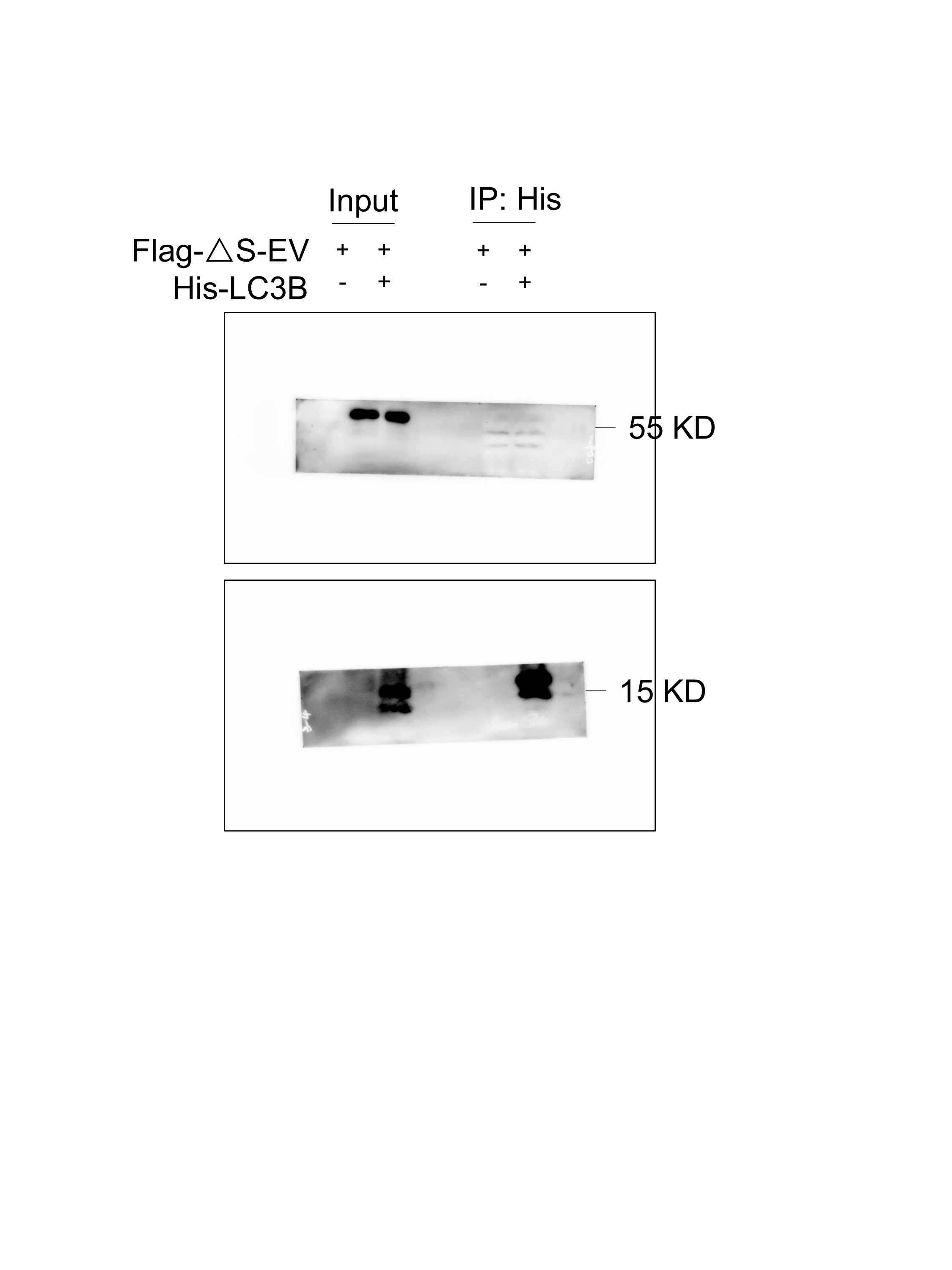

Supplement: Supplementary file 9 — Source data Fig. 3 [file 44321_2025_371_MOESM9_ESM.zip › Figure 3/Fig. 3d/Fig. 3d-Right-Summary plus label.tif]

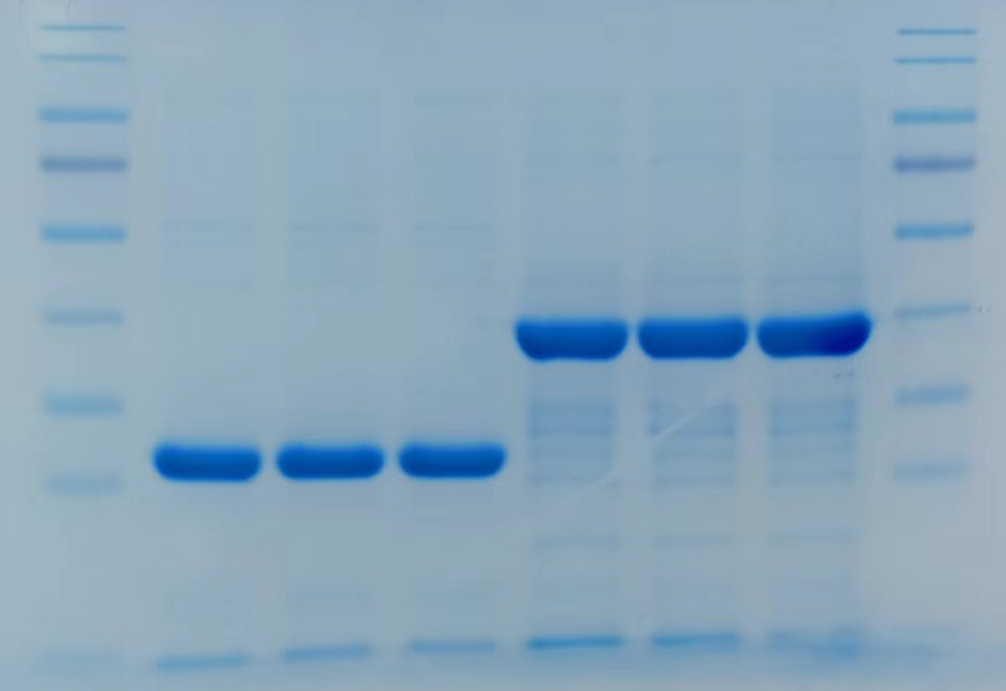

Supplement: Supplementary file 9 — Source data Fig. 3 [file 44321_2025_371_MOESM9_ESM.zip › Figure 3/Fig. 3e/Fig. 3e Input Cooma.tif]

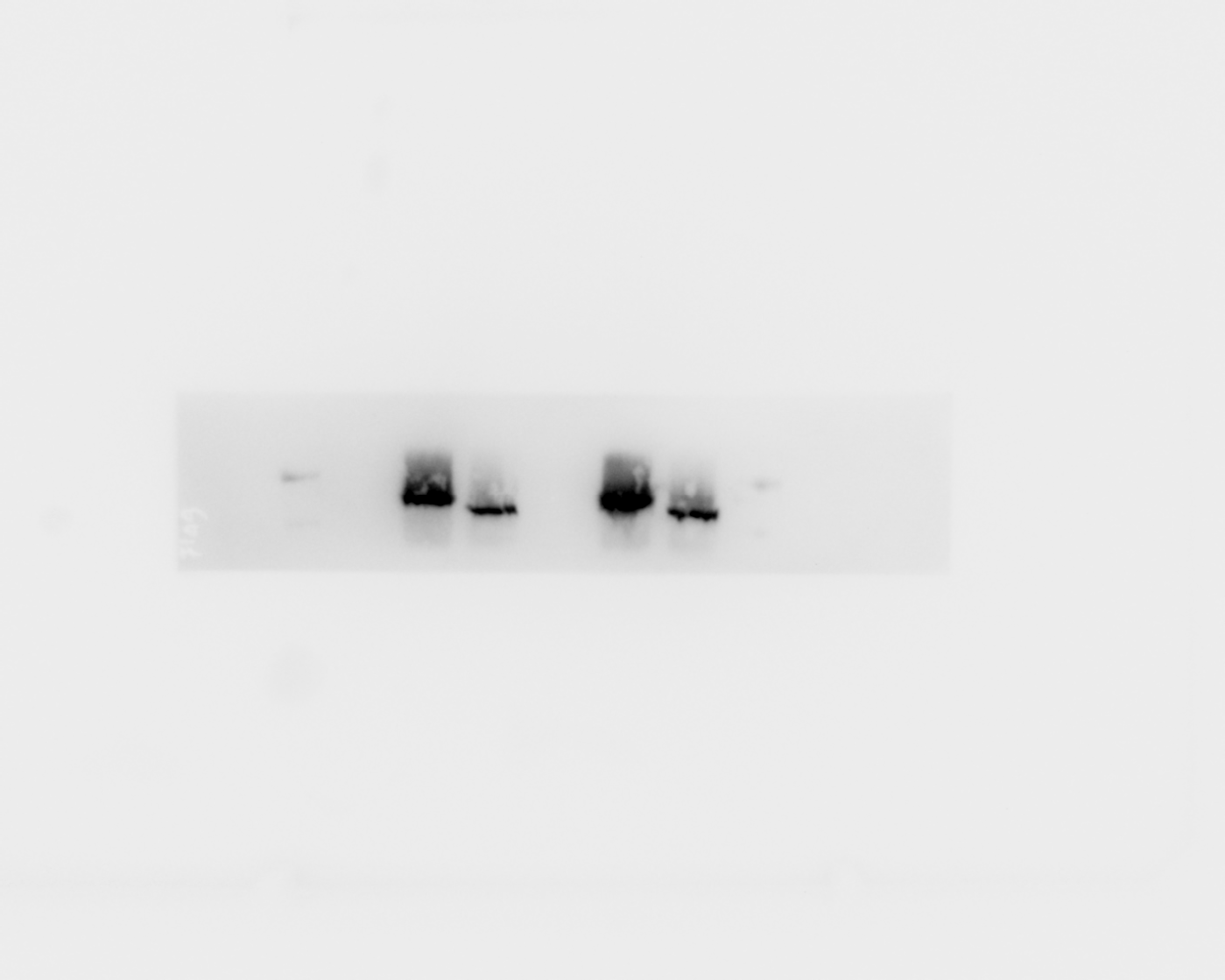

Supplement: Supplementary file 9 — Source data Fig. 3 [file 44321_2025_371_MOESM9_ESM.zip › Figure 3/Fig. 3e/Fig. 3e Input Flag.tif]

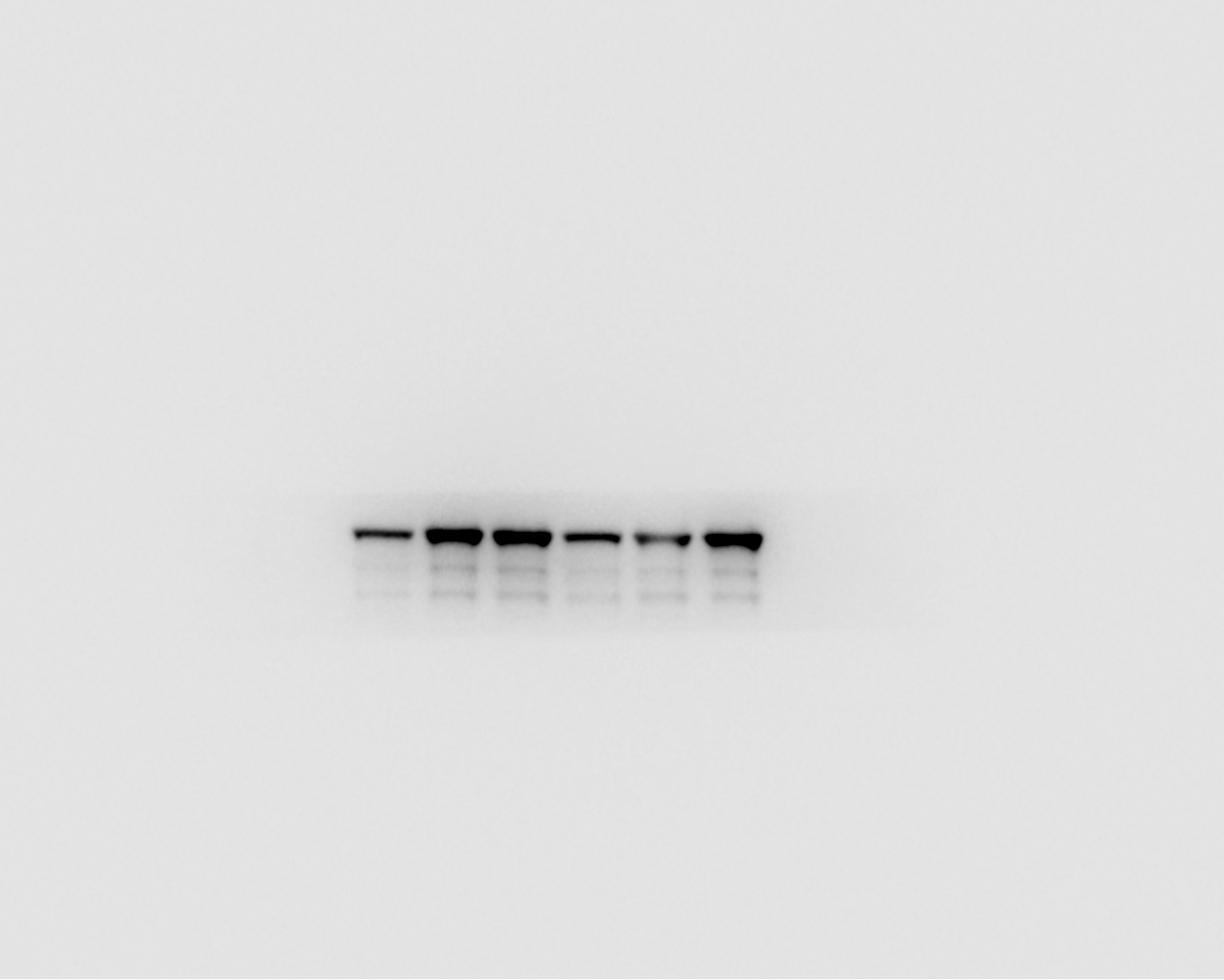

Supplement: Supplementary file 9 — Source data Fig. 3 [file 44321_2025_371_MOESM9_ESM.zip › Figure 3/Fig. 3e/Fig. 3e Input TSG101.tif]

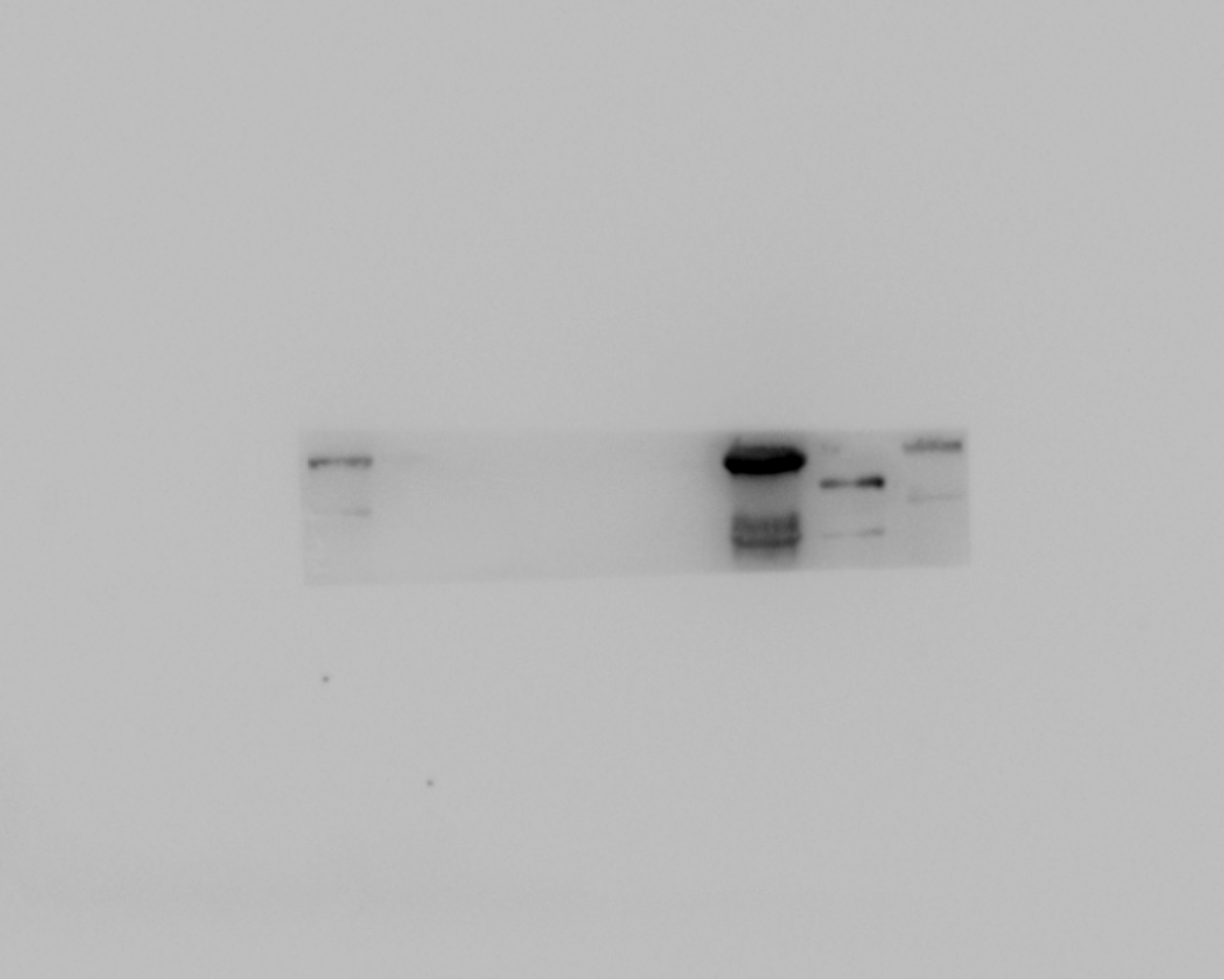

Supplement: Supplementary file 9 — Source data Fig. 3 [file 44321_2025_371_MOESM9_ESM.zip › Figure 3/Fig. 3e/Fig. 3e pull down Flag.tif]

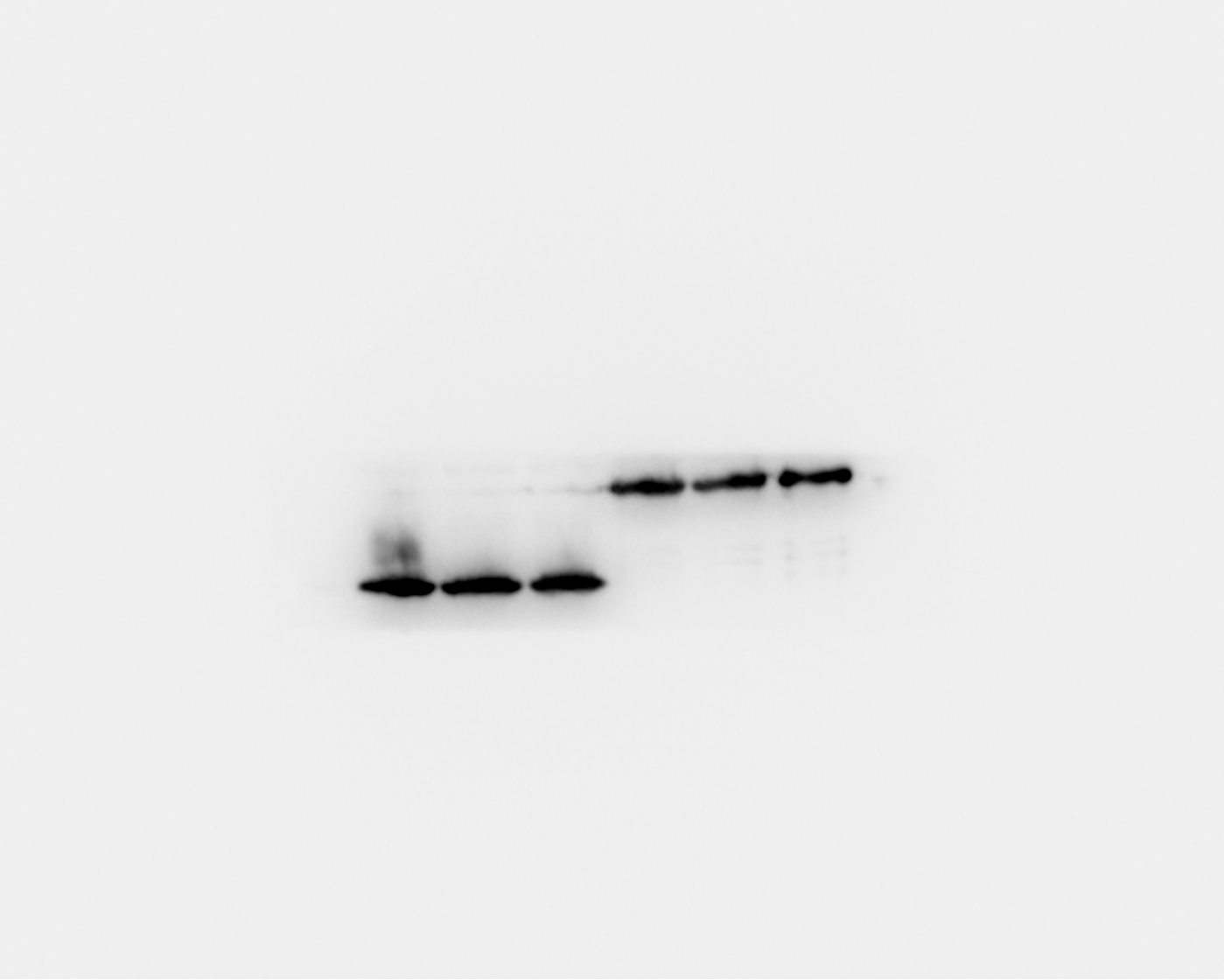

Supplement: Supplementary file 9 — Source data Fig. 3 [file 44321_2025_371_MOESM9_ESM.zip › Figure 3/Fig. 3e/Fig. 3e pull down GST.tif]

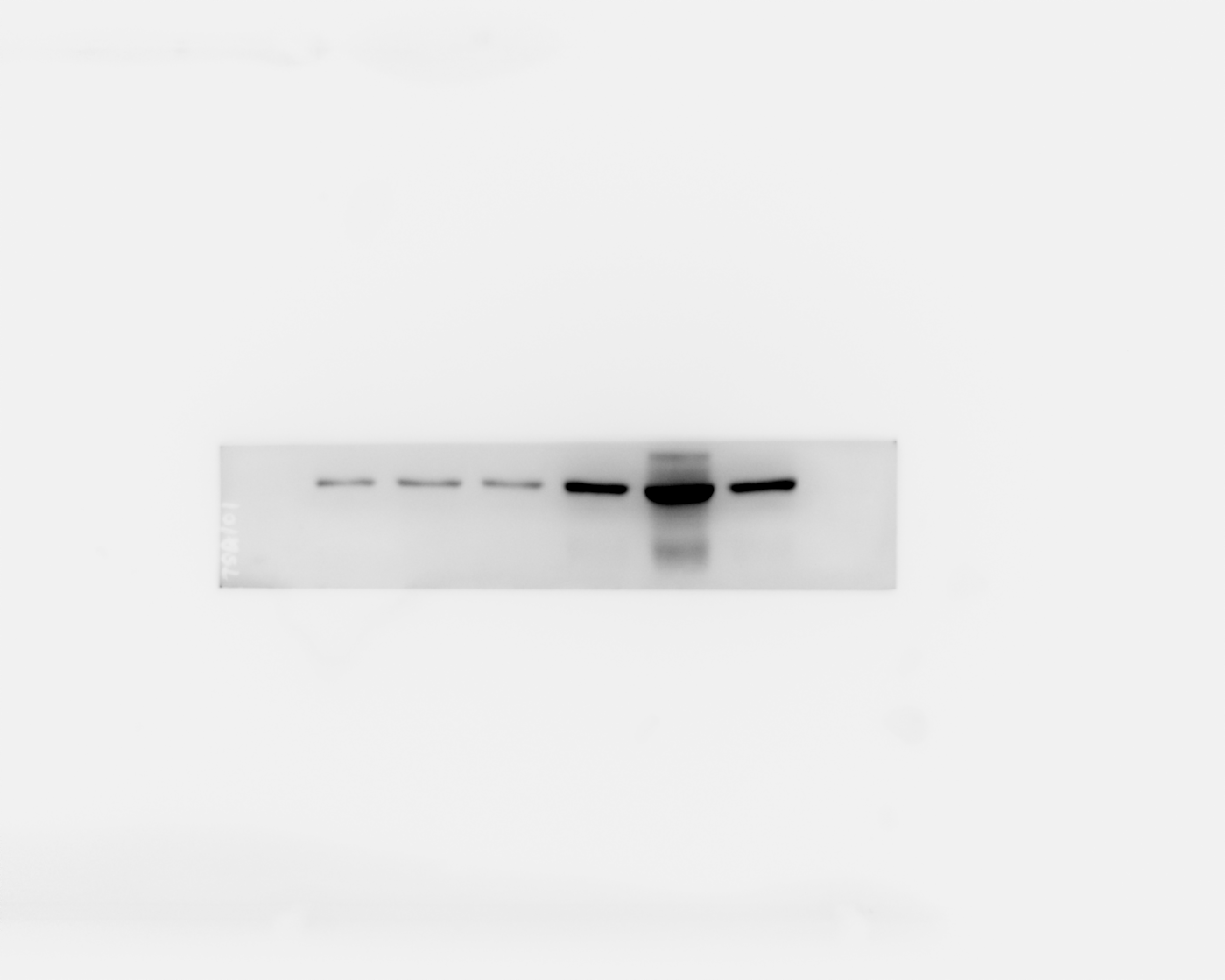

Supplement: Supplementary file 9 — Source data Fig. 3 [file 44321_2025_371_MOESM9_ESM.zip › Figure 3/Fig. 3e/Fig. 3e pull down TSG101.tif]

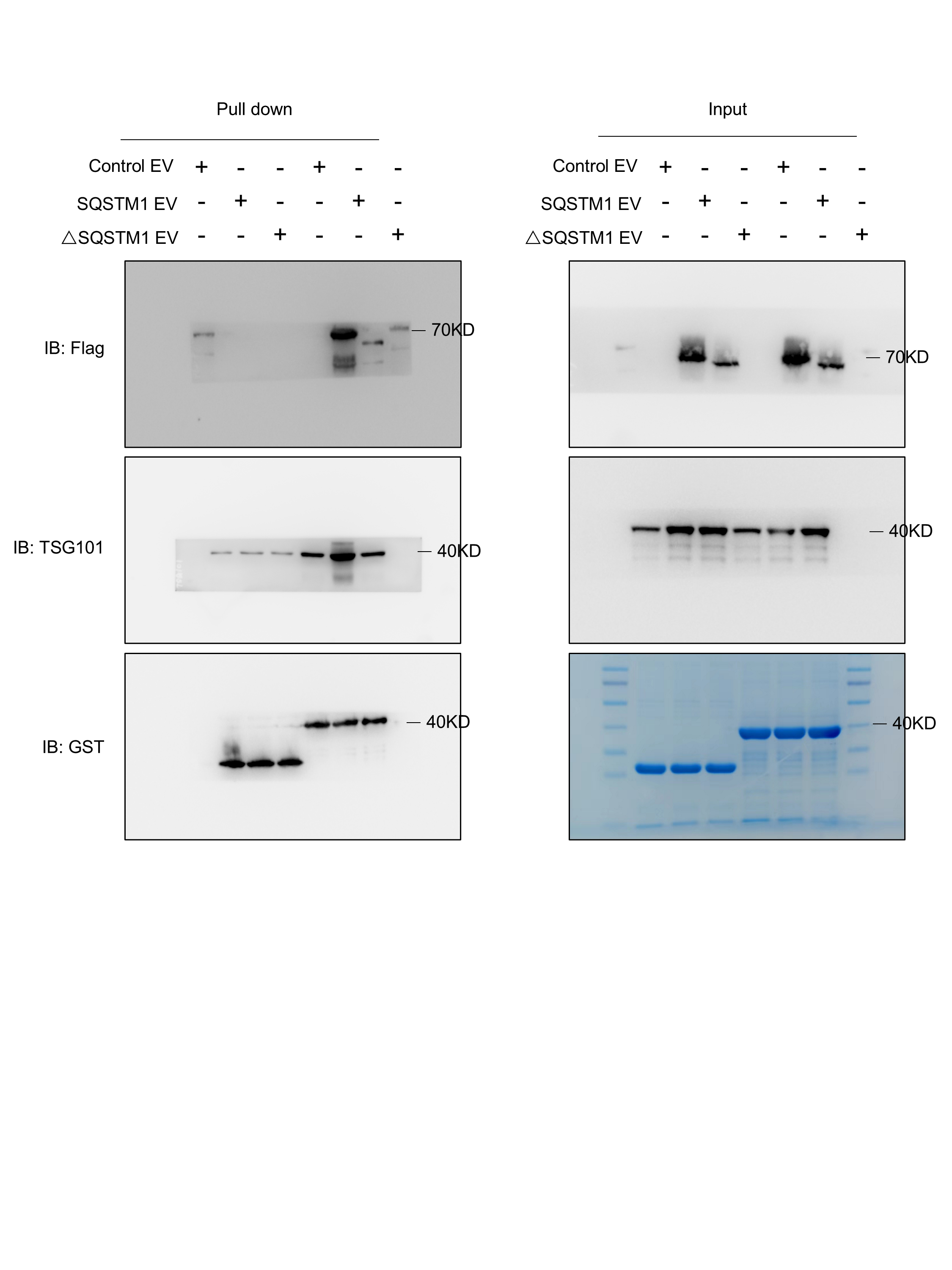

Supplement: Supplementary file 9 — Source data Fig. 3 [file 44321_2025_371_MOESM9_ESM.zip › Figure 3/Fig. 3e/Fig. 3e Summary plus Label.tif]

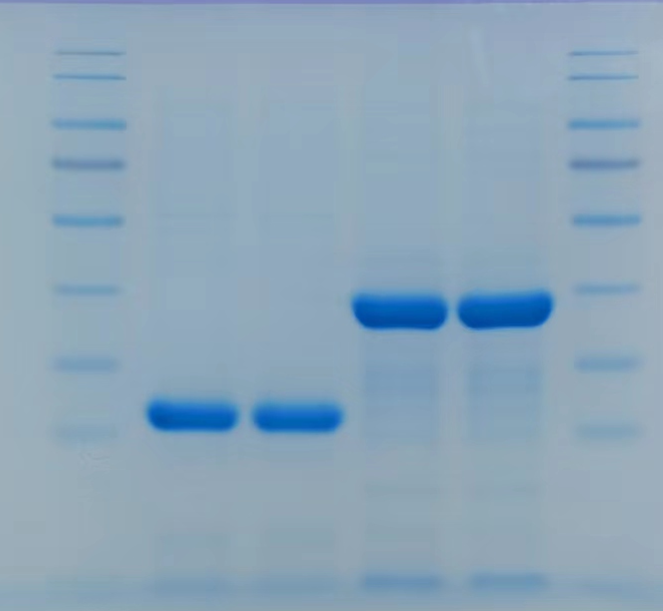

Supplement: Supplementary file 9 — Source data Fig. 3 [file 44321_2025_371_MOESM9_ESM.zip › Figure 3/Fig. 3f/Fig. 3f Input Cooma.tif]

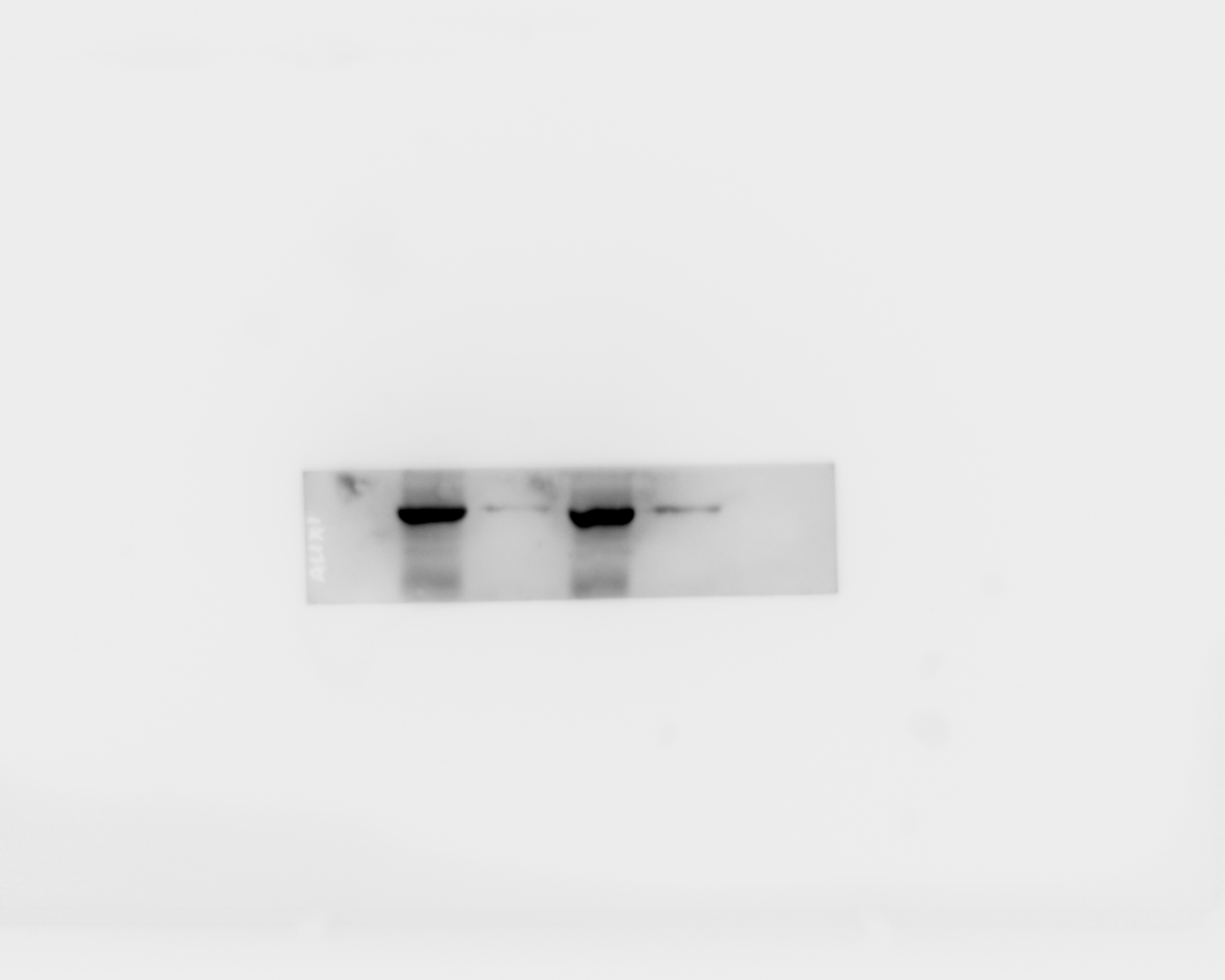

Supplement: Supplementary file 9 — Source data Fig. 3 [file 44321_2025_371_MOESM9_ESM.zip › Figure 3/Fig. 3f/Fig. 3f Input SQSTM1.tif]

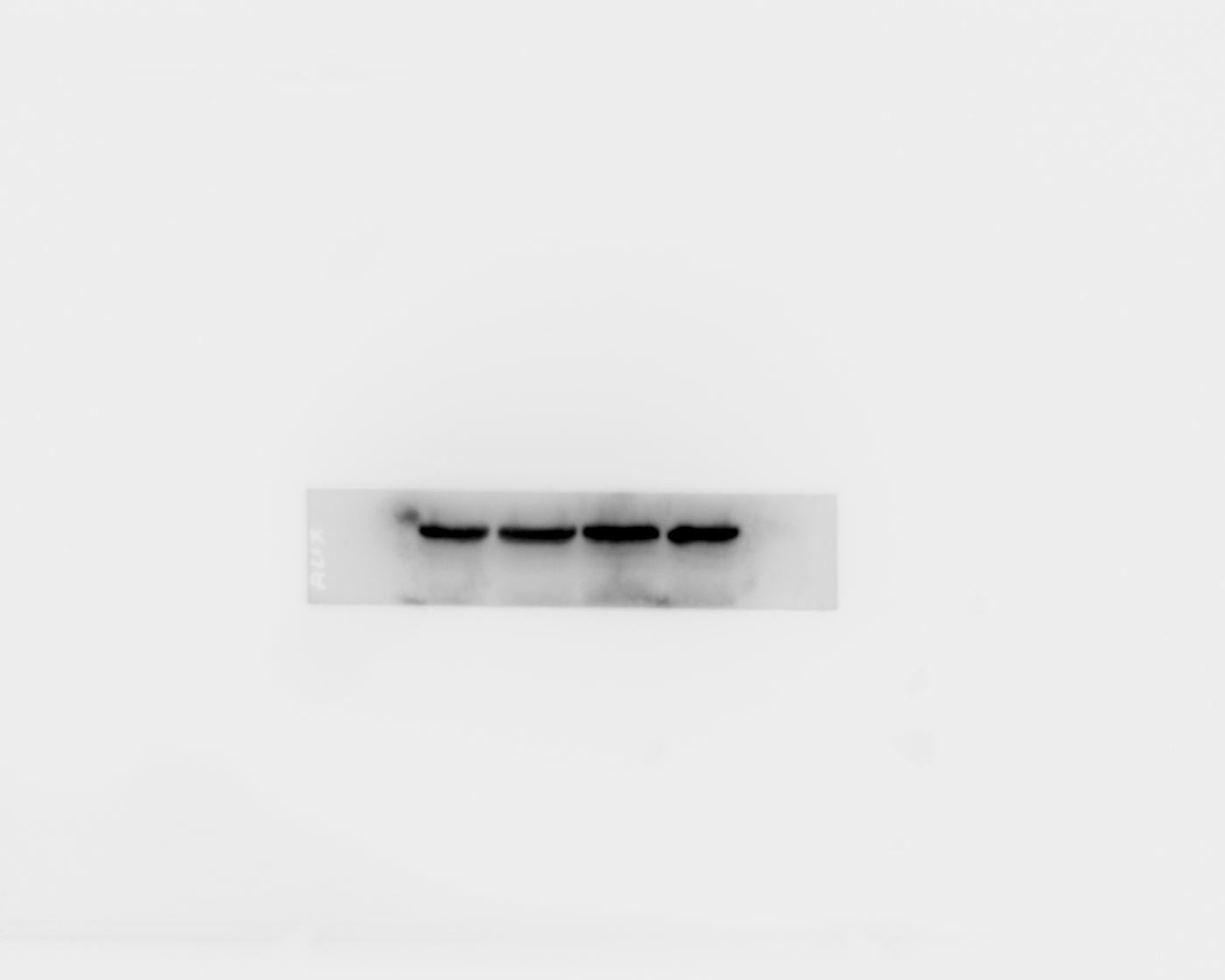

Supplement: Supplementary file 9 — Source data Fig. 3 [file 44321_2025_371_MOESM9_ESM.zip › Figure 3/Fig. 3f/Fig. 3f Input TSG101.tif]

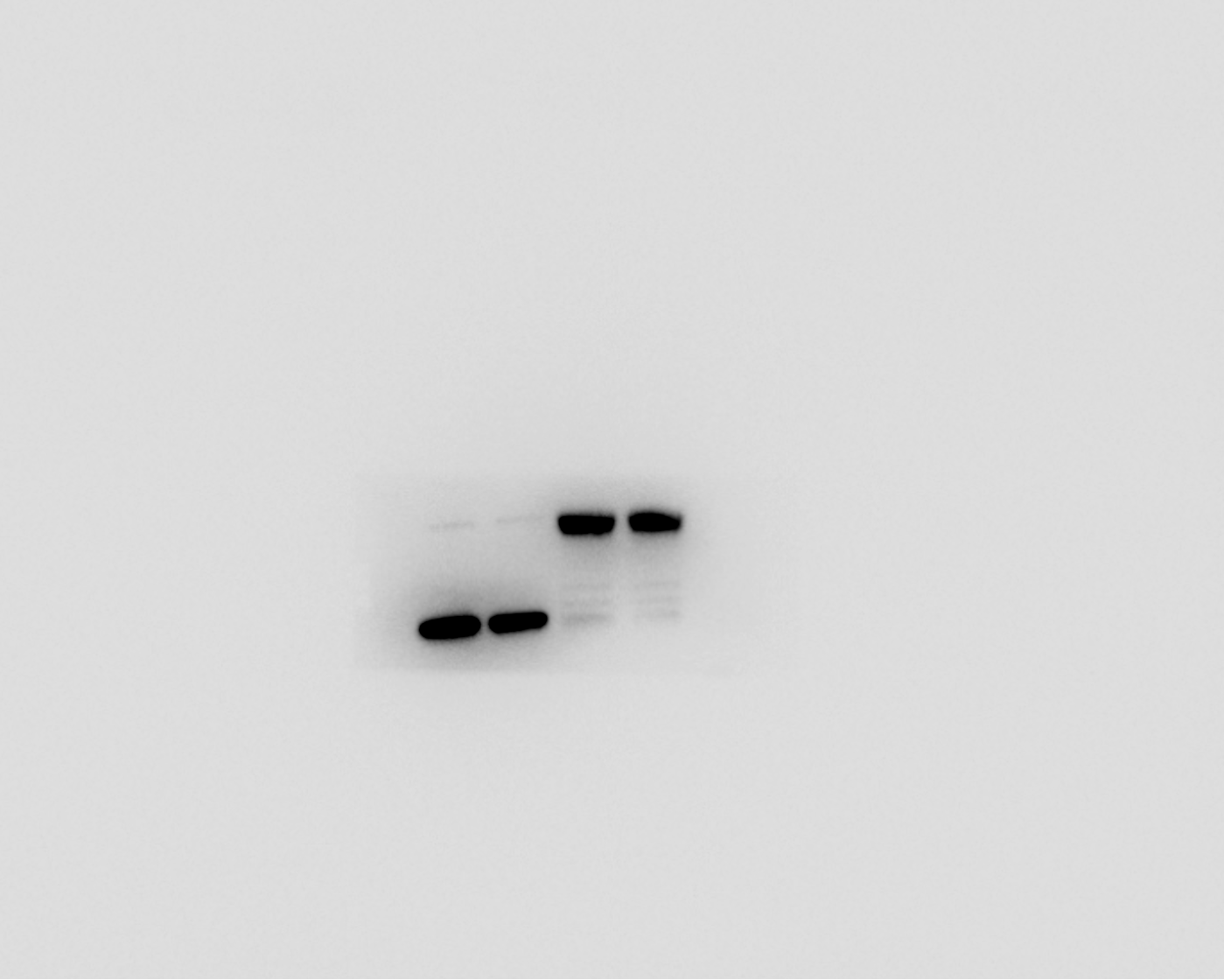

Supplement: Supplementary file 9 — Source data Fig. 3 [file 44321_2025_371_MOESM9_ESM.zip › Figure 3/Fig. 3f/Fig. 3f pull down GST.tif]

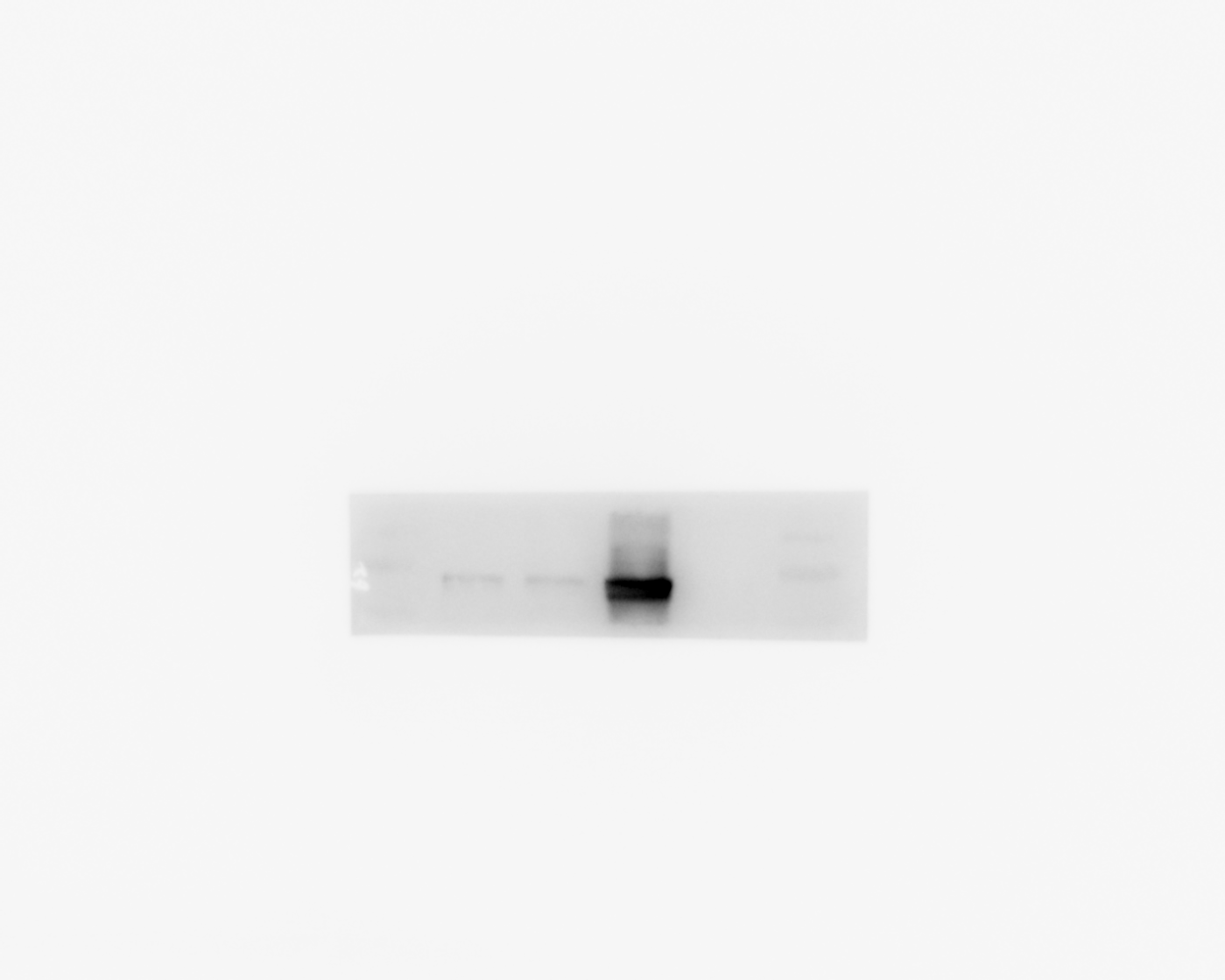

Supplement: Supplementary file 9 — Source data Fig. 3 [file 44321_2025_371_MOESM9_ESM.zip › Figure 3/Fig. 3f/Fig. 3f pull down SQSTM1.tif]

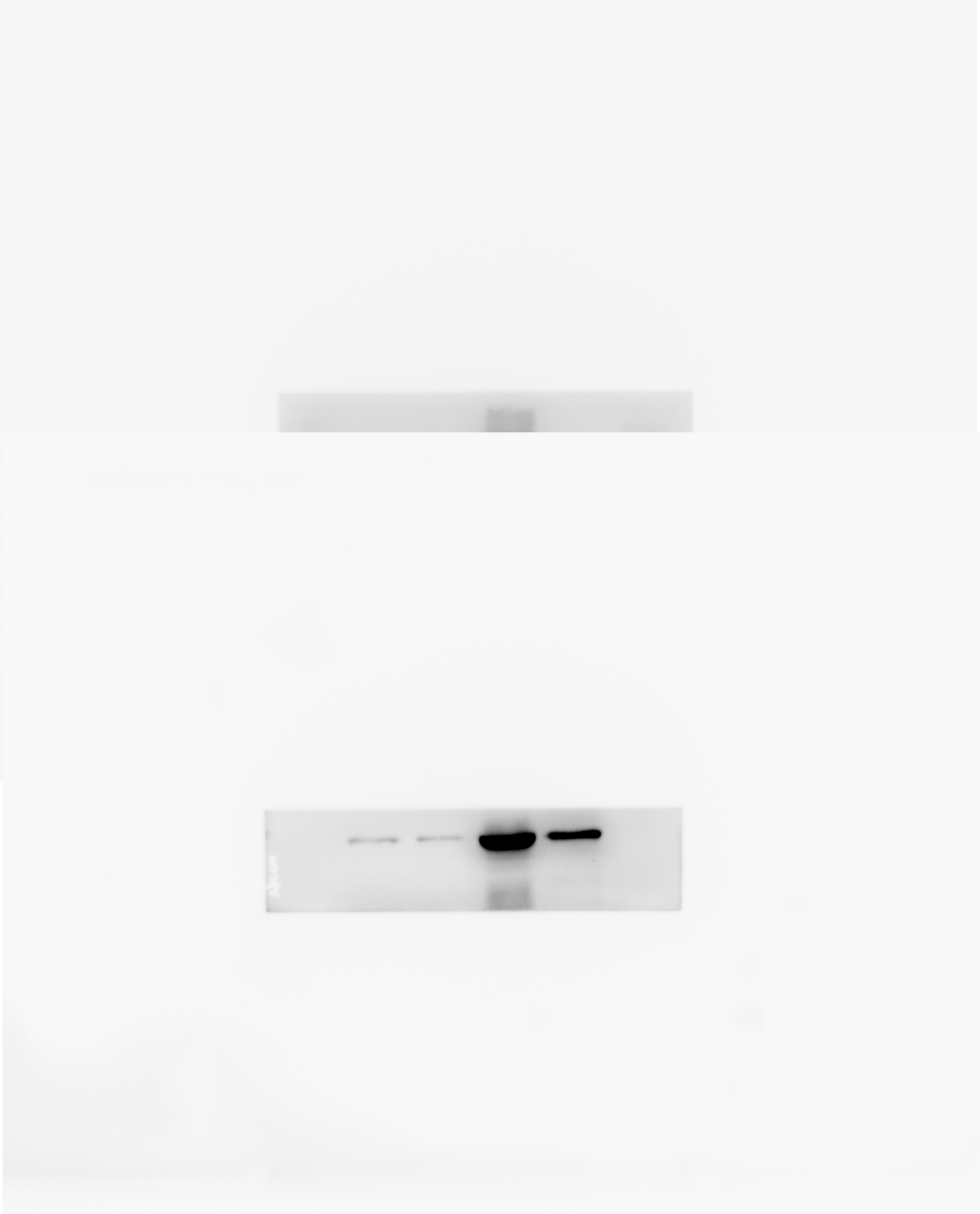

Supplement: Supplementary file 9 — Source data Fig. 3 [file 44321_2025_371_MOESM9_ESM.zip › Figure 3/Fig. 3f/Fig. 3f pull down TSG101.tif]

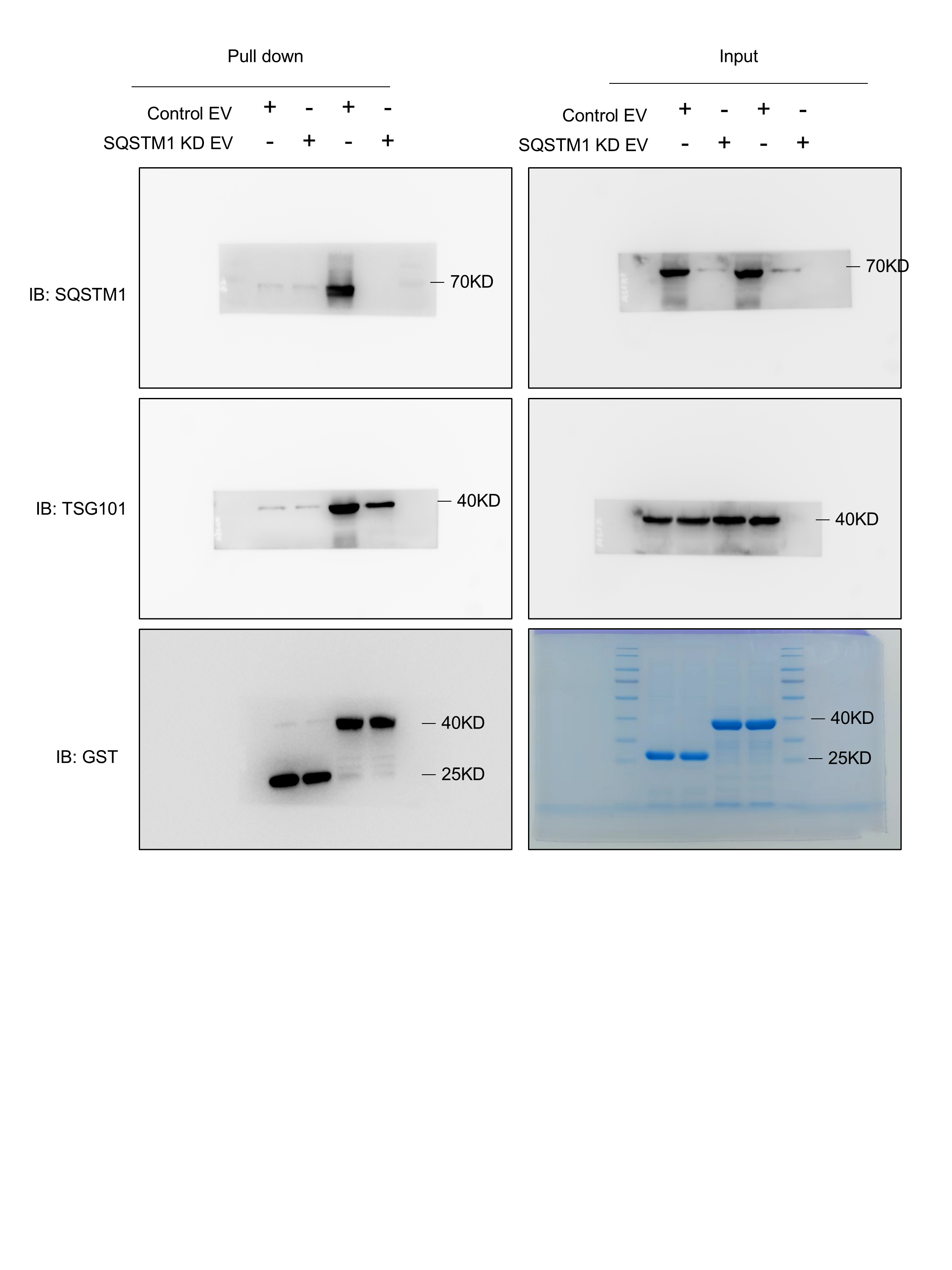

Supplement: Supplementary file 9 — Source data Fig. 3 [file 44321_2025_371_MOESM9_ESM.zip › Figure 3/Fig. 3f/Fig. 3f.tif]

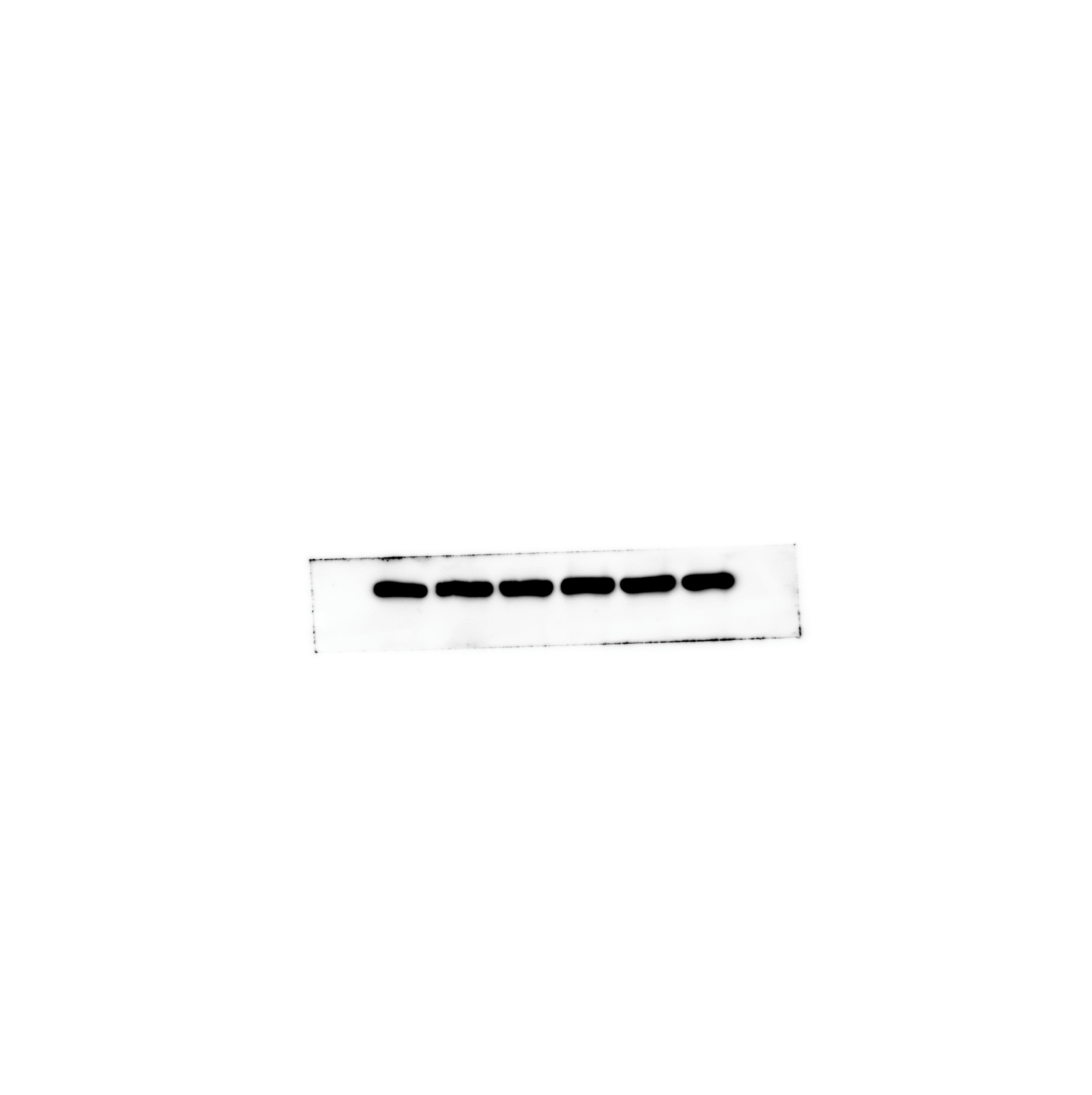

Supplement: Supplementary file 9 — Source data Fig. 3 [file 44321_2025_371_MOESM9_ESM.zip › Figure 3/Fig. 3g/Fig. 3g Deg-actin.tif]

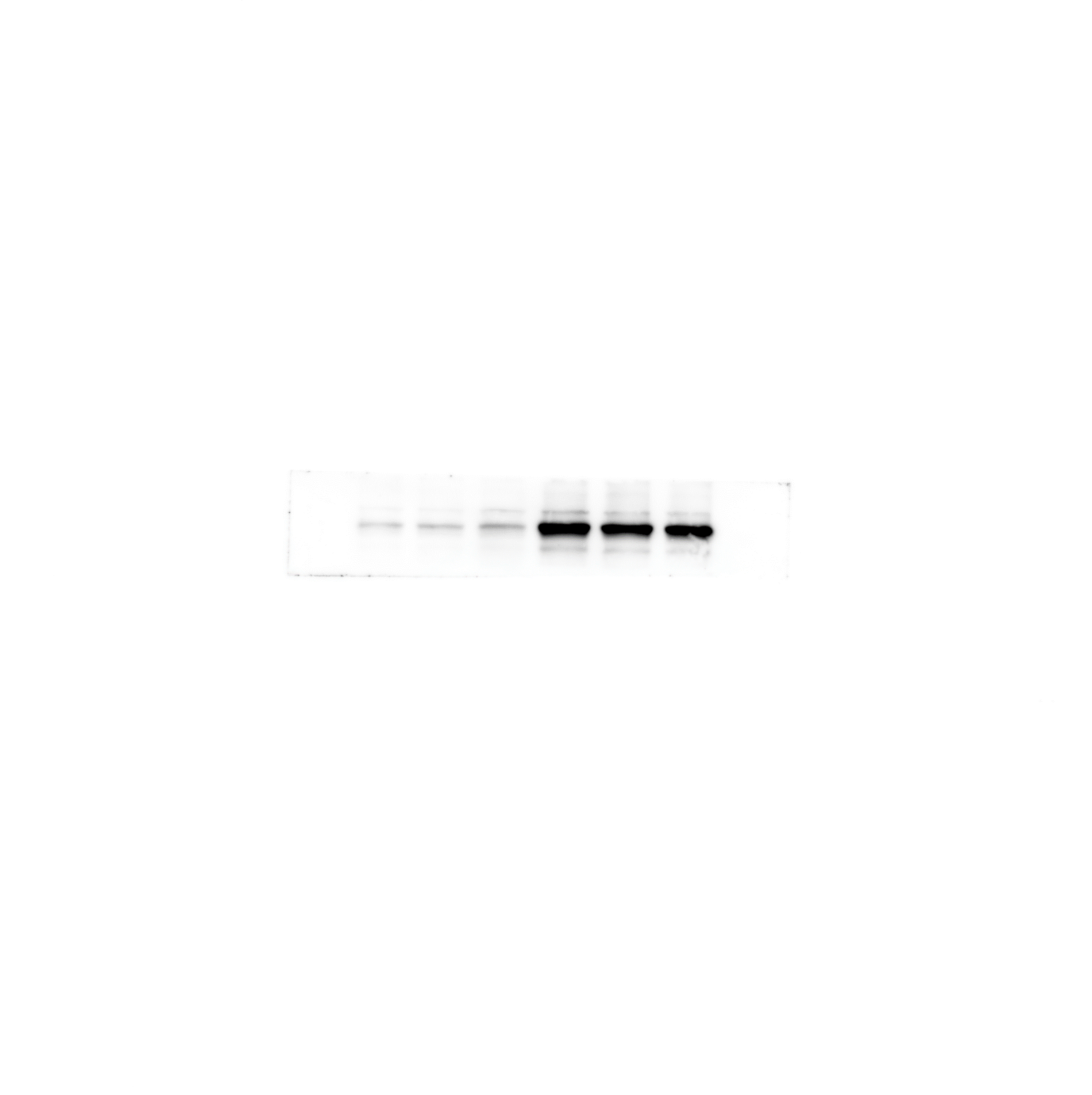

Supplement: Supplementary file 9 — Source data Fig. 3 [file 44321_2025_371_MOESM9_ESM.zip › Figure 3/Fig. 3g/Fig. 3g Deg-GFP.tif]

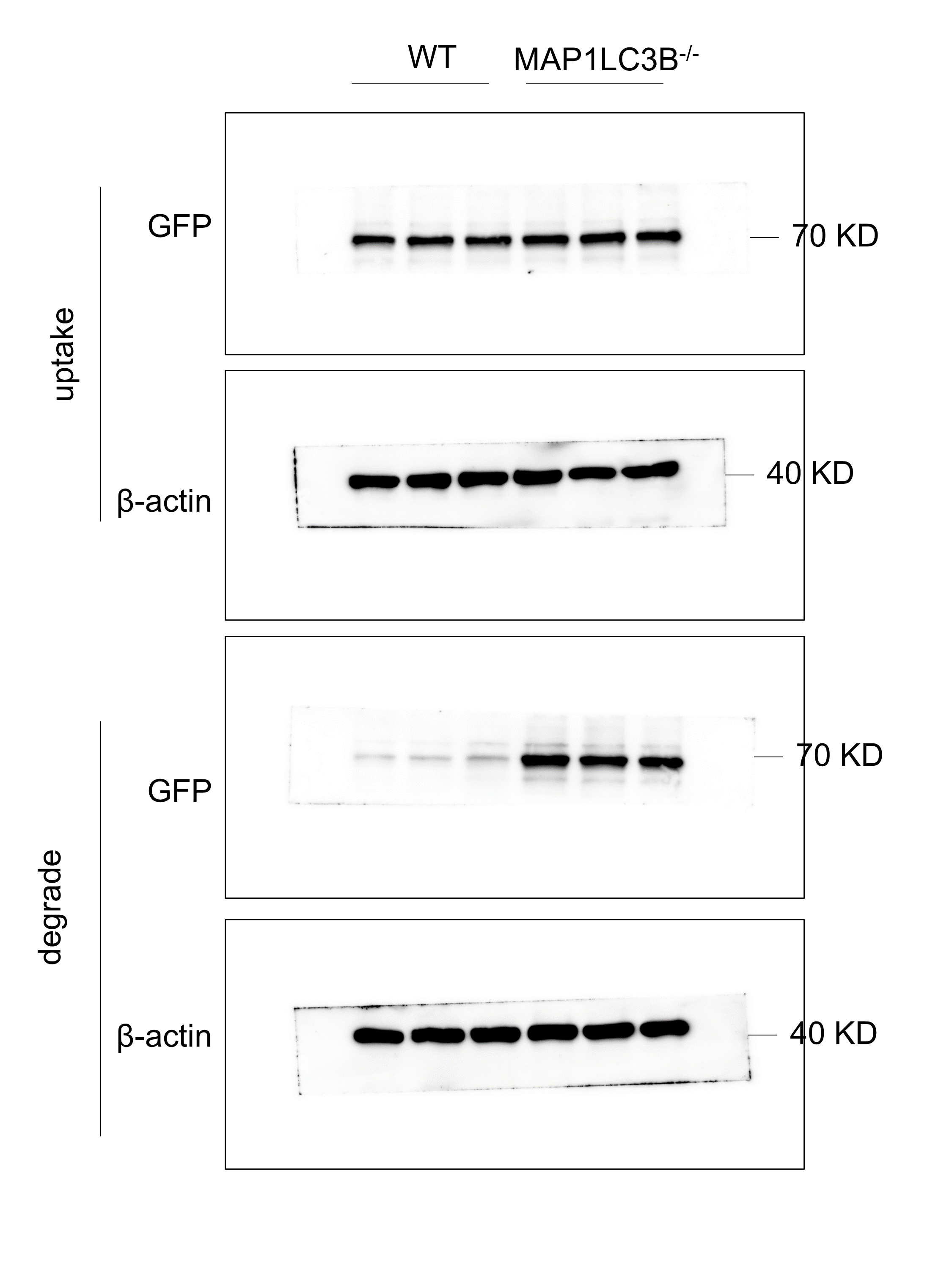

Supplement: Supplementary file 9 — Source data Fig. 3 [file 44321_2025_371_MOESM9_ESM.zip › Figure 3/Fig. 3g/Fig. 3g Summary plus label.tif]

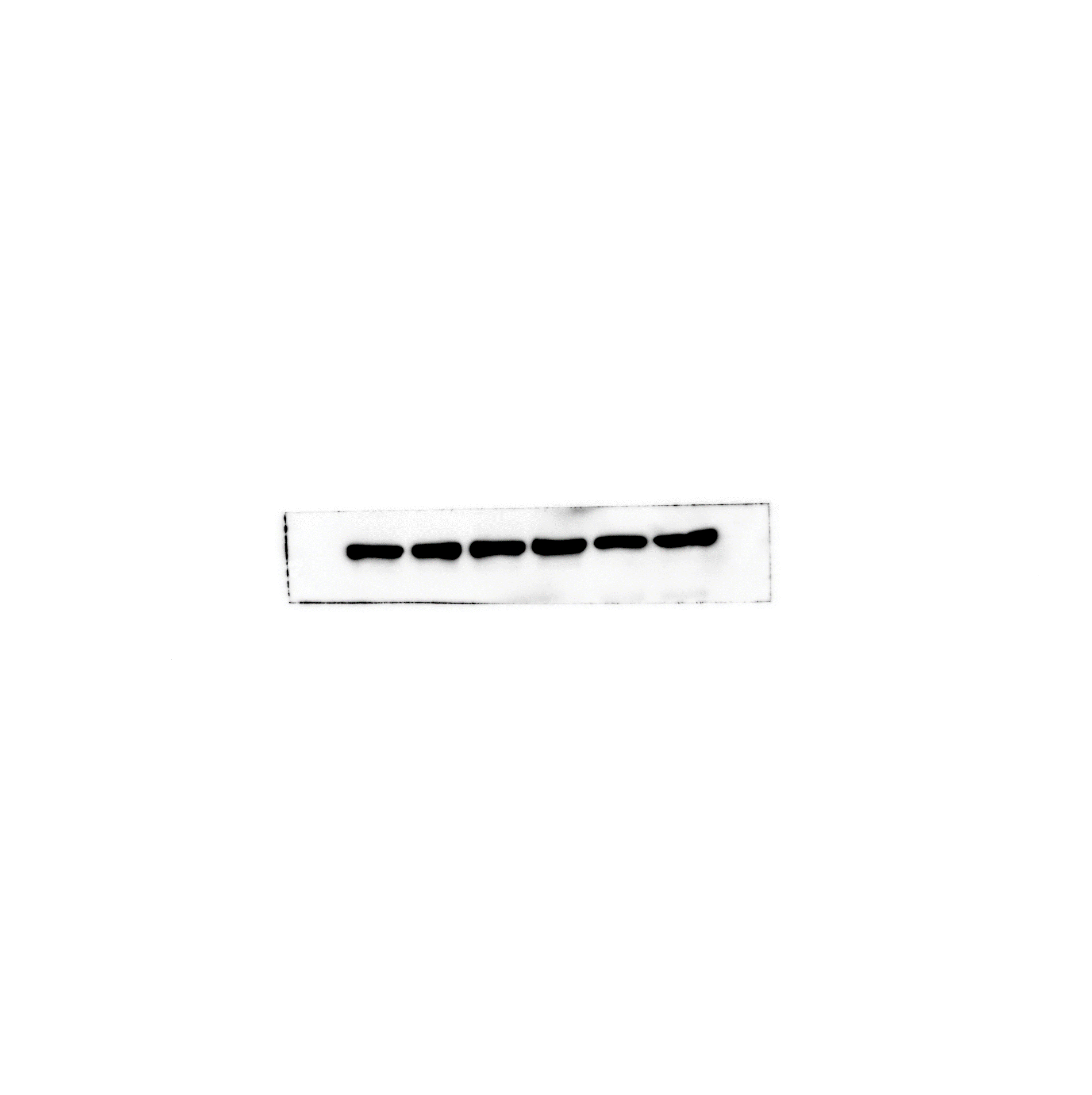

Supplement: Supplementary file 9 — Source data Fig. 3 [file 44321_2025_371_MOESM9_ESM.zip › Figure 3/Fig. 3g/Fig. 3g Uptake-actin.tif]

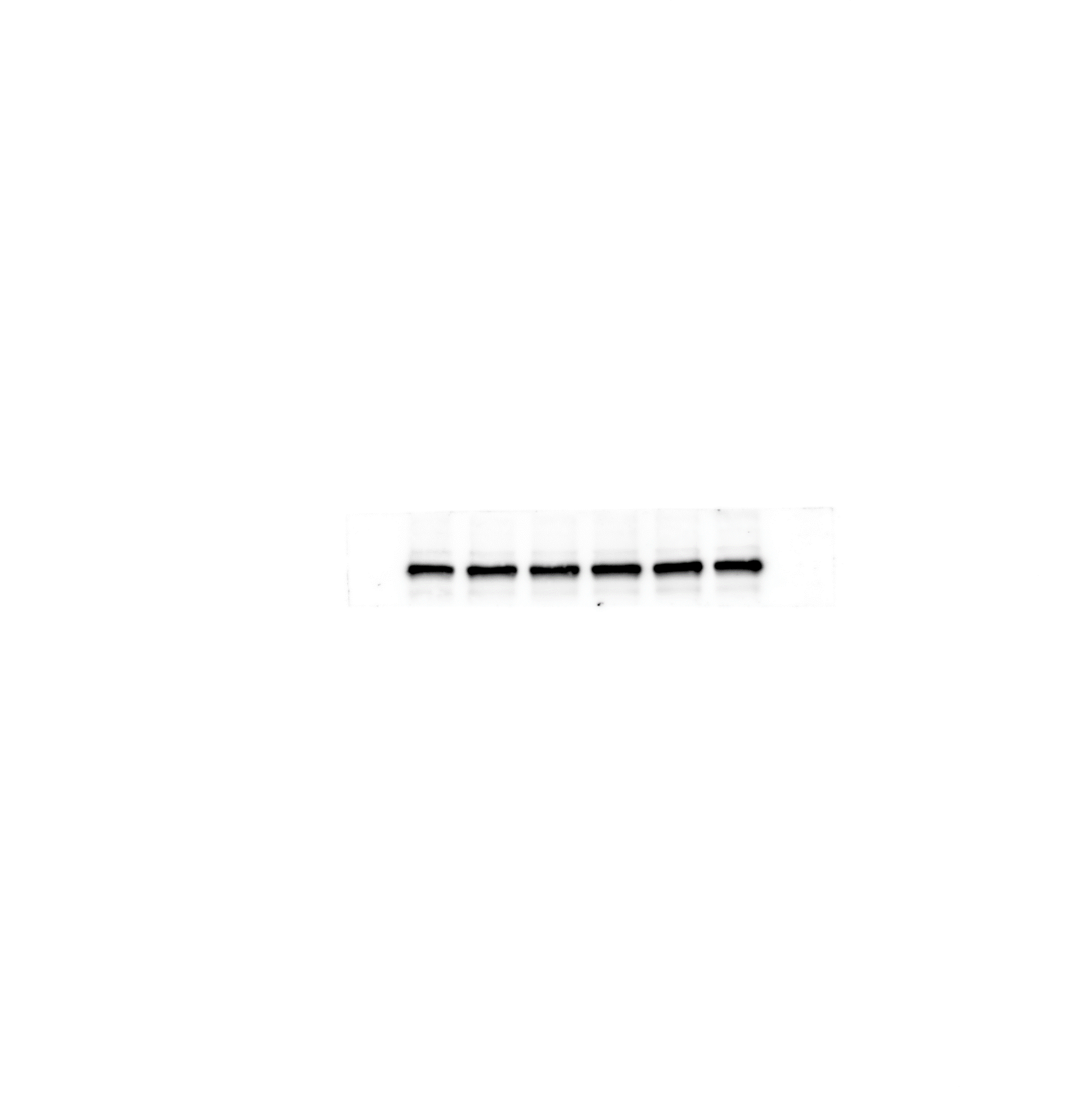

Supplement: Supplementary file 9 — Source data Fig. 3 [file 44321_2025_371_MOESM9_ESM.zip › Figure 3/Fig. 3g/Fig. 3g Uptake-GFP.tif]

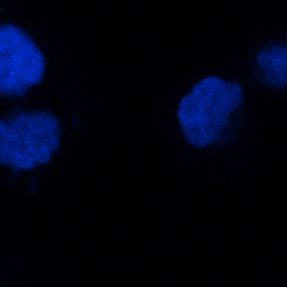

Supplement: Supplementary file 9 — Source data Fig. 3 [file 44321_2025_371_MOESM9_ESM.zip › Figure 3/Fig. 3h/Fig. 3h 1-DAPI.tif]

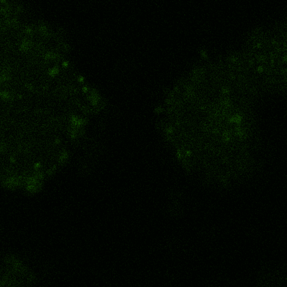

Supplement: Supplementary file 9 — Source data Fig. 3 [file 44321_2025_371_MOESM9_ESM.zip › Figure 3/Fig. 3h/Fig. 3h 1-EEA1.tif]

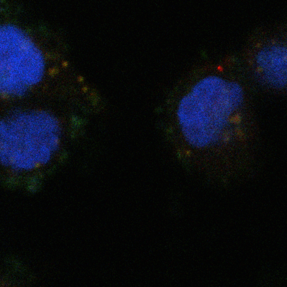

Supplement: Supplementary file 9 — Source data Fig. 3 [file 44321_2025_371_MOESM9_ESM.zip › Figure 3/Fig. 3h/Fig. 3h 1-Merge.tif]

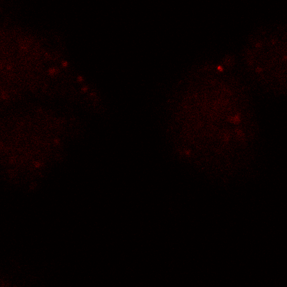

Supplement: Supplementary file 9 — Source data Fig. 3 [file 44321_2025_371_MOESM9_ESM.zip › Figure 3/Fig. 3h/Fig. 3h 1-PLA.tif]

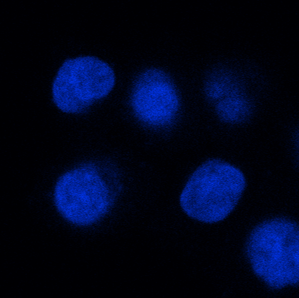

Supplement: Supplementary file 9 — Source data Fig. 3 [file 44321_2025_371_MOESM9_ESM.zip › Figure 3/Fig. 3h/Fig. 3h 2-DAPI.tif]

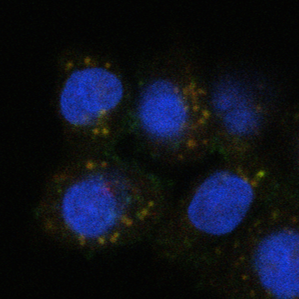

Supplement: Supplementary file 9 — Source data Fig. 3 [file 44321_2025_371_MOESM9_ESM.zip › Figure 3/Fig. 3h/Fig. 3h 2-Merge.tif]

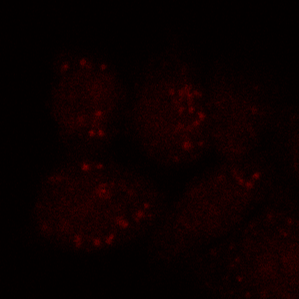

Supplement: Supplementary file 9 — Source data Fig. 3 [file 44321_2025_371_MOESM9_ESM.zip › Figure 3/Fig. 3h/Fig. 3h 2-PLA.tif]

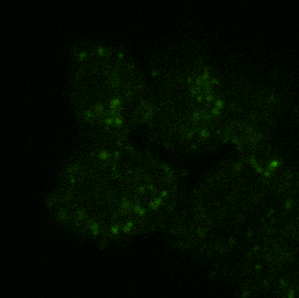

Supplement: Supplementary file 9 — Source data Fig. 3 [file 44321_2025_371_MOESM9_ESM.zip › Figure 3/Fig. 3h/Fig. 3h 2-Rab7a.tif]

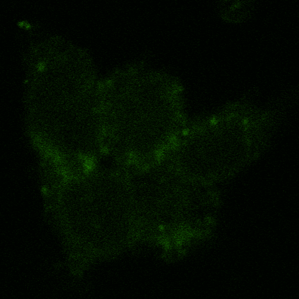

Supplement: Supplementary file 9 — Source data Fig. 3 [file 44321_2025_371_MOESM9_ESM.zip › Figure 3/Fig. 3h/Fig. 3h 3-Calreticulin.tif]

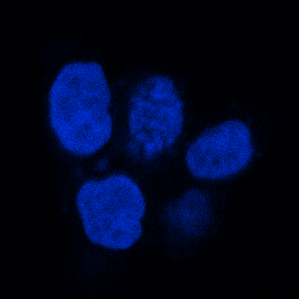

Supplement: Supplementary file 9 — Source data Fig. 3 [file 44321_2025_371_MOESM9_ESM.zip › Figure 3/Fig. 3h/Fig. 3h 3-DAPI.tif]

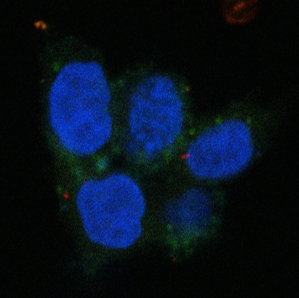

Supplement: Supplementary file 9 — Source data Fig. 3 [file 44321_2025_371_MOESM9_ESM.zip › Figure 3/Fig. 3h/Fig. 3h 3-Merge.tif]

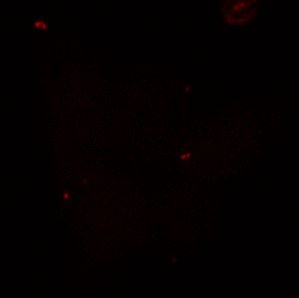

Supplement: Supplementary file 9 — Source data Fig. 3 [file 44321_2025_371_MOESM9_ESM.zip › Figure 3/Fig. 3h/Fig. 3h 3-PLA.tif]

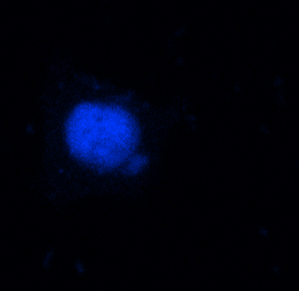

Supplement: Supplementary file 9 — Source data Fig. 3 [file 44321_2025_371_MOESM9_ESM.zip › Figure 3/Fig. 3i/Fig. 3i ATG7KO-DAPI.tif]

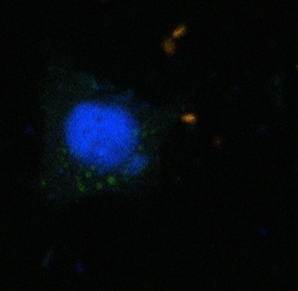

Supplement: Supplementary file 9 — Source data Fig. 3 [file 44321_2025_371_MOESM9_ESM.zip › Figure 3/Fig. 3i/Fig. 3i ATG7KO-Merge.tif]

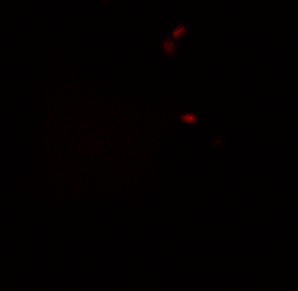

Supplement: Supplementary file 9 — Source data Fig. 3 [file 44321_2025_371_MOESM9_ESM.zip › Figure 3/Fig. 3i/Fig. 3i ATG7KO-PLA.tif]

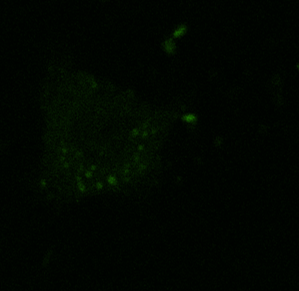

Supplement: Supplementary file 9 — Source data Fig. 3 [file 44321_2025_371_MOESM9_ESM.zip › Figure 3/Fig. 3i/Fig. 3i ATG7KO-Rab7a.tif]

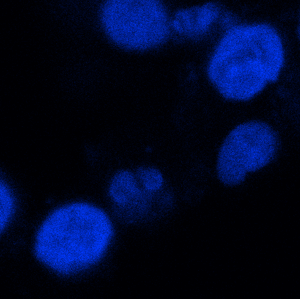

Supplement: Supplementary file 9 — Source data Fig. 3 [file 44321_2025_371_MOESM9_ESM.zip › Figure 3/Fig. 3i/Fig. 3i WT-DAPI.tif]

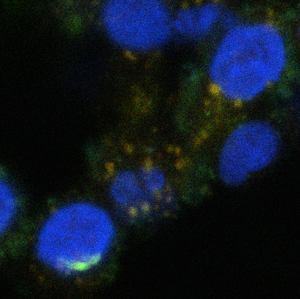

Supplement: Supplementary file 9 — Source data Fig. 3 [file 44321_2025_371_MOESM9_ESM.zip › Figure 3/Fig. 3i/Fig. 3i WT-Merge.tif]

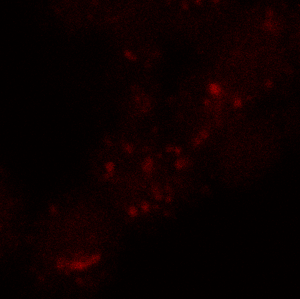

Supplement: Supplementary file 9 — Source data Fig. 3 [file 44321_2025_371_MOESM9_ESM.zip › Figure 3/Fig. 3i/Fig. 3i WT-PLA.tif]

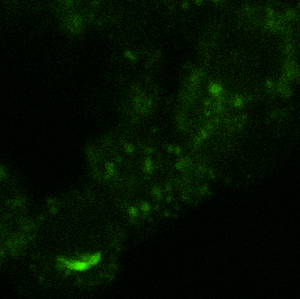

Supplement: Supplementary file 9 — Source data Fig. 3 [file 44321_2025_371_MOESM9_ESM.zip › Figure 3/Fig. 3i/Fig. 3i WT-Rab7a.tif]

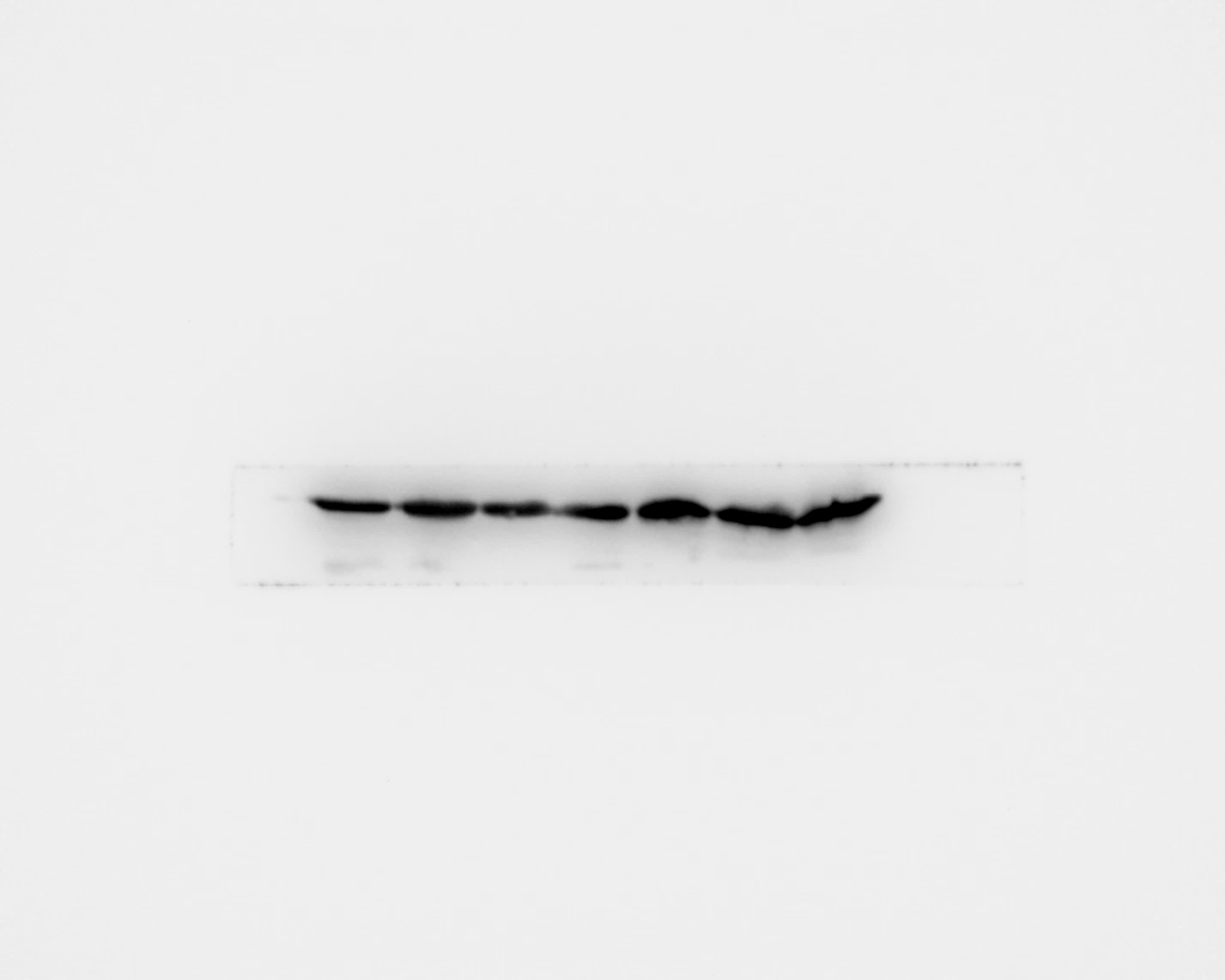

Supplement: Supplementary file 9 — Source data Fig. 3 [file 44321_2025_371_MOESM9_ESM.zip › Figure 3/Fig. 3k/Fig. 3k Deg-actin.tif]

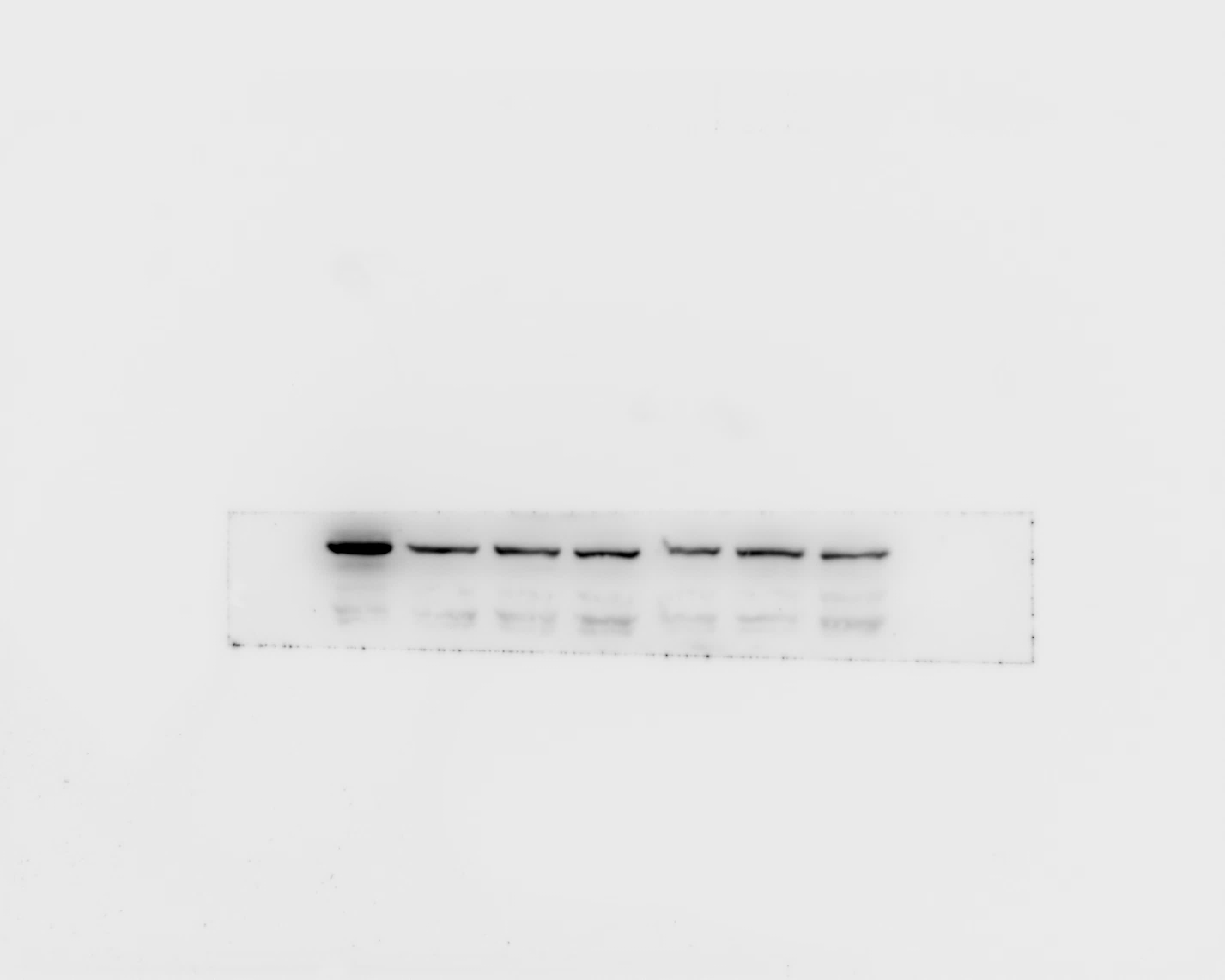

Supplement: Supplementary file 9 — Source data Fig. 3 [file 44321_2025_371_MOESM9_ESM.zip › Figure 3/Fig. 3k/Fig. 3k Deg-GFP.tif]

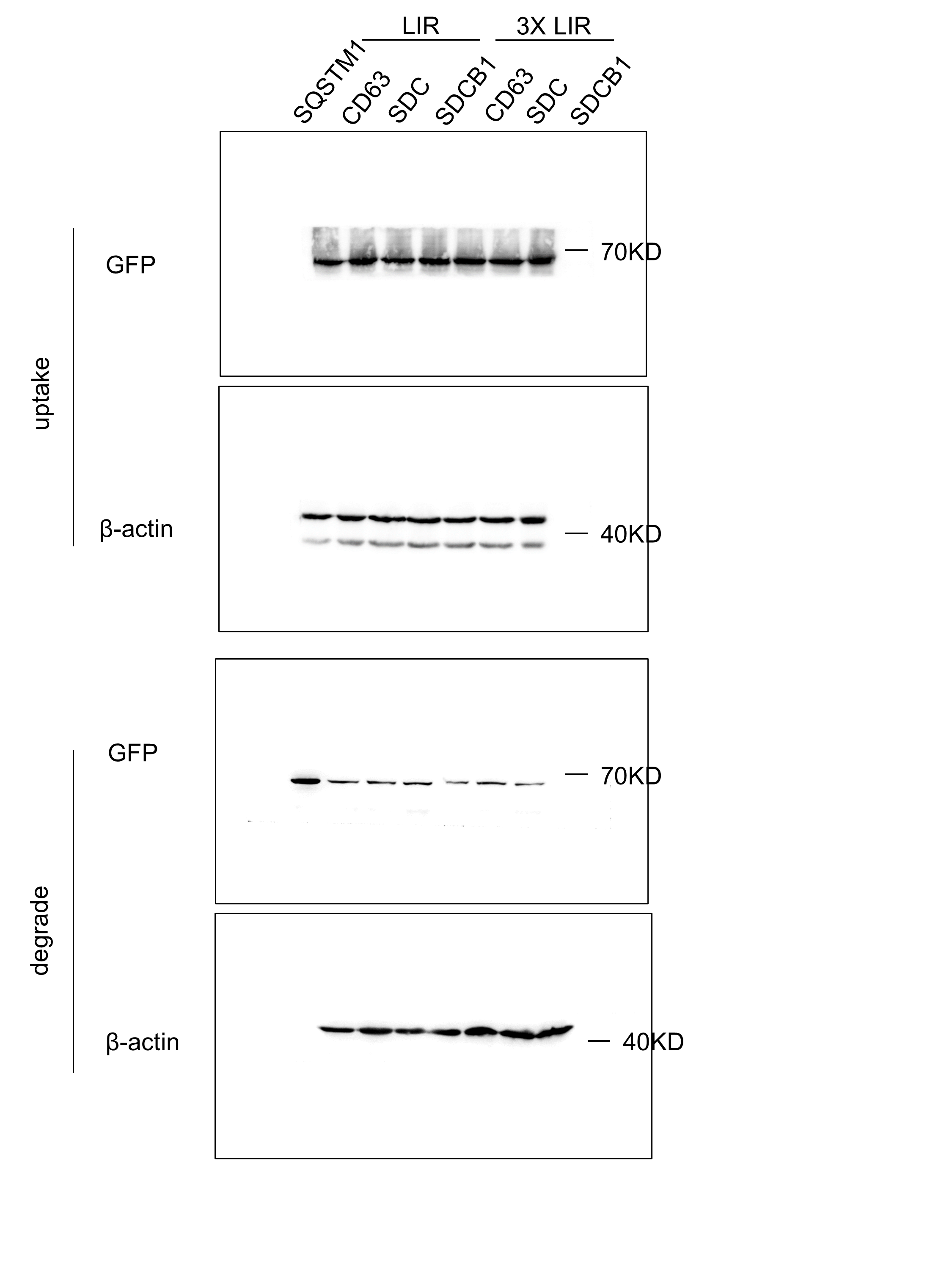

Supplement: Supplementary file 9 — Source data Fig. 3 [file 44321_2025_371_MOESM9_ESM.zip › Figure 3/Fig. 3k/Fig. 3k Summary plus label.tif]

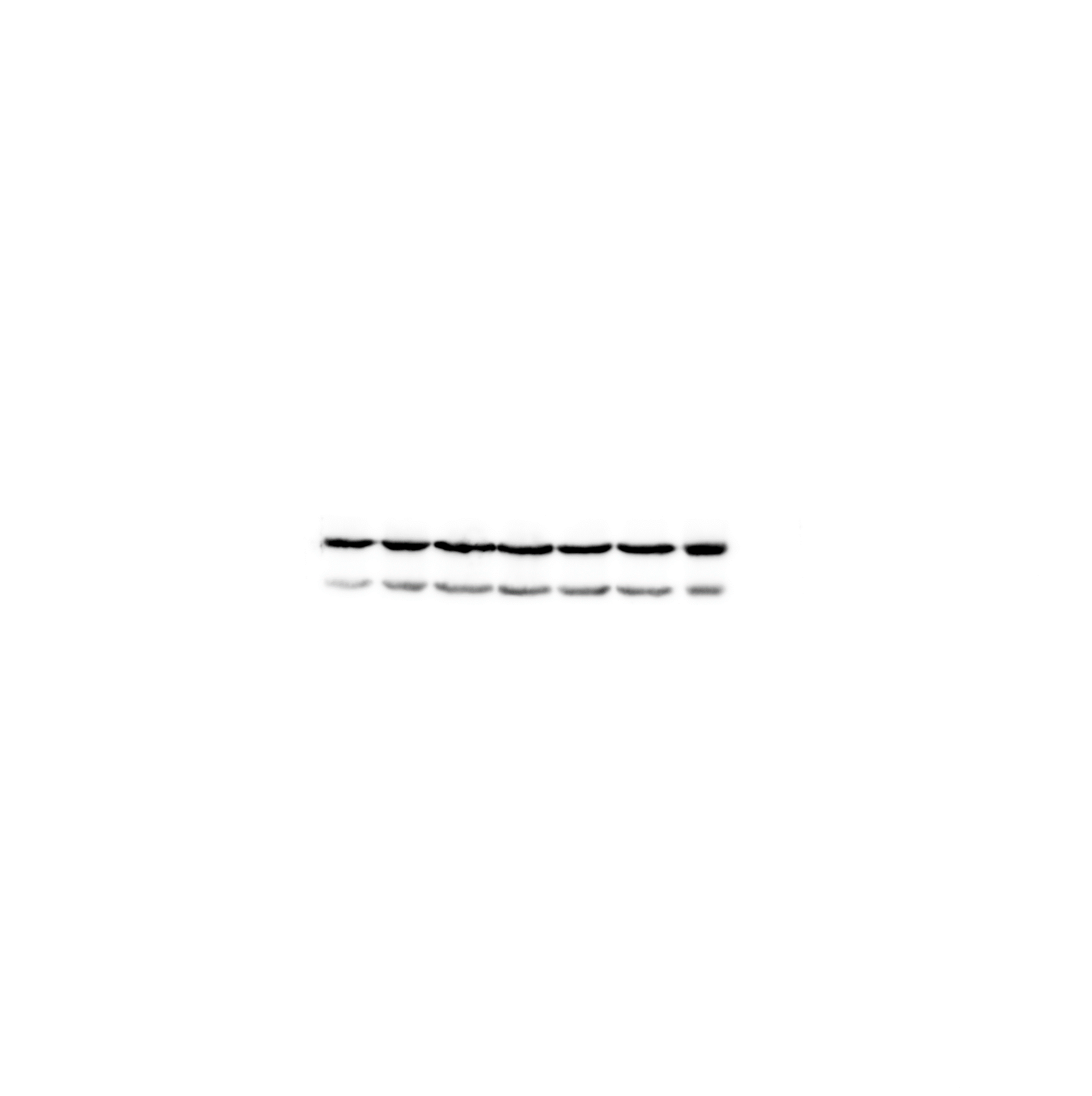

Supplement: Supplementary file 9 — Source data Fig. 3 [file 44321_2025_371_MOESM9_ESM.zip › Figure 3/Fig. 3k/Fig. 3k Uptake-actin.tif]

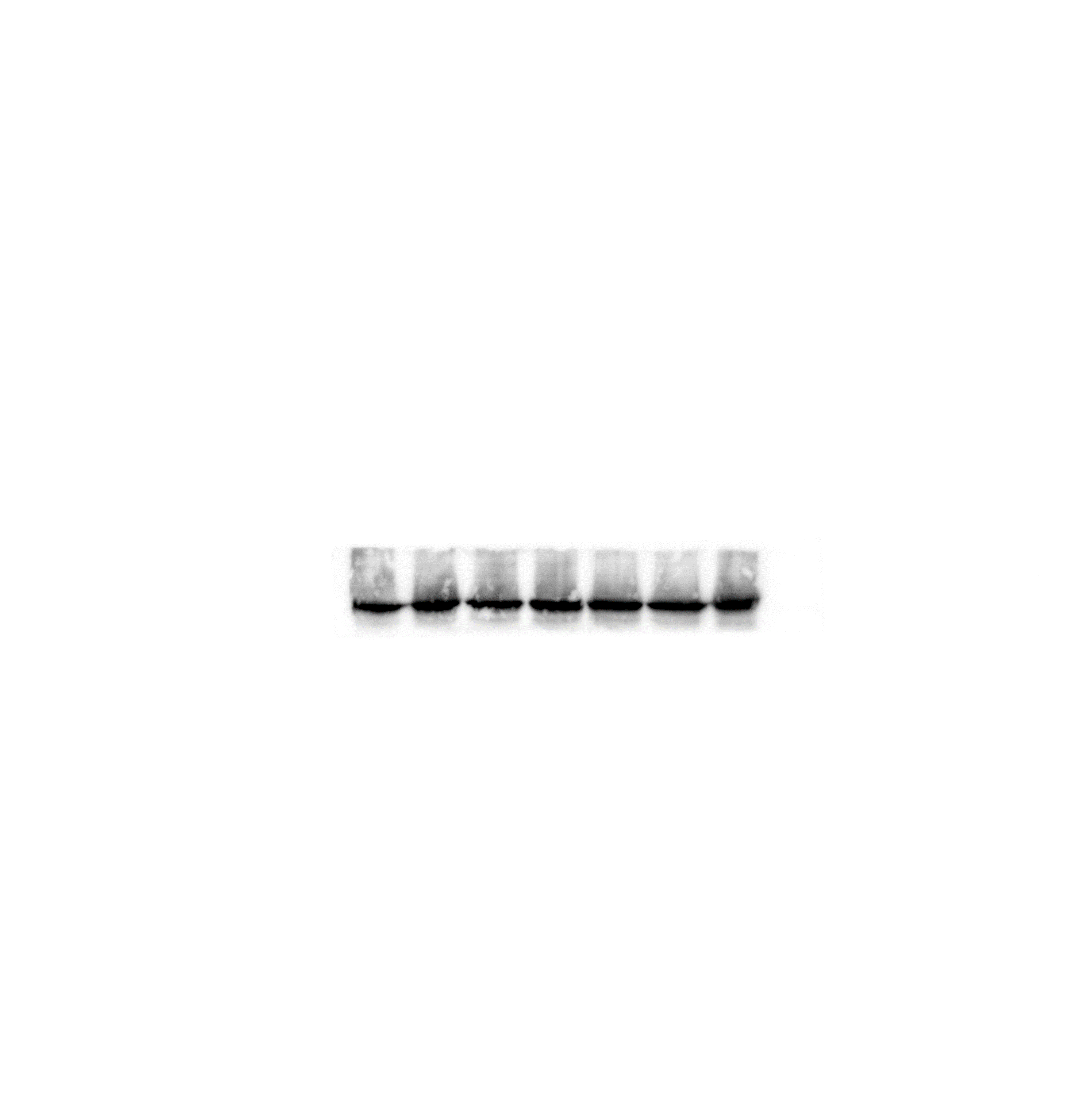

Supplement: Supplementary file 9 — Source data Fig. 3 [file 44321_2025_371_MOESM9_ESM.zip › Figure 3/Fig. 3k/Fig. 3k Uptake-GFP.tif]

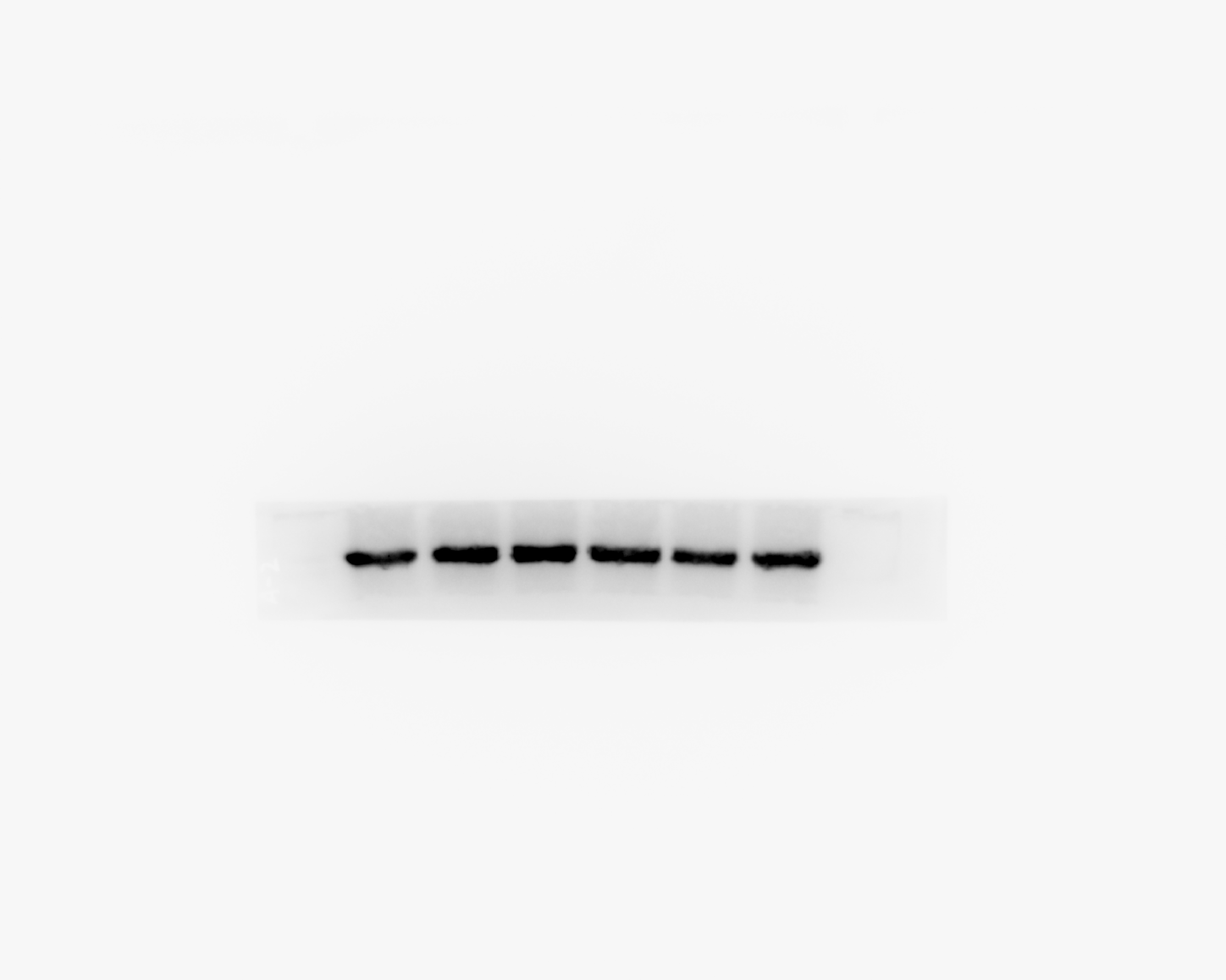

Supplement: Supplementary file 10 — Source data Fig. 4 [file 44321_2025_371_MOESM10_ESM.zip › Figure 4/Fig. 4g/Fig. 4g Cell lysate-actin.tif]

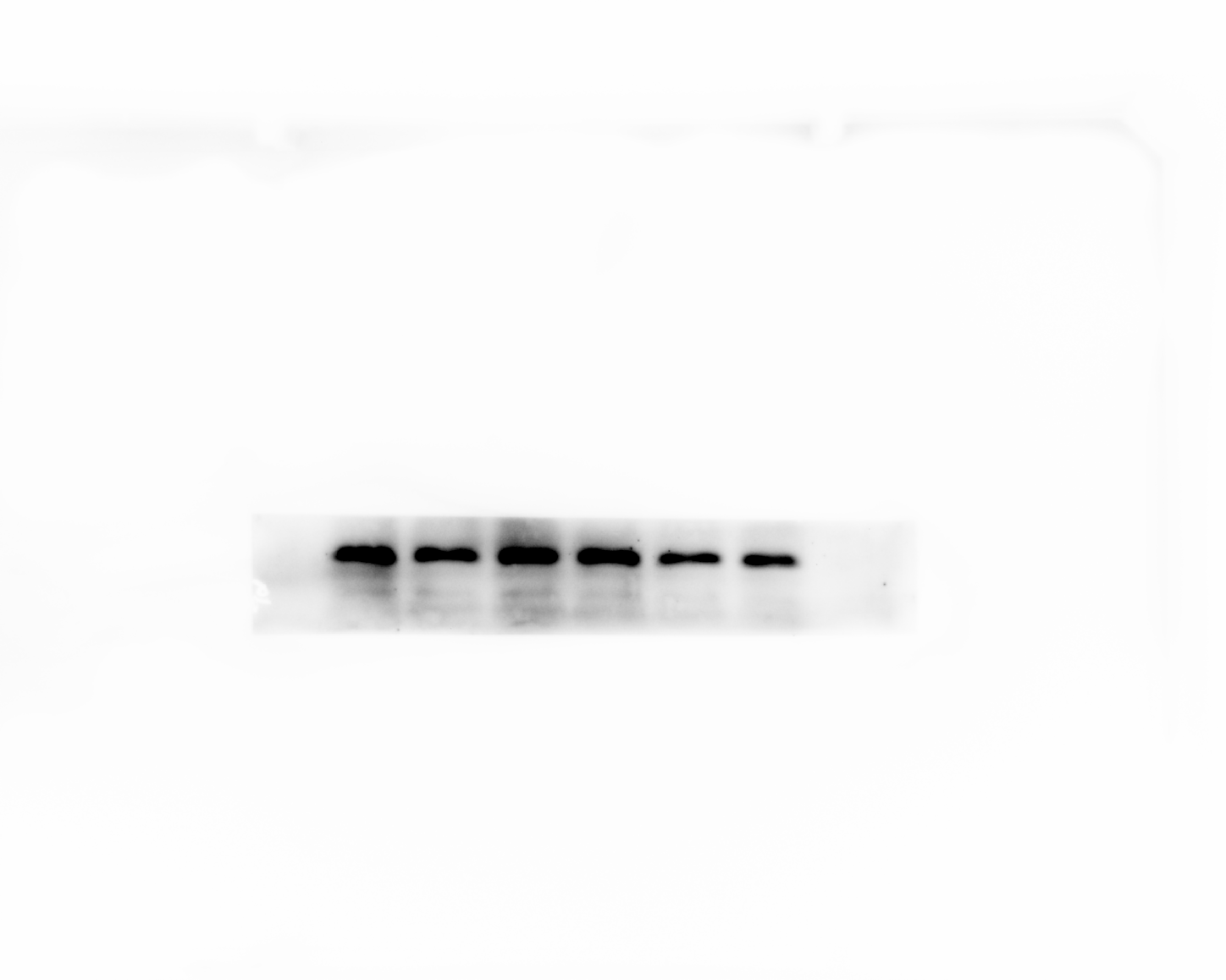

Supplement: Supplementary file 10 — Source data Fig. 4 [file 44321_2025_371_MOESM10_ESM.zip › Figure 4/Fig. 4g/Fig. 4g Cell lysate-TNFa.tif]

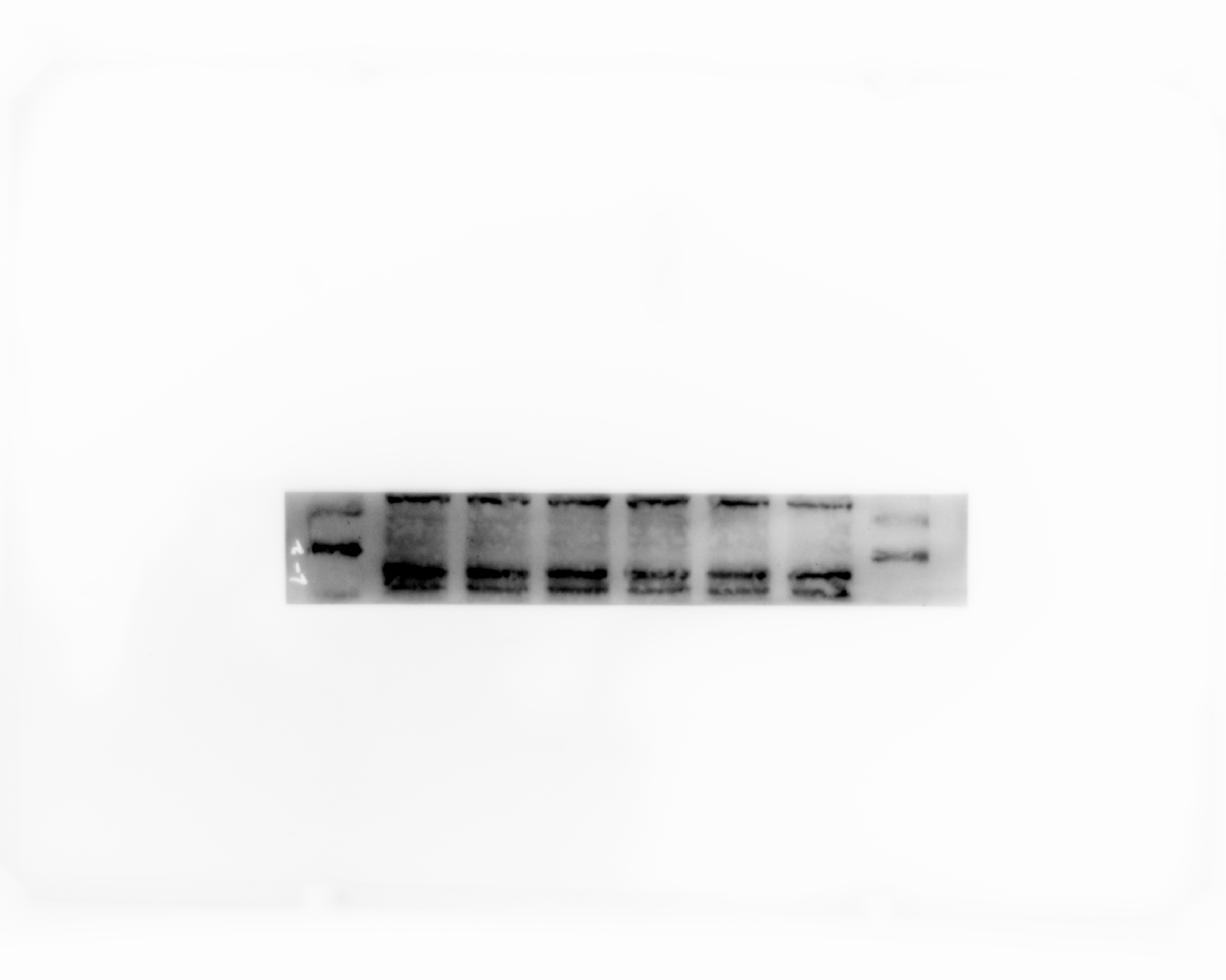

Supplement: Supplementary file 10 — Source data Fig. 4 [file 44321_2025_371_MOESM10_ESM.zip › Figure 4/Fig. 4g/Fig. 4g Lysosome-LampII.tif]

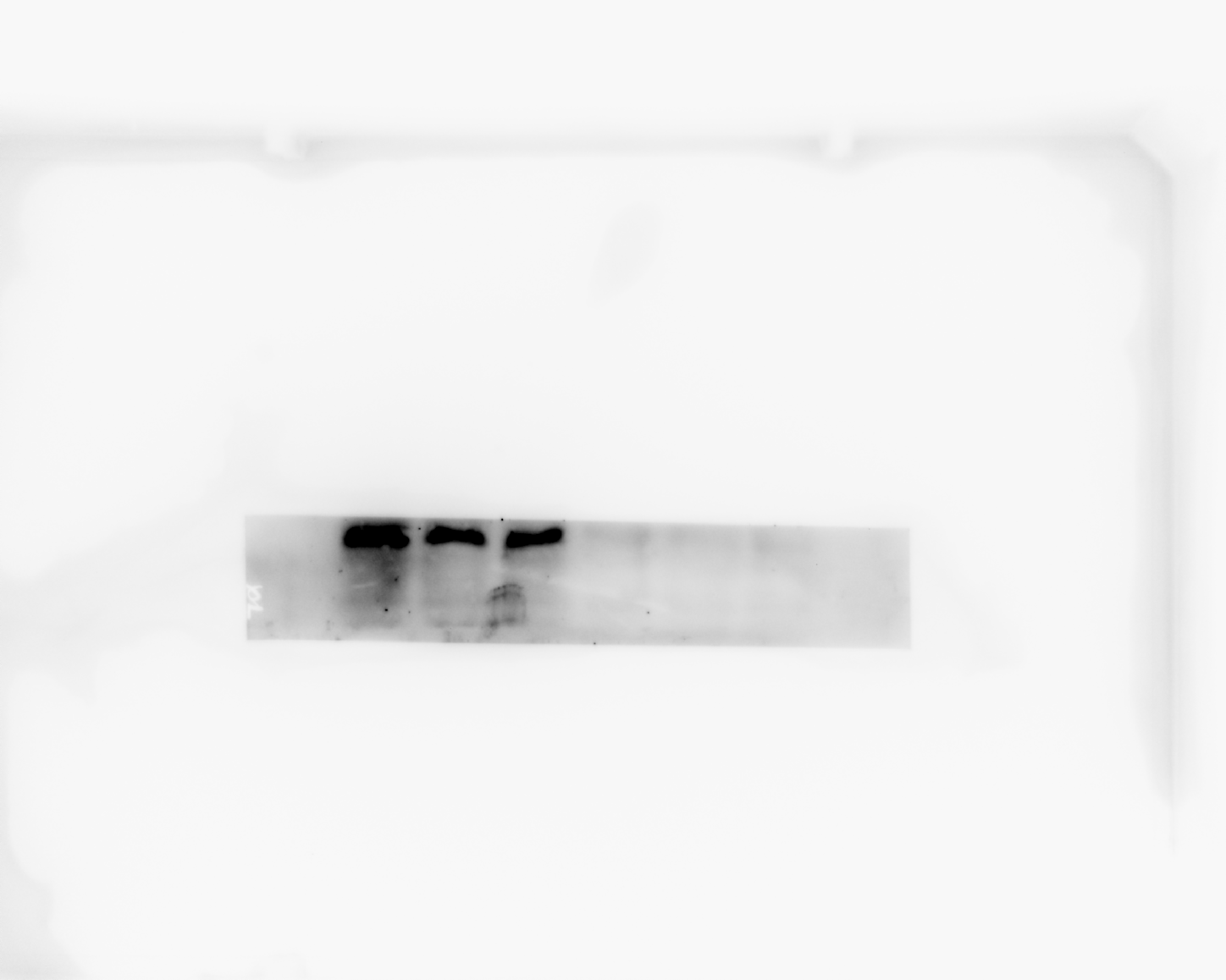

Supplement: Supplementary file 10 — Source data Fig. 4 [file 44321_2025_371_MOESM10_ESM.zip › Figure 4/Fig. 4g/Fig. 4g Lysosome-TNFa.tif]

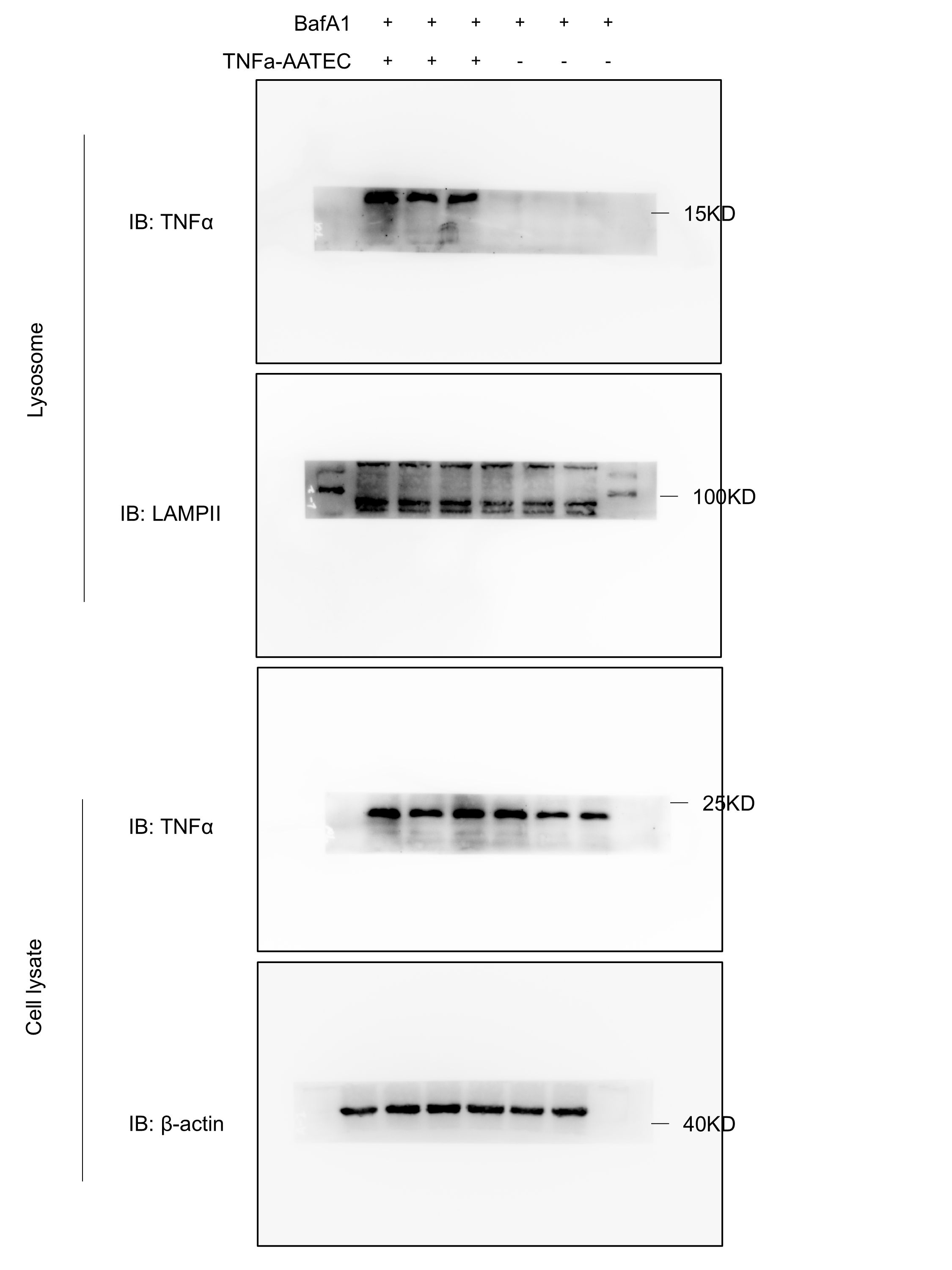

Supplement: Supplementary file 10 — Source data Fig. 4 [file 44321_2025_371_MOESM10_ESM.zip › Figure 4/Fig. 4g/Fig. 4g Summary plus label.tif]

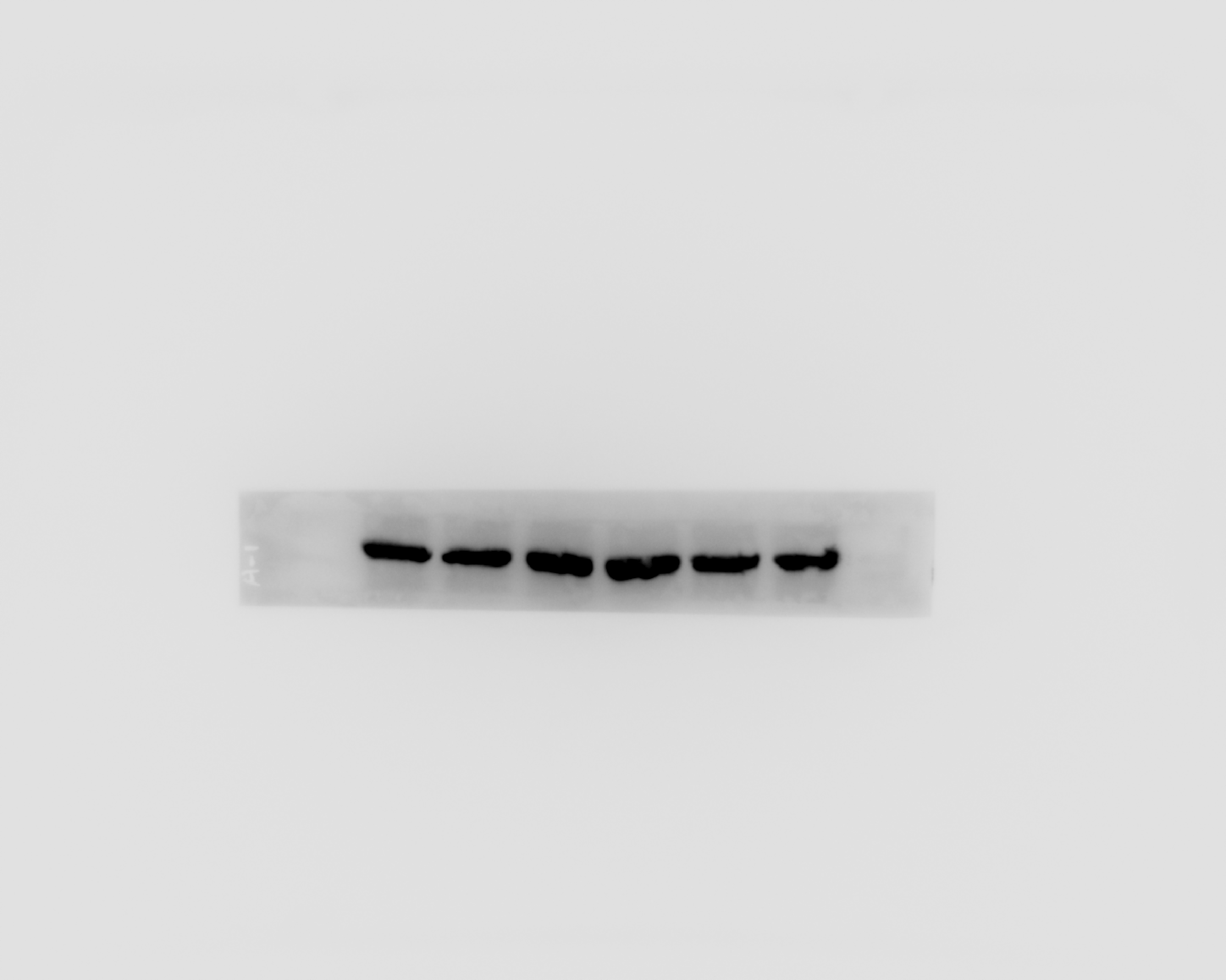

Supplement: Supplementary file 10 — Source data Fig. 4 [file 44321_2025_371_MOESM10_ESM.zip › Figure 4/Fig. 4n/Fig. 4n Cell lysate-actin.tif]

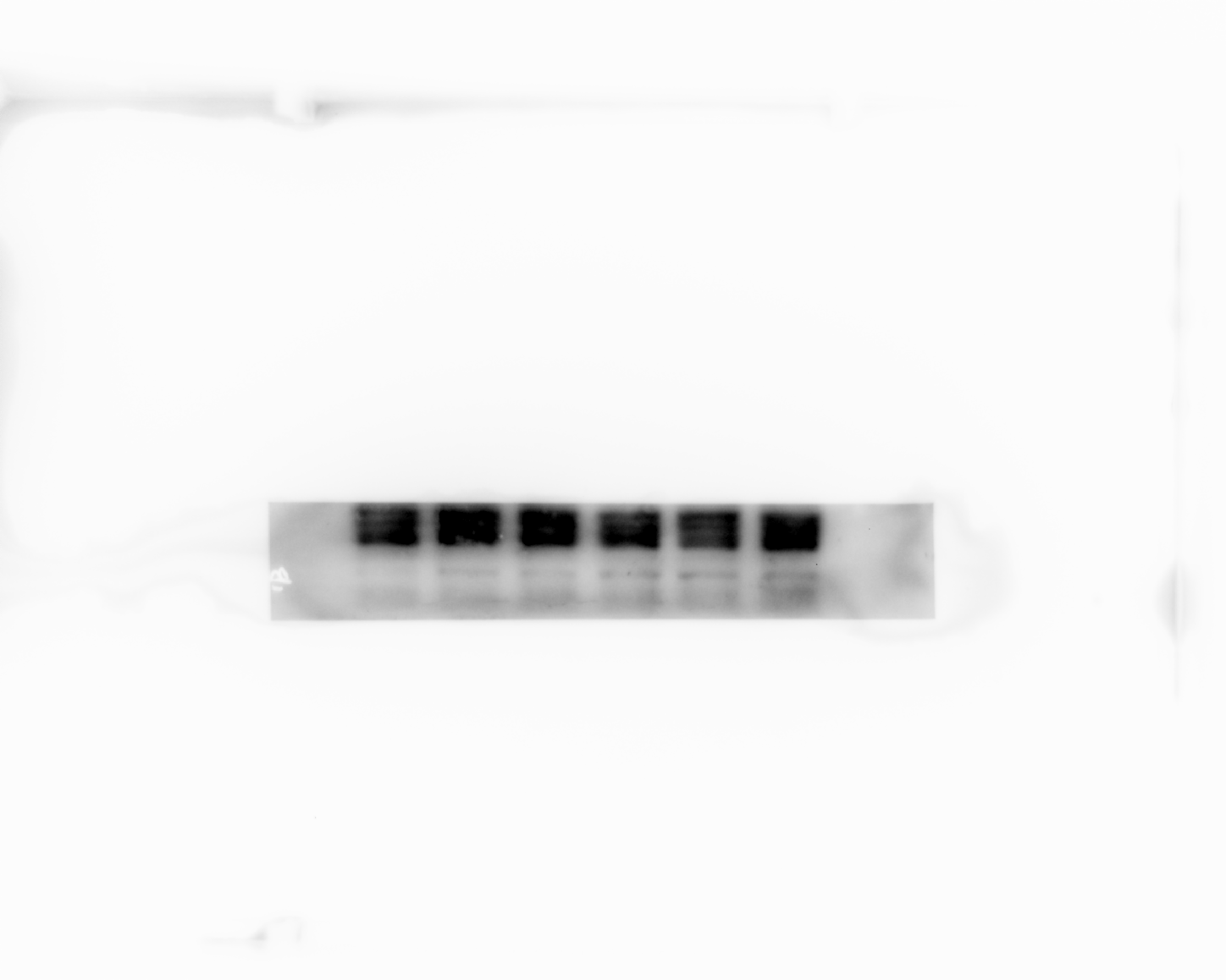

Supplement: Supplementary file 10 — Source data Fig. 4 [file 44321_2025_371_MOESM10_ESM.zip › Figure 4/Fig. 4n/Fig. 4n Cell lysate-IL1b.tif]

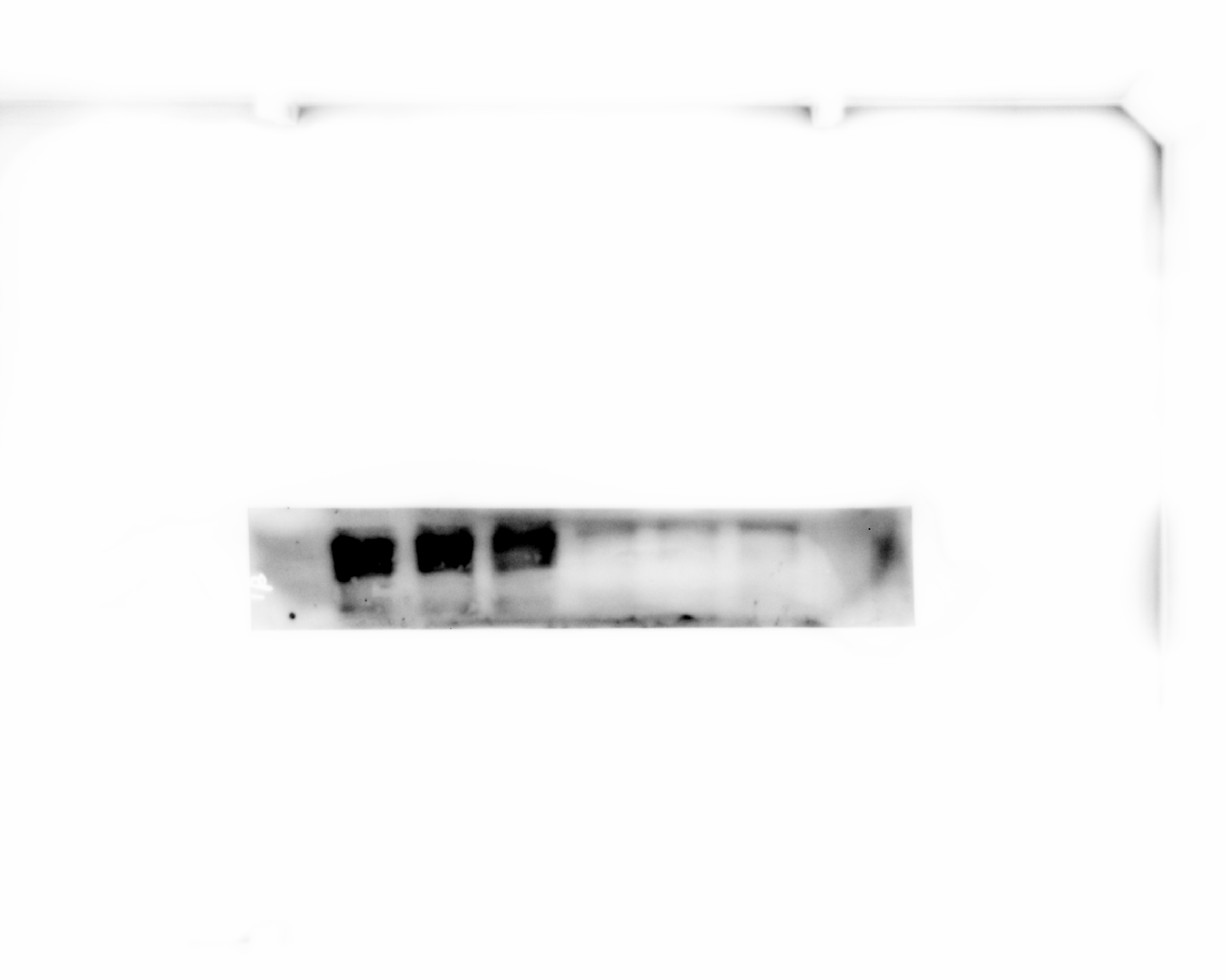

Supplement: Supplementary file 10 — Source data Fig. 4 [file 44321_2025_371_MOESM10_ESM.zip › Figure 4/Fig. 4n/Fig. 4n Lysosome-IL1b.tif]

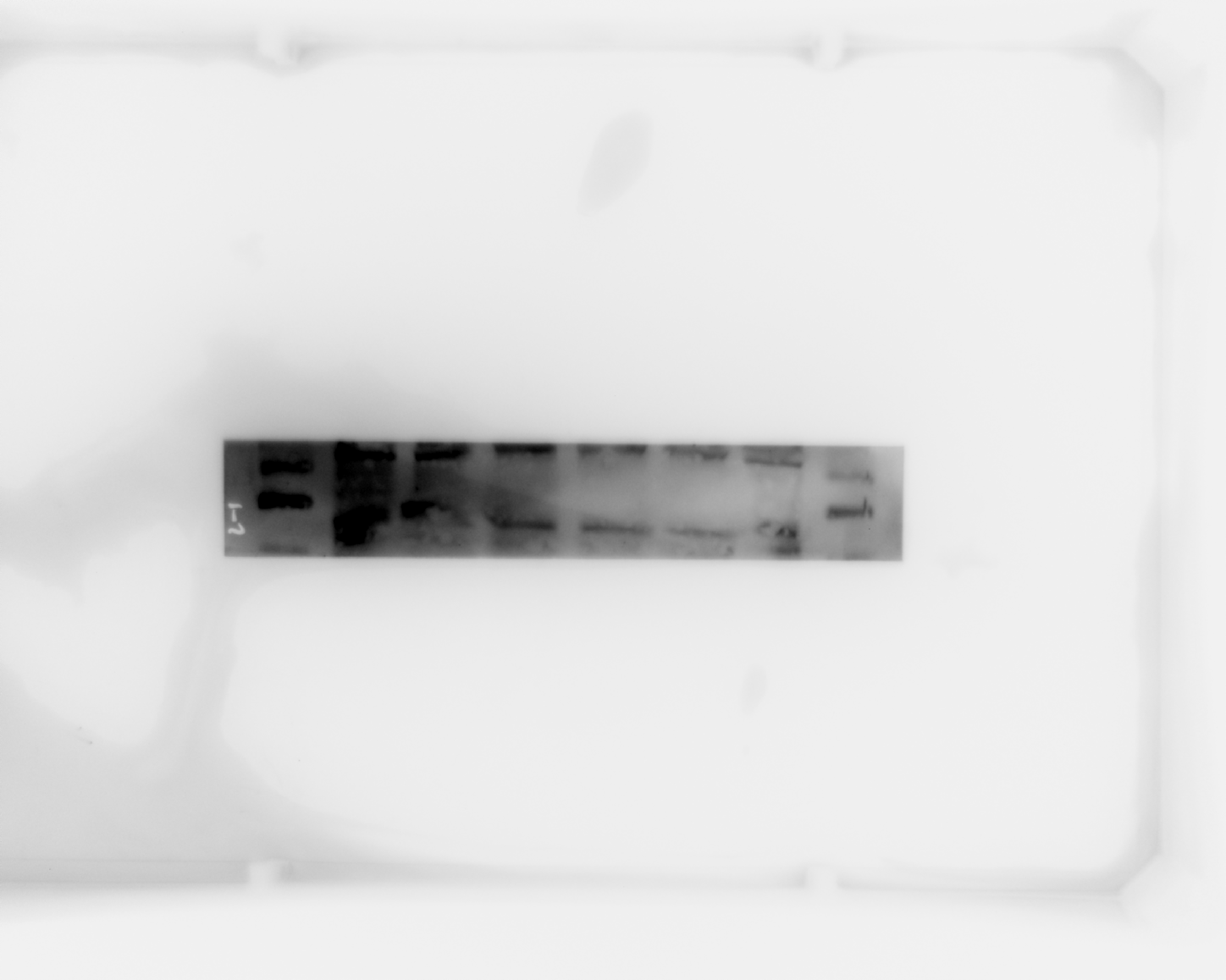

Supplement: Supplementary file 10 — Source data Fig. 4 [file 44321_2025_371_MOESM10_ESM.zip › Figure 4/Fig. 4n/Fig. 4n Lysosome-LampII.tif]

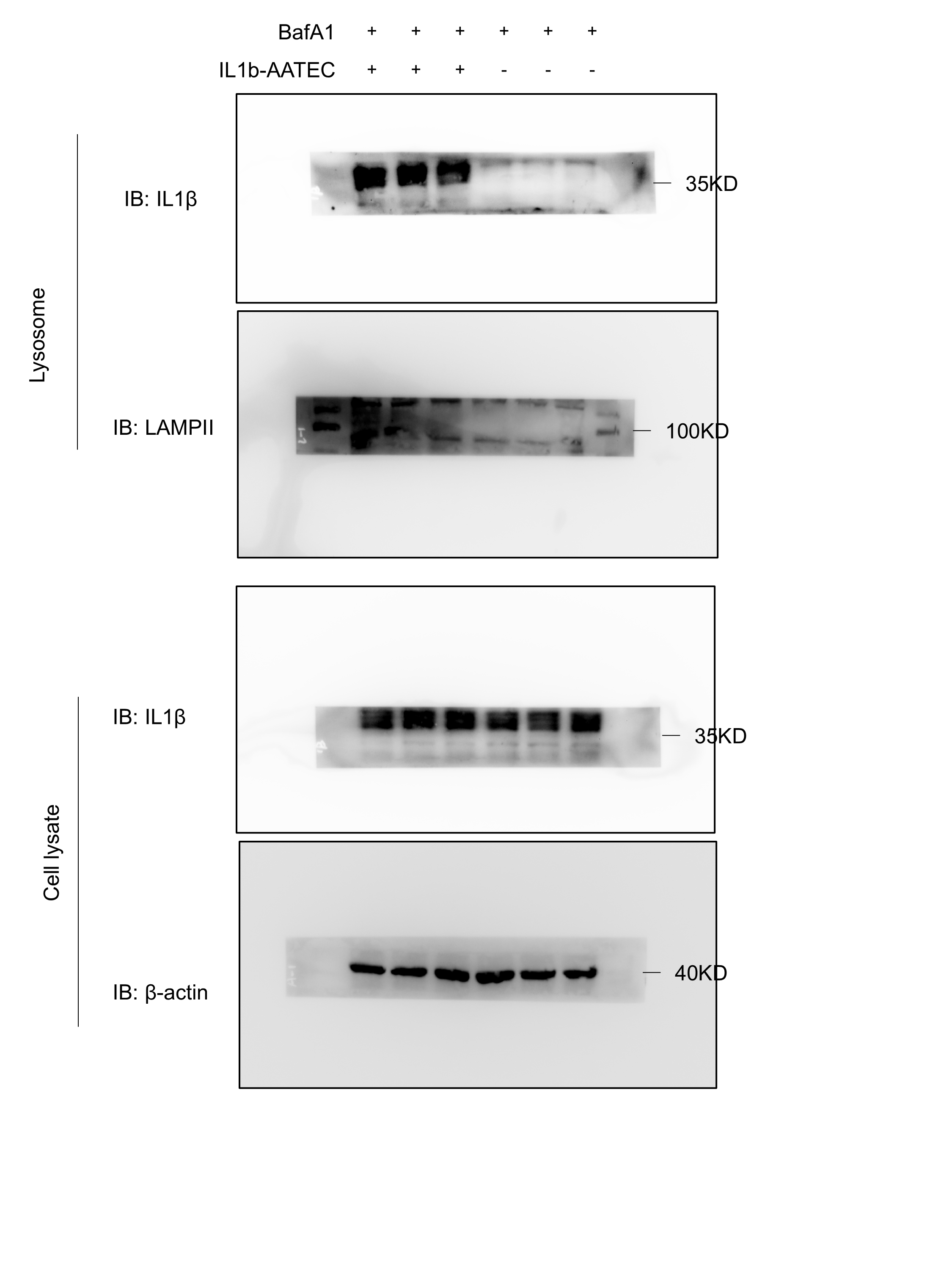

Supplement: Supplementary file 10 — Source data Fig. 4 [file 44321_2025_371_MOESM10_ESM.zip › Figure 4/Fig. 4n/Fig. 4n Summary plus label.tif]

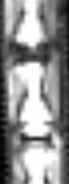

Supplement: Supplementary file 11 — Source data Fig. 5 [file 44321_2025_371_MOESM11_ESM.zip › Figure 5/Fig. 5h/Fig. 5h MRI EV.tif]

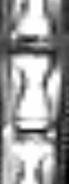

Supplement: Supplementary file 11 — Source data Fig. 5 [file 44321_2025_371_MOESM11_ESM.zip › Figure 5/Fig. 5h/Fig. 5h MRI I-EVTPD.tif]

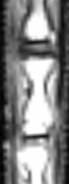

Supplement: Supplementary file 11 — Source data Fig. 5 [file 44321_2025_371_MOESM11_ESM.zip › Figure 5/Fig. 5h/Fig. 5h MRI T-EVTPD.tif]

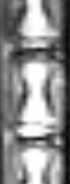

Supplement: Supplementary file 11 — Source data Fig. 5 [file 44321_2025_371_MOESM11_ESM.zip › Figure 5/Fig. 5h/Fig. 5h MRI TI-EVTPD.tif]

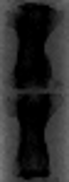

Supplement: Supplementary file 11 — Source data Fig. 5 [file 44321_2025_371_MOESM11_ESM.zip › Figure 5/Fig. 5h/Fig. 5h Xray EV.tif]

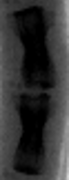

Supplement: Supplementary file 11 — Source data Fig. 5 [file 44321_2025_371_MOESM11_ESM.zip › Figure 5/Fig. 5h/Fig. 5h Xray I-EVTPD.tif]

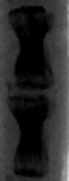

Supplement: Supplementary file 11 — Source data Fig. 5 [file 44321_2025_371_MOESM11_ESM.zip › Figure 5/Fig. 5h/Fig. 5h Xray T-EVTPD.tif]

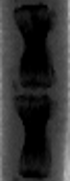

Supplement: Supplementary file 11 — Source data Fig. 5 [file 44321_2025_371_MOESM11_ESM.zip › Figure 5/Fig. 5h/Fig. 5h Xray TI-EVTPD.tif]

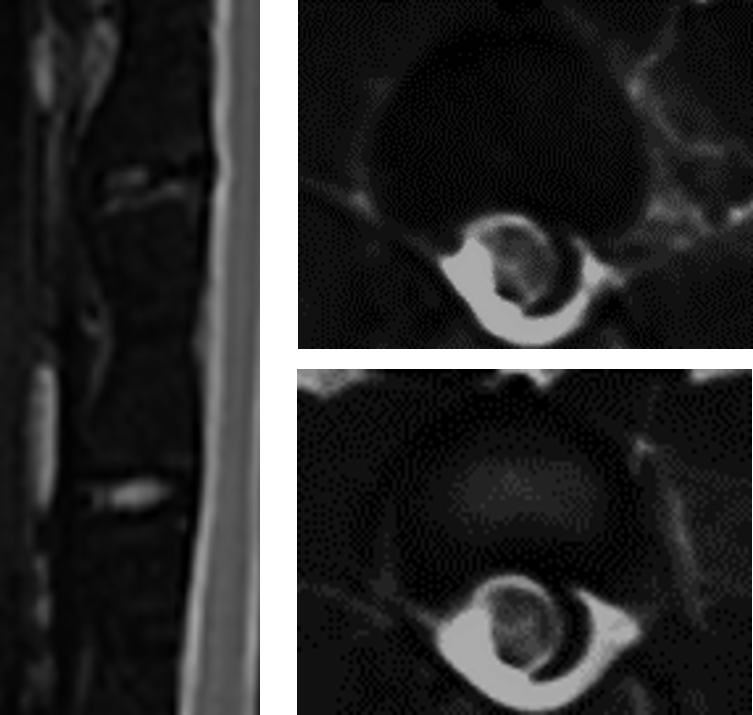

Supplement: Supplementary file 12 — Source data Fig. 6 [file 44321_2025_371_MOESM12_ESM.zip › Figure 6/Fig. 6b/Fig. 6b EVTPD 14d.tif]

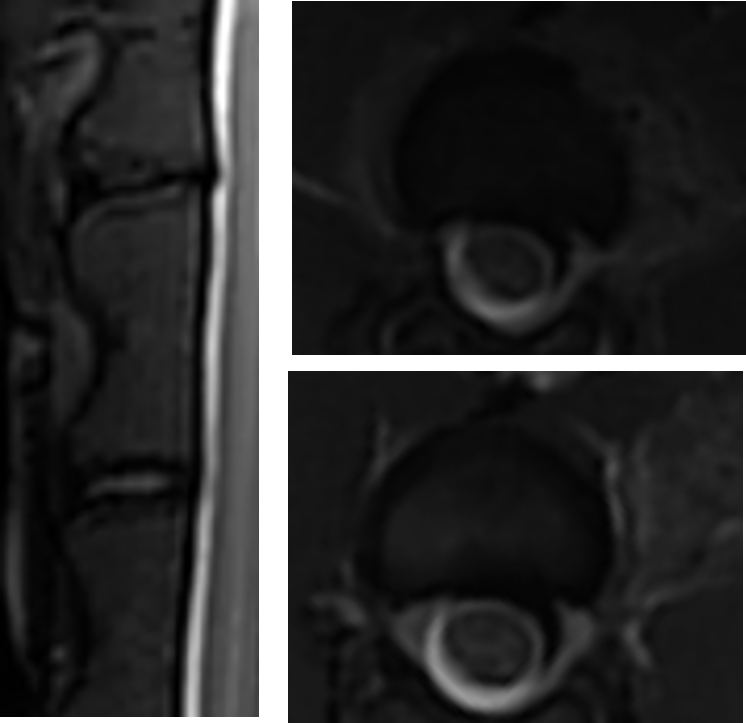

Supplement: Supplementary file 12 — Source data Fig. 6 [file 44321_2025_371_MOESM12_ESM.zip › Figure 6/Fig. 6b/Fig. 6b EVTPD 28d.tif]
