# Supplementary figures and images for: Extracellular vesicle-based targeted protein degradation platform for multiple extracellular proteins (part 2 of 2)
Source: EMBO Mol Med. 2026 Jan 12;18(2):759–94. doi: 10.1038/s44321-025-00371-8 (PMC12905291; doi:10.1038/s44321-025-00371-8)

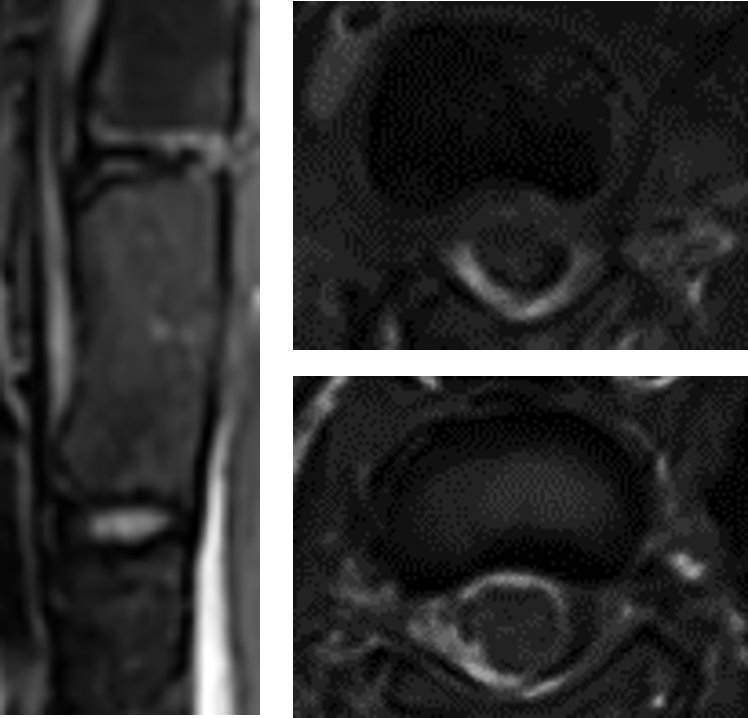

Supplement: Supplementary file 12 — Source data Fig. 6 [file 44321_2025_371_MOESM12_ESM.zip › Figure 6/Fig. 6b/Fig. 6b TI-EVTPD 14d.tif]

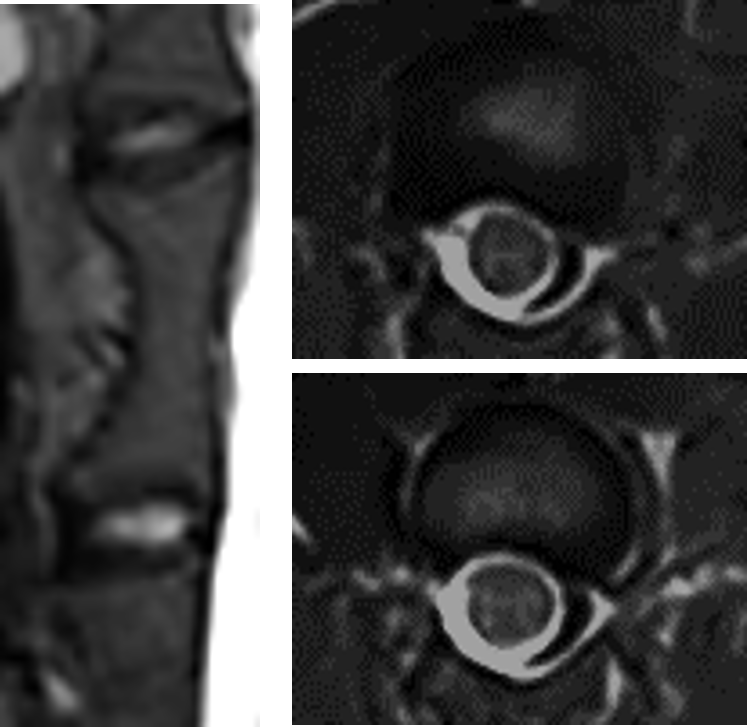

Supplement: Supplementary file 12 — Source data Fig. 6 [file 44321_2025_371_MOESM12_ESM.zip › Figure 6/Fig. 6b/Fig. 6b TI-EVTPD 28d.tif]

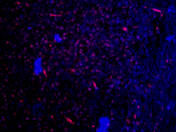

Supplement: Supplementary file 12 — Source data Fig. 6 [file 44321_2025_371_MOESM12_ESM.zip › Figure 6/Fig. 6d/Fig. 6d AF EVTPD.tif]

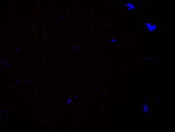

Supplement: Supplementary file 12 — Source data Fig. 6 [file 44321_2025_371_MOESM12_ESM.zip › Figure 6/Fig. 6d/Fig. 6d AF sham.tif]

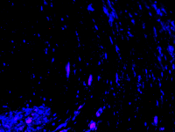

Supplement: Supplementary file 12 — Source data Fig. 6 [file 44321_2025_371_MOESM12_ESM.zip › Figure 6/Fig. 6d/Fig. 6d AF TI-EVTPD.tif]

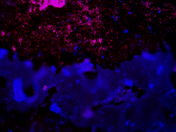

Supplement: Supplementary file 12 — Source data Fig. 6 [file 44321_2025_371_MOESM12_ESM.zip › Figure 6/Fig. 6d/Fig. 6d CEP EVTPD.tif]

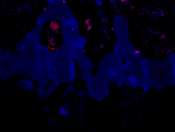

Supplement: Supplementary file 12 — Source data Fig. 6 [file 44321_2025_371_MOESM12_ESM.zip › Figure 6/Fig. 6d/Fig. 6d CEP sham.tif]

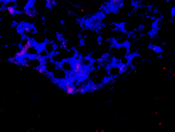

Supplement: Supplementary file 12 — Source data Fig. 6 [file 44321_2025_371_MOESM12_ESM.zip › Figure 6/Fig. 6d/Fig. 6d CEP TI-EVTPD.tif]

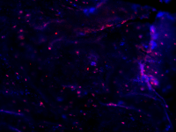

Supplement: Supplementary file 12 — Source data Fig. 6 [file 44321_2025_371_MOESM12_ESM.zip › Figure 6/Fig. 6d/Fig. 6d NP EVTPD.tif]

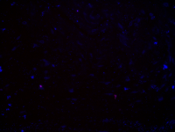

Supplement: Supplementary file 12 — Source data Fig. 6 [file 44321_2025_371_MOESM12_ESM.zip › Figure 6/Fig. 6d/Fig. 6d NP sham.tif]

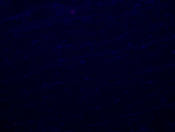

Supplement: Supplementary file 12 — Source data Fig. 6 [file 44321_2025_371_MOESM12_ESM.zip › Figure 6/Fig. 6d/Fig. 6d NP TI-EVTPD.tif]

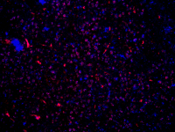

Supplement: Supplementary file 12 — Source data Fig. 6 [file 44321_2025_371_MOESM12_ESM.zip › Figure 6/Fig. 6e/Fig. 6e AF EVTPD.tif]

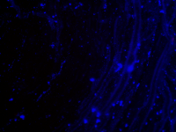

Supplement: Supplementary file 12 — Source data Fig. 6 [file 44321_2025_371_MOESM12_ESM.zip › Figure 6/Fig. 6e/Fig. 6e AF sham.tif]

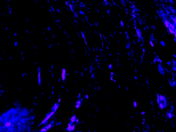

Supplement: Supplementary file 12 — Source data Fig. 6 [file 44321_2025_371_MOESM12_ESM.zip › Figure 6/Fig. 6e/Fig. 6e AF TI-EVTPD.tif]

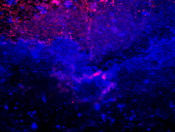

Supplement: Supplementary file 12 — Source data Fig. 6 [file 44321_2025_371_MOESM12_ESM.zip › Figure 6/Fig. 6e/Fig. 6e CEP EVTPD.tif]

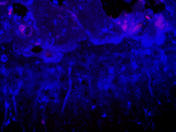

Supplement: Supplementary file 12 — Source data Fig. 6 [file 44321_2025_371_MOESM12_ESM.zip › Figure 6/Fig. 6e/Fig. 6e CEP sham.tif]

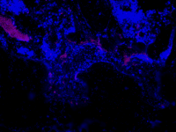

Supplement: Supplementary file 12 — Source data Fig. 6 [file 44321_2025_371_MOESM12_ESM.zip › Figure 6/Fig. 6e/Fig. 6e CEP TI-EVTPD.tif]

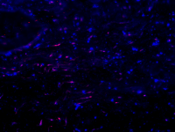

Supplement: Supplementary file 12 — Source data Fig. 6 [file 44321_2025_371_MOESM12_ESM.zip › Figure 6/Fig. 6e/Fig. 6e NP EVTPD.tif]

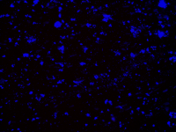

Supplement: Supplementary file 12 — Source data Fig. 6 [file 44321_2025_371_MOESM12_ESM.zip › Figure 6/Fig. 6e/Fig. 6e NP sham.tif]

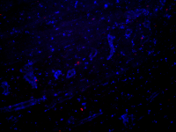

Supplement: Supplementary file 12 — Source data Fig. 6 [file 44321_2025_371_MOESM12_ESM.zip › Figure 6/Fig. 6e/Fig. 6e NP TI-EVTPD.tif]

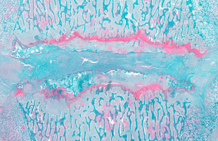

Supplement: Supplementary file 12 — Source data Fig. 6 [file 44321_2025_371_MOESM12_ESM.zip › Figure 6/Fig. 6g/Fig. 6g EVTPD.tif]

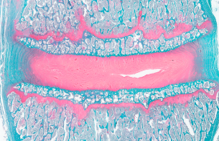

Supplement: Supplementary file 12 — Source data Fig. 6 [file 44321_2025_371_MOESM12_ESM.zip › Figure 6/Fig. 6g/Fig. 6g sham.tif]

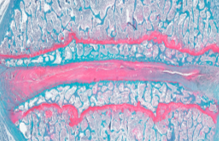

Supplement: Supplementary file 12 — Source data Fig. 6 [file 44321_2025_371_MOESM12_ESM.zip › Figure 6/Fig. 6g/Fig. 6g TI-EVTPD.tif]

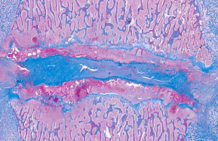

Supplement: Supplementary file 12 — Source data Fig. 6 [file 44321_2025_371_MOESM12_ESM.zip › Figure 6/Fig. 6h/Fig. 6h EV.tif]

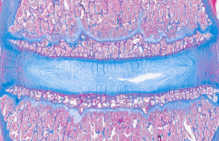

Supplement: Supplementary file 12 — Source data Fig. 6 [file 44321_2025_371_MOESM12_ESM.zip › Figure 6/Fig. 6h/Fig. 6h sham.tif]

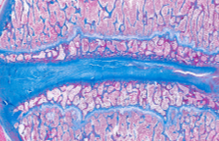

Supplement: Supplementary file 12 — Source data Fig. 6 [file 44321_2025_371_MOESM12_ESM.zip › Figure 6/Fig. 6h/Fig. 6h TI-EVTPD.tif]
