# Supplementary material for: Extracellular vesicle-based targeted protein degradation platform for multiple extracellular proteins
Source: EMBO Mol Med. 2026 Jan 12;18(2):759–94. doi: 10.1038/s44321-025-00371-8 (PMC12905291; doi:10.1038/s44321-025-00371-8)
Supplement: Supplementary file 13 — Expanded View Figures [file 44321_2025_371_MOESM13_ESM.pdf]

Expanded View Figures

Figure EV1. The degradation of extracellular vesicles occurs through an autophagy-dependent lysosomal pathway.

(**A**) Representative TEM image of HEK293T-derived EGFP-EVs (scale bar: 100 nm). (**B**) Nanoparticle tracking analysis (NTA) of EGFP-EVs. (**C**, **D**) Immunoblot assay analysis of EVs markers and EGFP expression in EGFP-EVs and cell lysates. (**E**) EGFP-EVs were added to NPCs for an incubation of 12 h. Then, free EGFP-EVs were washed out and replaced by fresh medium. Degradation of EGFP-EV in NPCs at 0, 3, 6, 12, and 24 h were analyzed by western blot ($n = 3$). (**F–H**) Analysis of EGFP level in DMSO, MG132 and BafA1 groups NPCs co-incubated with EGFP-EVs derived from HEK293T (**F**), HeLa (**G**) and MSC (**H**) cells for 12 h (uptake) and wash out for 12 h (degradation). (**I–L**) Analysis of EGFP level in DMSO, MG132 and BafA1 groups HeLa cells co-incubated with EGFP-EVs derived from HEK293T (**I**), HeLa (**J**) and MSC (**K**) cells for 12 h (uptake) and wash out for 12 h (degradation) ($n = 3$). (**M–O**) Analysis of EGFP level of EGFP in DMSO, 3-MA and ML282 groups NPCs co-incubated with EGFP-EVs derived from HEK293T (**M**), HeLa (**N**) and MSC (**O**) cells for 12 h (uptake) and wash out for 12 h (degradation). (**P–S**) Analysis of EGFP level in DMSO, 3-MA and ML282 groups HeLa cells co-incubated with EGFP-EVs derived from HEK293T (**P**), HeLa (**Q**) and MSC (**R**) cells for 12 h (uptake) and wash out for 12 h (degradation) ($n = 3$); (**T**) NPCs were treated with EGFP-EVs for 12 h. After incubation, free EGFP-EVs were washed out and replaced by fresh medium with 50 nM BafA1 for 12 h. Co-localization of EGFP and LAMPII was analyzed by immunofluorescence (scale bar: 10 μm) ($n = 6$). (**U**) NPCs were treated with EGFP-EVs for 12 h. After incubation, free EGFP-EVs were washed out and replaced by fresh medium with 50 nM BafA1 for 12 h. Lysosome enrichment level of EGFP in NPCs in control, 3-MA and ML282 groups were analyzed by western blot ($n = 4$). (**V**) Analysis of lysosome enrichment level of EGFP in shScramble and shATG7-treated NPCs ($n = 3$). Data were analyzed by unpaired two-tailed *t* tests (**L**, **S–V**). Data were shown as mean ± SD. Each *n* in (**L**, **S–V**) is biological independent samples. The *P* values are labeled in the figure.

© The Author(s)

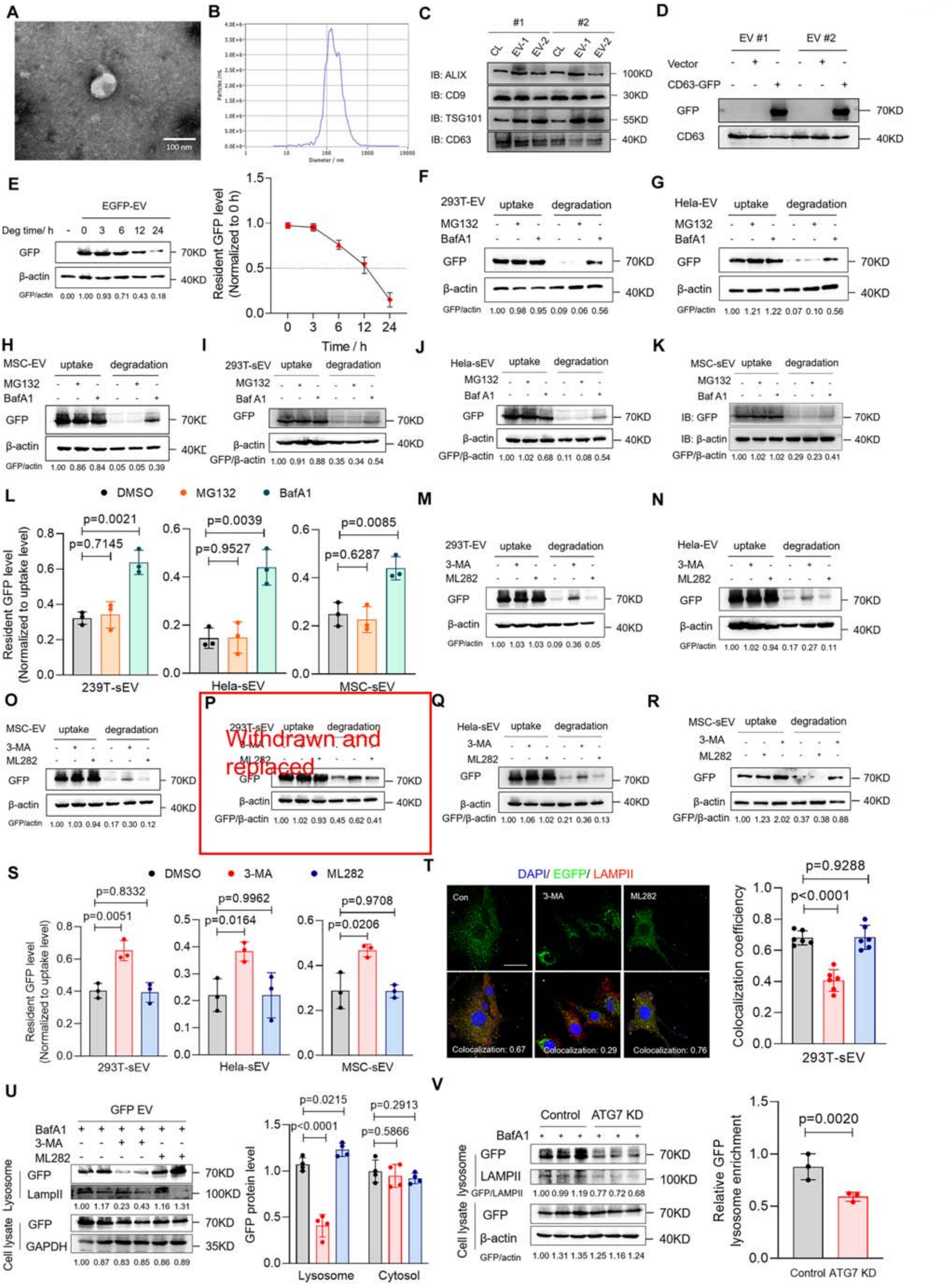

A
100 nm
B
Particles / mL
Diameter / nm
C
#1
#2
CL
EV-1
EV-2
IB: ALIX
IB: CD9
IB: TSG101
IB: CD63
100KD
30KD
55KD
40KD
D
EV #1
EV #2
Vector
CD63-GFP
GFP
CD63
70KD
40KD
E
EGFP-EV
Deg time/ h
0 3 6 12 24
GFP
β-actin
GFP/actin 0.00 1.00 0.93 0.71 0.43 0.18
Resident GFP level (Normalized to 0 h)
Time / h
F
293T-EV
uptake
degradation
MG132
BafA1
GFP/actin 1.00 0.98 0.95 0.09 0.06 0.56
G
Hela-EV
GFP/actin 1.00 1.21 1.22 0.07 0.10 0.56
H
MSC-EV
GFP/actin 1.00 0.86 0.84 0.05 0.05 0.39
I
293T-sEV
Baf A1
GFP/β-actin 1.00 0.91 0.88 0.35 0.34 0.54
J
Hela-sEV
GFP/β-actin 1.00 1.02 0.68 0.11 0.08 0.54
K
MSC-sEV
IB: GFP
IB: β-actin
GFP/β-actin 1.00 1.02 1.02 0.29 0.23 0.41
L
DMSO
MG132
BafA1
Resident GFP level (Normalized to uptake level)
p=0.0021
p=0.7145
p=0.0039
p=0.9527
p=0.0085
p=0.6287
239T-sEV
Hela-sEV
MSC-sEV
M
293T-EV
3-MA
ML282
GFP/actin 1.00 1.03 1.03 0.09 0.36 0.05
N
Hela-EV
GFP/actin 1.00 1.02 0.94 0.17 0.27 0.11
O
MSC-EV
GFP/actin 1.00 1.03 0.94 0.17 0.30 0.12
P
Withdrawn and replaced
GFP/β-actin 1.00 1.02 0.93 0.45 0.62 0.41
Q
Hela-sEV
GFP/β-actin 1.00 1.06 1.02 0.21 0.36 0.13
R
MSC-sEV
GFP/β-actin 1.00 1.23 2.02 0.37 0.38 0.88
S
DMSO
3-MA
ML282
p=0.8332
p=0.0051
p=0.9962
p=0.0164
p=0.9708
p=0.0206
293T-sEV
Hela-sEV
MSC-sEV
T
DAPI/ EGFP/ LAMPII
Con
3-MA
ML282
Colocalization: 0.67
Colocalization: 0.29
Colocalization: 0.76
Colocalization coefficiency
p=0.9288
p<0.0001
293T-sEV
U
GFP EV
BafA1
3-MA
ML282
Lysosome
Cell lysate
GFP
LampII
GAPDH
1.00 1.17 0.23 0.43 1.16 1.31
1.00 0.87 0.83 0.85 0.86 0.89
70KD
100KD
35KD
GFP protein level
p=0.0215
p<0.0001
p=0.2913
p=0.5866
Lysosome
Cytosol
V
Control
ATG7 KD
LAMPII
GFP/LAMPII 1.00 0.99 1.19 0.77 0.72 0.68
GFP/actin 1.00 1.31 1.35 1.25 1.16 1.24
Relative GFP lysosome enrichment
p=0.0020
Control ATG7 KD

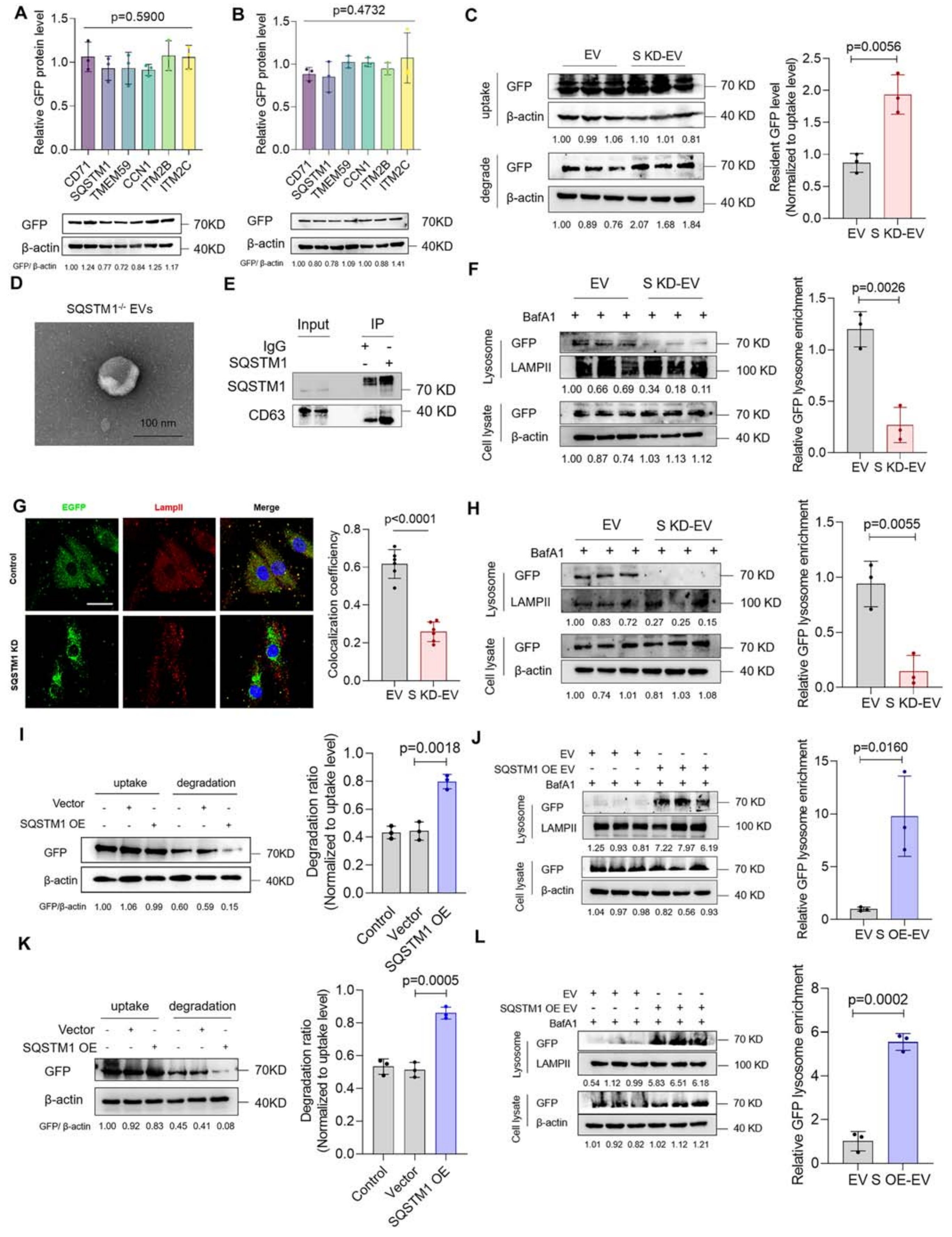
A
Relative GFP protein level
p=0.5900
CD71
SQSTM1
TMEM59
CCN1
ITM2B
ITM2C
GFP
β-actin
70KD
40KD
GFP/ β-actin 1.00 1.24 0.77 0.72 0.84 1.25 1.17
B
Relative GFP protein level
p=0.4732
GFP/ β-actin 1.00 0.80 0.78 1.09 1.00 0.88 1.41
C
EV
S KD-EV
uptake
degrade
70 KD
40 KD
1.00 0.99 1.06 1.10 1.01 0.81
1.00 0.89 0.76 2.07 1.68 1.84
Resident GFP level (Normalized to uptake level)
p=0.0056
D
SQSTM1-/- EVs
100 nm
E
Input
IP
IgG
SQSTM1
CD63
F
BafA1
Lysosome
Cell lysate
LAMPII
100 KD
1.00 0.66 0.69 0.34 0.18 0.11
1.00 0.87 0.74 1.03 1.13 1.12
Relative GFP lysosome enrichment
p=0.0026
G
EGFP
LampII
Merge
Control
SQSTM1 KD
Colocalization coefficiency
p<0.0001
H
1.00 0.83 0.72 0.27 0.25 0.15
1.00 0.74 1.01 0.81 1.03 1.08
p=0.0055
I
uptake
degradation
Vector
SQSTM1 OE
GFP/β-actin 1.00 1.06 0.99 0.60 0.59 0.15
Degradation ratio (Normalized to uptake level)
p=0.0018
Control
J
SQSTM1 OE EV
1.25 0.93 0.81 7.22 7.97 6.19
1.04 0.97 0.98 0.82 0.56 0.93
p=0.0160
EV S OE-EV
K
GFP/ β-actin 1.00 0.92 0.83 0.45 0.41 0.08
p=0.0005
L
0.54 1.12 0.99 5.83 6.51 6.18
1.01 0.92 0.82 1.02 1.12 1.21
p=0.0002

Figure EV2. SQSTM1 mediates extracellular vesicles entry into lysosome-dependent degradation pathway.

(**A**) The uptake levels of specific antibody-blocking EVs (IgG, CD71, SQSTM1, TMEM59, CCN1, ITM2B, ITM2C) were detected after incubation for 12 h. IgG control was used to normalize the grayscale values of each group. Data were analyzed by one-way ANOVA ($n = 3$). (**B**) The uptake levels of specific gene-knockdown EVs (Vector, CD71, SQSTM1, TMEM59, CCN1, ITM2B, ITM2C) were detected after incubation for 12 h. Vector control was used to normalize the grayscale values of each group. Data were analyzed by one-way ANOVA ($n = 3$). (**C**) Control EVs and SQSTM1 KD EVs were incubated with NPCs for 12 h. Uptake level was analyzed at this time while degradation level was detected following wash out and another 12 h incubation ($n = 3$). (**D**) IEM assay to detect the expression of SQSTM1 on HEK-293T derived EVs (scale bar: 100 nm). (**E**) Anti-SQSTM1 antibody was used to pull down HEK-293T derived EVs. IgG was used as negative control. SQSTM1 as well as EV marker-CD63 were detected by western blot. (**F**) Control or SQSTM1 KD EVs were added to NPCs and co-incubated for 12 h. Then, free EVs were wash out and replaced by free mediun containing 50 nM BafA1 for 12 h. After incubation, lysosome enrichment method was used to detect the sorting efficiency of control and SQSTM1 knockdown EVs into lysosomes in NPCs ($n = 3$). (**G**) Control or SQSTM1 KD EVs were added to NPCs and co-incubated for 12 h. Then, free EVs were washed out and replaced by free mediun containing 50 nM BafA1 for 12 h. After incubation, immunofluorescence assay was used to detect the co-localization of EGFP with LampII (scale bar: 10 μm) ($n = 6$); (**H**) Control or SQSTM1 KD EVs were added to HeLa cells and co-incubated for 12 h. Then, free EVs were washed out and replaced by free mediun containing 50 nM BafA1 for 12 h. After incubation, lysosome enrichment method was used to detect the sorting efficiency of control and SQSTM1 knockdown EVs into lysosomes in HeLa cells ($n = 3$); (**I**, **J**) Analysis of degradation rate (**I**) and lysosomal sorting efficiency (**J**) of control and SQSTM1 overexpression EVs in NPCs ($n = 3$); (**K**, **L**) Analysis of degradation rate (**K**) and lysosomal sorting efficiency (l) of control and SQSTM1 overexpression EVs in HeLa cells ($n = 3$). Data were analyzed by one-way ANOVA (**A**, **B**) unpaired two-tailed *t* tests (**C**, **F**, **G**, **H**, **I**, **J**, **K**, **L**). Data were shown as mean ± SD. Each *n* in (**A**, **B**, **C**, **F**, **G**, **H**, **I**, **J**, **K**, **L**) is biological independent samples. The *P* values are labeled in the figure.

 © The Author(s)

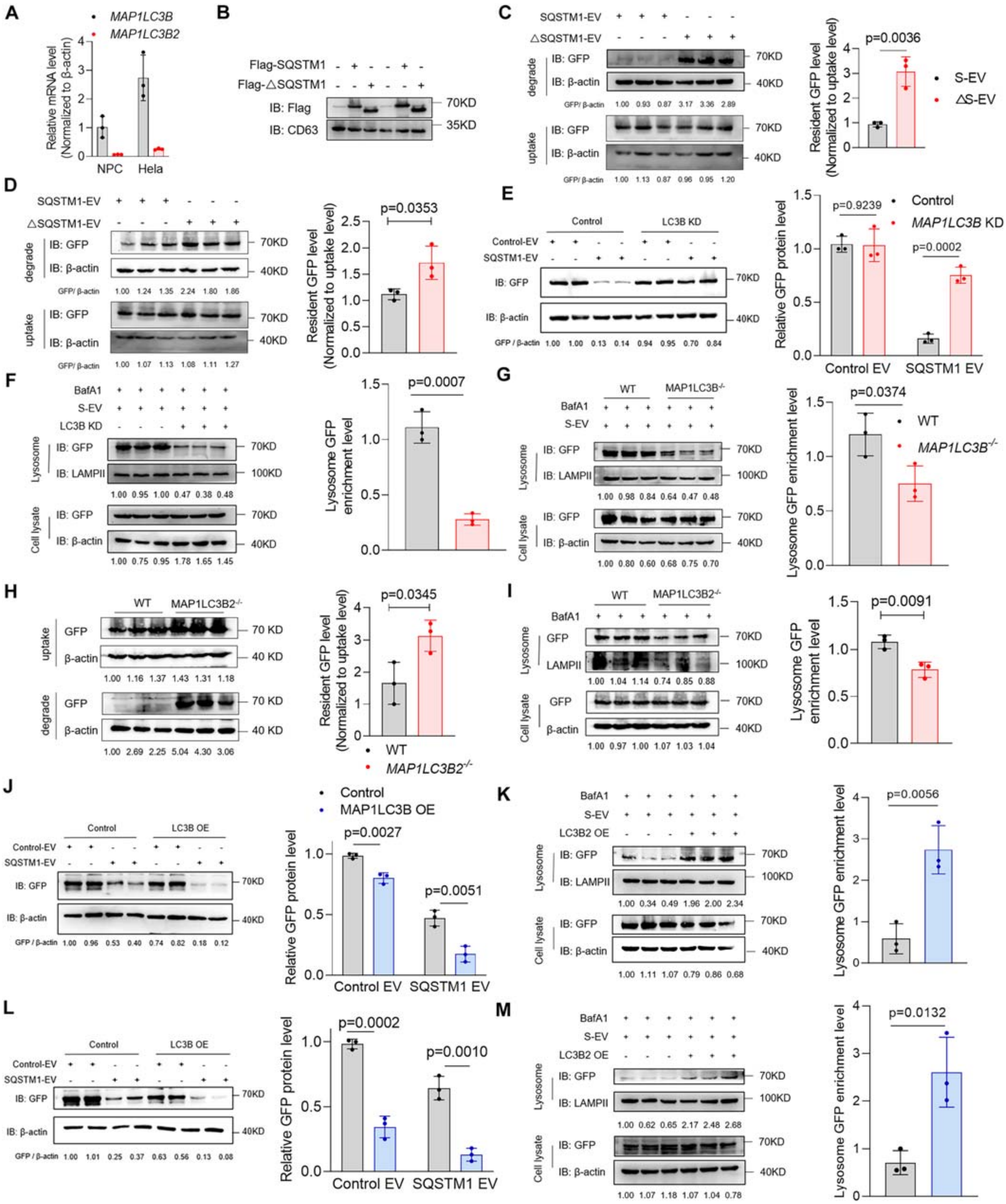
A
MAP1LC3B
MAP1LC3B2
Relative mRNA level
(Normalized to β-actin)
NPC
Hela
B
Flag-SQSTM1
Flag-△SQSTM1
IB: Flag
IB: CD63
70KD
35KD
C
SQSTM1-EV
△SQSTM1-EV
degrade
uptake
IB: GFP
IB: β-actin
GFP/ β-actin
1.00 0.93 0.87 3.17 3.36 2.89
1.00 1.13 0.87 0.96 0.95 1.20
70KD
40KD
p=0.0036
Resident GFP level
(Normalized to uptake level)
S-EV
ΔS-EV
D
SQSTM1-EV
△SQSTM1-EV
degrade
uptake
IB: GFP
IB: β-actin
GFP/ β-actin
1.00 1.24 1.35 2.24 1.80 1.86
1.00 1.07 1.13 1.08 1.11 1.27
p=0.0353
Resident GFP level
(Normalized to uptake level)
E
Control
LC3B KD
Control-EV
SQSTM1-EV
IB: GFP
IB: β-actin
GFP / β-actin 1.00 1.00 0.13 0.14 0.94 0.95 0.70 0.84
p=0.9239
p=0.0002
Control
MAP1LC3B KD
Relative GFP protein level
Control EV
SQSTM1 EV
F
BafA1
S-EV
LC3B KD
Lysosome
Cell lysate
IB: GFP
IB: LAMPII
IB: β-actin
1.00 0.95 1.00 0.47 0.38 0.48
1.00 0.75 0.95 1.78 1.65 1.45
70KD
100KD
40KD
p=0.0007
Lysosome GFP
enrichment level
G
WT
MAP1LC3B-/-
BafA1
S-EV
Lysosome
Cell lysate
IB: GFP
IB: LAMPII
IB: β-actin
1.00 0.98 0.84 0.64 0.47 0.48
1.00 0.80 0.60 0.68 0.75 0.70
p=0.0374
WT
MAP1LC3B-/-
Lysosome GFP enrichment level
H
WT
MAP1LC3B2-/-
uptake
degrade
GFP
β-actin
1.00 1.16 1.37 1.43 1.31 1.18
1.00 2.69 2.25 5.04 4.30 3.06
70 KD
40 KD
p=0.0345
Resident GFP level
(Normalized to uptake level)
WT
MAP1LC3B2-/-
I
WT
MAP1LC3B2-/-
BafA1
Lysosome
Cell lysate
GFP
LAMPII
β-actin
1.00 1.04 1.14 0.74 0.85 0.88
1.00 0.97 1.00 1.07 1.03 1.04
p=0.0091
Lysosome GFP
enrichment level
J
Control
LC3B OE
Control-EV
SQSTM1-EV
IB: GFP
IB: β-actin
GFP / β-actin 1.00 0.96 0.53 0.40 0.74 0.82 0.18 0.12
Control
MAP1LC3B OE
p=0.0027
p=0.0051
Relative GFP protein level
Control EV
SQSTM1 EV
K
BafA1
S-EV
LC3B2 OE
Lysosome
Cell lysate
IB: GFP
IB: LAMPII
IB: β-actin
1.00 0.34 0.49 1.96 2.00 2.34
1.00 1.11 1.07 0.79 0.86 0.68
p=0.0056
Lysosome GFP enrichment level
L
Control
LC3B OE
Control-EV
SQSTM1-EV
IB: GFP
IB: β-actin
GFP / β-actin 1.00 1.01 0.25 0.37 0.63 0.56 0.13 0.08
p=0.0002
p=0.0010
Relative GFP protein level
Control EV
SQSTM1 EV
M
BafA1
S-EV
LC3B2 OE
Lysosome
Cell lysate
IB: GFP
IB: LAMPII
IB: β-actin
1.00 0.62 0.65 2.17 2.48 2.68
1.00 1.07 1.18 1.07 1.04 0.78
p=0.0132
Lysosome GFP enrichment level

Figure EV3. EVs engineered with SQSTM1 degradation signal can be efficiently sorted into lysosome and degraded via interacting with MAP1LC3B.

(**A**) The mRNA level of MAP1LC3B and MAP1LC3B2 in NPCs and HeLa cells were analyzed by Real-time PCR ($n = 3$). The expression of MAP1LC3B in NPCs was normalized as control. (**B**) The expression of Flag tagged SQSTM1 and Flag tagged truncated SQSTM1 in engineering EVs were detected by western blot. (**C**) SQSTM1 and its truncated form loaded EVs were incubated with NPCs for 12 h. Uptake level was analyzed at this time while degradation level was detected following wash out and another 12 h incubation ($n = 3$). (**D**) SQSTM1 and its truncated form loaded EVs were incubated with HeLa cells for 12 h. Uptake level was analyzed at this time while degradation level was detected following wash out and another 12 h incubation ($n = 3$). (**E**) Degradation level of control and SQSTM1-EVs in control or LC3B KD NPCs were detected after 12 h incubation following with washed out and another 12 h incubation in fresh medium ($n = 3$). (**F**) SQSTM1-EVs were added to control or LC3B KD NPCs for incubation of 12 h followed with wash out and incubation in fresh medium containing 50 nM BafA1 for 12 h. Lysosomal enrichment level were analyzed by lysosomal isolation and western blot ($n = 3$). (**G**) Lysosomal enrichment level of SQSTM1-EVs in wild-type and MAP1LC3B KO HeLa cells were analyzed by lysosomal isolation and western blot ($n = 3$). (**H**) The uptake and degradation level of SQSTM1 EVs in wild-type and MAP1LC3B2 KO HeLa cells were analyzed by western blot ($n = 3$). (**I**) Detection of lysosomal enrichment efficiency of SQSTM1-EV in wild-type and MAP1LC3B2 knockout HeLa cell lines ($n = 3$). (**J**, **K**) Detection of degradation level (**J**) and lysosomal enrichment efficiency (**K**) of SQSTM1-EV in control and MAP1LC3B overexpression NPC ($n = 3$). (**L**, **M**) Detection of degradation level (**L**) and lysosomal enrichment efficiency (m) of SQSTM1-EV in control and MAP1LC3B overexpression HeLa cells ($n = 3$). Data were analyzed by unpaired two-tailed *t* tests (**A**, **C**–**M**). Data were shown as mean ± SD. Each *n* in (**A**, **C**–**M**) is biological independent samples. The *P* values are labeled in the figure.

 © The Author(s)

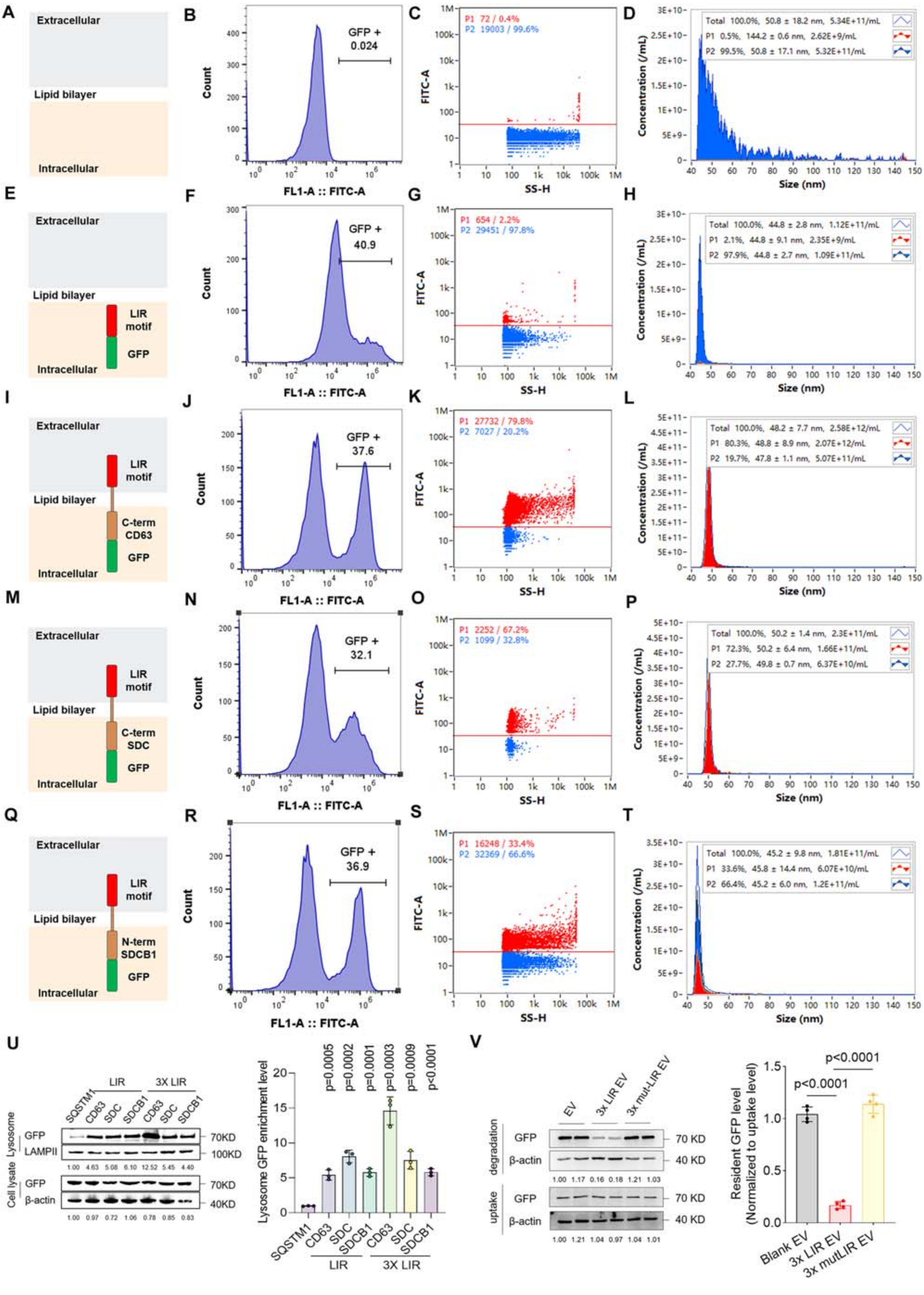
A
Extracellular
Lipid bilayer
Intracellular
B
GFP +
0.024
Count
FL1-A :: FITC-A
C
P1 72 / 0.4%
P2 19003 / 99.6%
FITC-A
SS-H
D
Total 100.0%, 50.8 ± 18.2 nm, 5.34E+11/mL
P1 0.5%, 144.2 ± 0.6 nm, 2.62E+9/mL
P2 99.5%, 50.8 ± 17.1 nm, 5.32E+11/mL
Concentration (/mL)
Size (nm)
E
LIR motif
GFP
F
GFP +
40.9
G
P1 654 / 2.2%
P2 29451 / 97.8%
H
Total 100.0%, 44.8 ± 2.8 nm, 1.12E+11/mL
P1 2.1%, 44.8 ± 9.1 nm, 2.35E+9/mL
P2 97.9%, 44.8 ± 2.7 nm, 1.09E+11/mL
I
C-term CD63
J
GFP +
37.6
K
P1 27732 / 79.8%
P2 7027 / 20.2%
L
Total 100.0%, 48.2 ± 7.7 nm, 2.58E+12/mL
P1 80.3%, 48.8 ± 8.9 nm, 2.07E+12/mL
P2 19.7%, 47.8 ± 1.1 nm, 5.07E+11/mL
M
C-term SDC
N
GFP +
32.1
O
P1 2252 / 67.2%
P2 1099 / 32.8%
P
Total 100.0%, 50.2 ± 1.4 nm, 2.3E+11/mL
P1 72.3%, 50.2 ± 6.4 nm, 1.66E+11/mL
P2 27.7%, 49.8 ± 0.7 nm, 6.37E+10/mL
Q
N-term SDCB1
R
GFP +
36.9
S
P1 16248 / 33.4%
P2 32369 / 66.6%
T
Total 100.0%, 45.2 ± 9.8 nm, 1.81E+11/mL
P1 33.6%, 45.8 ± 14.4 nm, 6.07E+10/mL
P2 66.4%, 45.2 ± 6.0 nm, 1.2E+11/mL
U
LIR
3X LIR
SQSTM1 CD63 SDC SDCB1 CD63 SDC SDCB1
Lysosome
GFP
LAMPII
70KD
100KD
1.00 4.63 5.08 6.10 12.52 5.45 4.40
Cell lysate
GFP
β-actin
70KD
40KD
1.00 0.97 0.72 1.06 0.78 0.85 0.83
Lysosome GFP enrichment level
p=0.0005
p=0.0002
p=0.0001
p=0.0003
p=0.0009
p<0.0001
V
EV
3x LIR EV
3x mut-LIR EV
degradation
GFP
β-actin
70 KD
40 KD
1.00 1.17 0.16 0.18 1.21 1.03
uptake
1.00 1.21 1.04 0.97 1.04 1.01
Resident GFP level (Normalized to uptake level)
p<0.0001
p<0.0001
Blank EV
3x LIR EV
3x mutLIR EV

Figure EV4. The loading efficiency of EV expression scaffolds.

(**A–D**) Control HEK293T (**A**) was analyzed by cytometry for GFP expression (**B**). HEK293T-derived EVs were analyzed by Nanoflow for GFP expression (**C**) as well as size distribution (**D**). (**E–H**) GFP-ILR motif transfected HEK293T (**E**) was analyzed by cytometry for GFP expression (**F**). Transfected HEK293T-derived EVs were analyzed by Nanoflow for GFP expression (**G**) as well as size distribution (**H**). (**I–L**) GFP-C term CD63-ILR motif transfected HEK293T (**I**) was analyzed by cytometry for GFP expression (**J**). Transfected HEK293T-derived EVs were analyzed by Nanoflow for GFP expression (**K**) as well as size distribution (**L**). (**M–P**) GFP-C term SDC-ILR motif transfected HEK293T (**M**) was analyzed by cytometry for GFP expression (**N**). Transfected HEK293T-derived EVs were analyzed by Nanoflow for GFP expression (**O**) as well as size distribution (**P**). (**Q–T**) GFP-N term SDCB1-ILR motif transfected HEK293T (**Q**) was analyzed by cytometry for GFP expression (**R**). Transfected HEK293T-derived EVs were analyzed by Nanoflow for GFP expression (**S**) as well as size distribution (**T**). (**U**) Different engineering EVs were added to NPCs for incubation of 12 h followed with wash out and incubation in fresh medium containing 50 nM BafA1 for 12 h. Lysosomal sorting efficiency of different EVs were detected by lysosome isolation and western blot ($n = 3$). (**V**) Representative immunoblot images and quantitative analysis of the resident GFP levels of blank, 3x LIR motifs and 3x L341V-mutated LIR motifs engineering EVs in HeLa cells ($n = 4$). Data were analyzed by unpaired two-tailed *t* tests (**U**, **V**). Data were shown as mean ± SD. Each *n* in (**U**, **V**) is biological independent samples. The *P* values are labeled in the figure.

 © The Author(s)

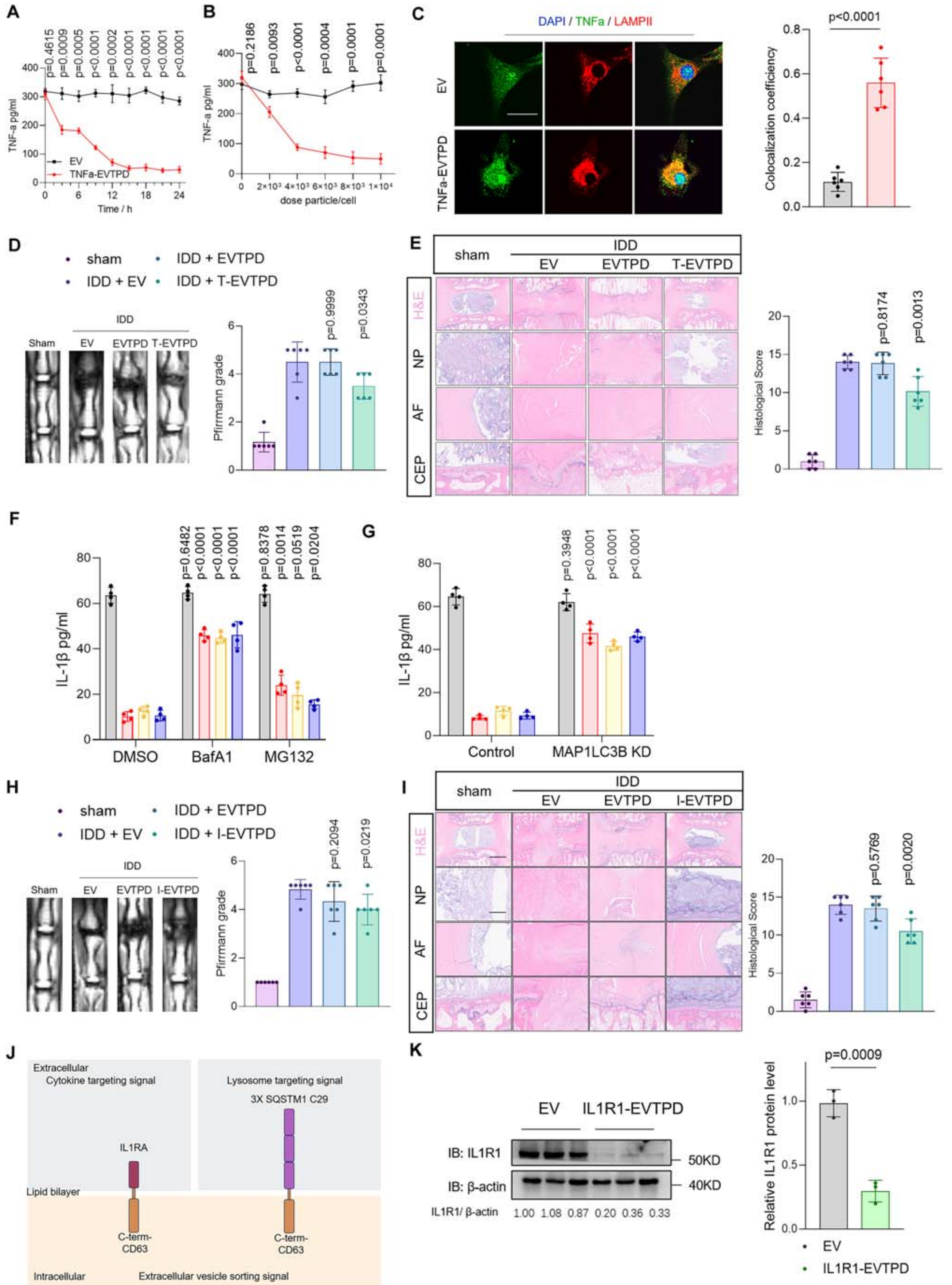

Figure EV5. EVs engineered with both degradation signal and cytokine-binding domains efficiently degrade TNF-α and IL-1β.

(**A**) The degradation level of TNF-α in the supernatant of NPCs by blank EVs or TNFα-EVTPD at 0,3,6,9,12,15,18,21,24 h were detected by EVTPD assays ($n = 3$). (**B**) The degradation rate of TNF-α in the supernatant of NPCs by blank EVs and TNFα- EVTPD at concentration of 0, 2, 4, 6, 8 and 10×10^3 per cell were detected by ELISA assays ($n = 3$); (**C**) Fluorescence co-localization analysis of TNF-α and LAMPII in NPCs after EV, TNFA- EVTPD treatment for 24 h with 50 nM BafA1 ($n = 6$). (**D**) MRI images of co7/8 and co8/9 as control. The Pfirrmann grades of sham, IDD + EV, IDD + EVTPD and IDD + rTNFα- EVTPD were analyzed based on the MRI images ($n = 6$). (**E**) H&E staining images including intire intervertebral disc, nucleus pulposus (NP), annulus fibrosus (AF), and cartilage endplates (CEP) and histological scores of intervertebral disc sham, IDD + EV, IDD + EVTPD and IDD + rTNFα-EVTPD groups (Scale bar: 50 µm and 5 µm). (**F**) ELISA was used to detect the degradation rate of IL1β by IL1β-targeting EVs loaded with or without degradation signal in the DMSO, BafA1 and MG132 pretreated groups ($n = 4$). (**G**) ELISA was used to detect the effect of MAP1LC3B knockdown on the IL1β-degradation ability of engineered EVs ($n = 4$). (**H**) MRI images of co7/8 and co8/9 as control. The Pfirrmann grades of sham, IDD + EV, IDD + EVTPD and IDD + rIL1β- EVTPD were analyzed based on the MRI images ($n = 6$). (**I**) H&E staining images including entire intervertebral disc, nucleus pulposus (NP), annulus fibrosus (AF), and cartilage endplates (CEP) and histological scores of intervertebral disc tissue of rats in sham, IDD + EV, IDD + EVTPD and IDD + rIL1β-EVTPD groups (scale bar: 50 µm and 5 µm). (**J**) Schematic diagram of EVs loaded with degradation signal and IL1R1 binding domain. (**K**) Protein level analysis of IL1R1 levels after blank EV or IL1R1-EVTPD treatment for 24 h ($n = 3$). Data were analyzed by unpaired two-tailed *t* tests (**A–I**, **K**). Data were shown as mean ± SD. Each *n* in (**D**, **E**, **H**, **I**) is an individual rat, each *n* in (**A–C**, **F**, **G**, **K**) is biological independent samples. The *P* values are labeled in the figure.

 © The Author(s)
