# Supplementary figures and images for: Author Correction: Extracellular vesicle-based targeted protein degradation platform for multiple extracellular proteins
Source: EMBO Mol Med. 2026 May 19;18(6):2514–7. doi: 10.1038/s44321-026-00439-z (PMC13269784; doi:10.1038/s44321-026-00439-z)

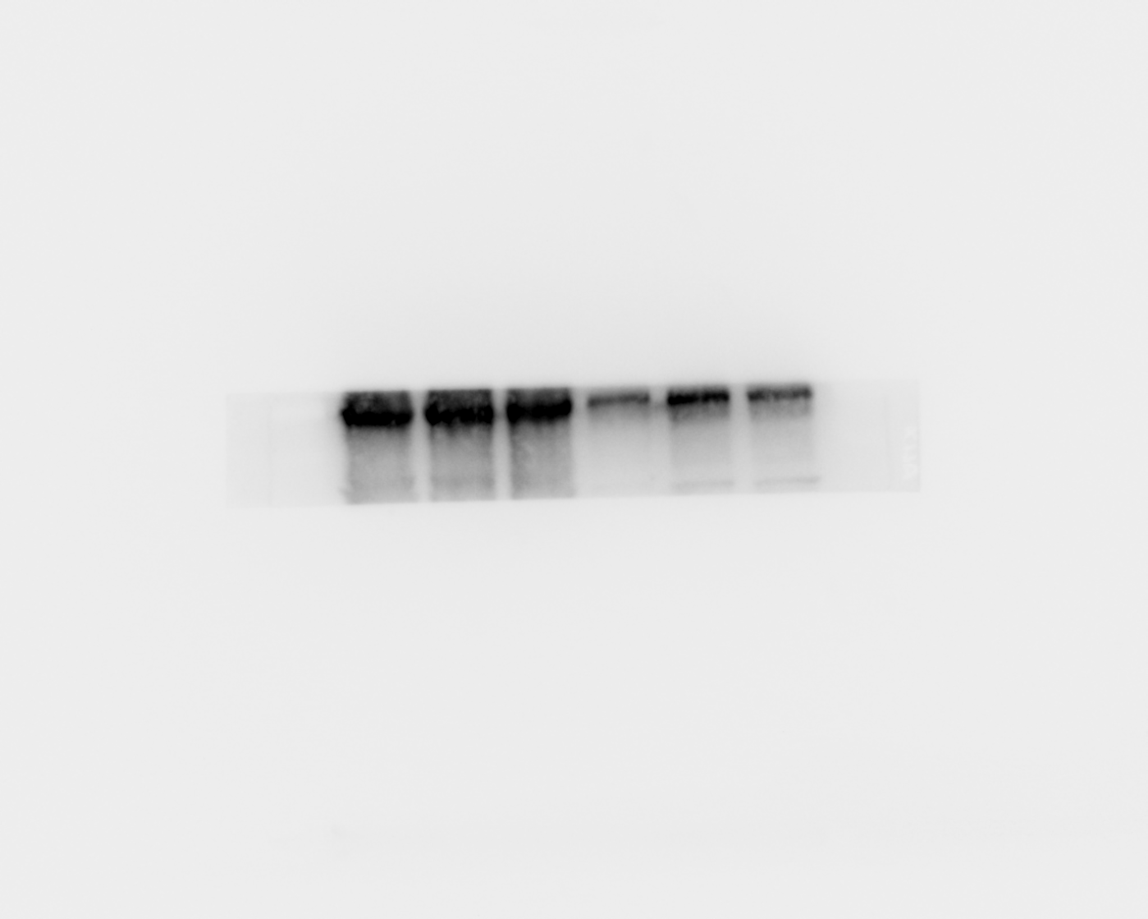

Supplement: Supplementary file 1 — Source Data [file 44321_2026_439_MOESM1_ESM.zip › Source Data/Original Figure EV1p GFP.tif]

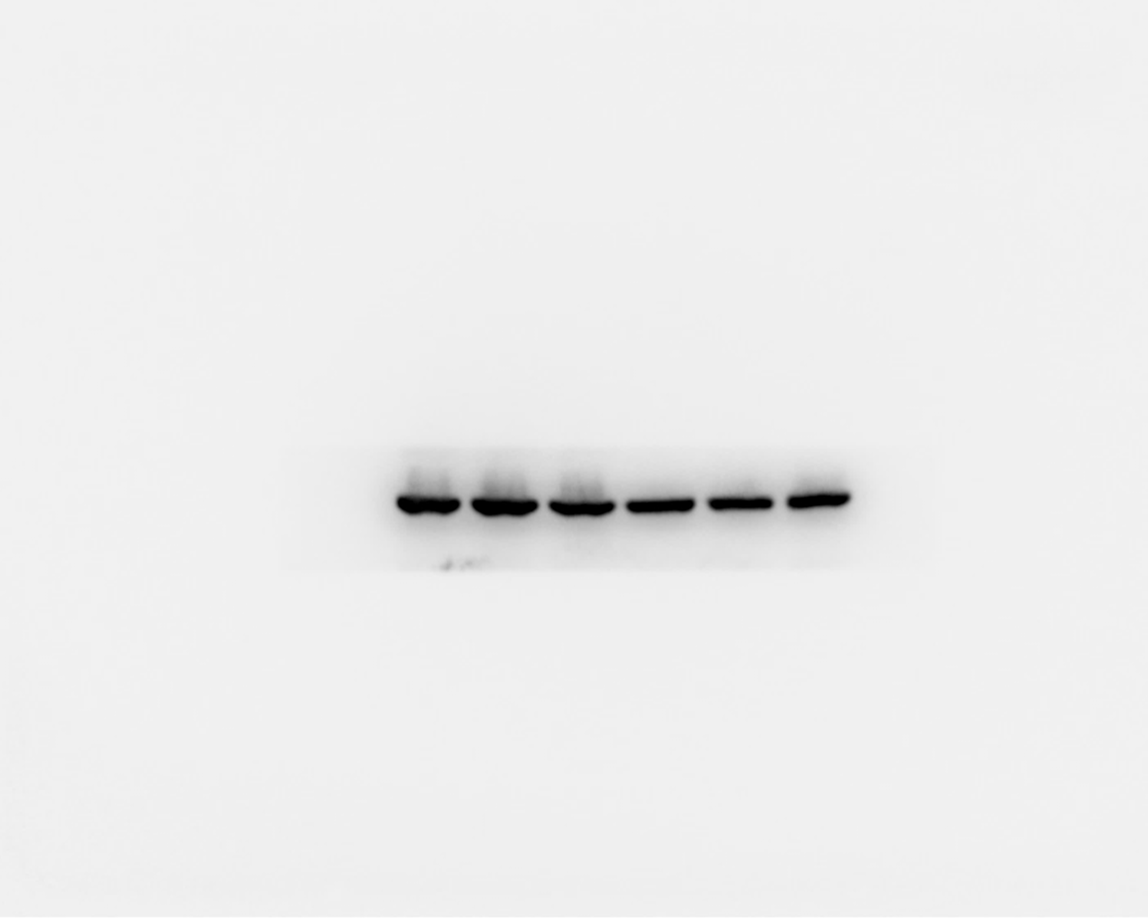

Supplement: Supplementary file 1 — Source Data [file 44321_2026_439_MOESM1_ESM.zip › Source Data/Original Figure EV1p actin.tif]

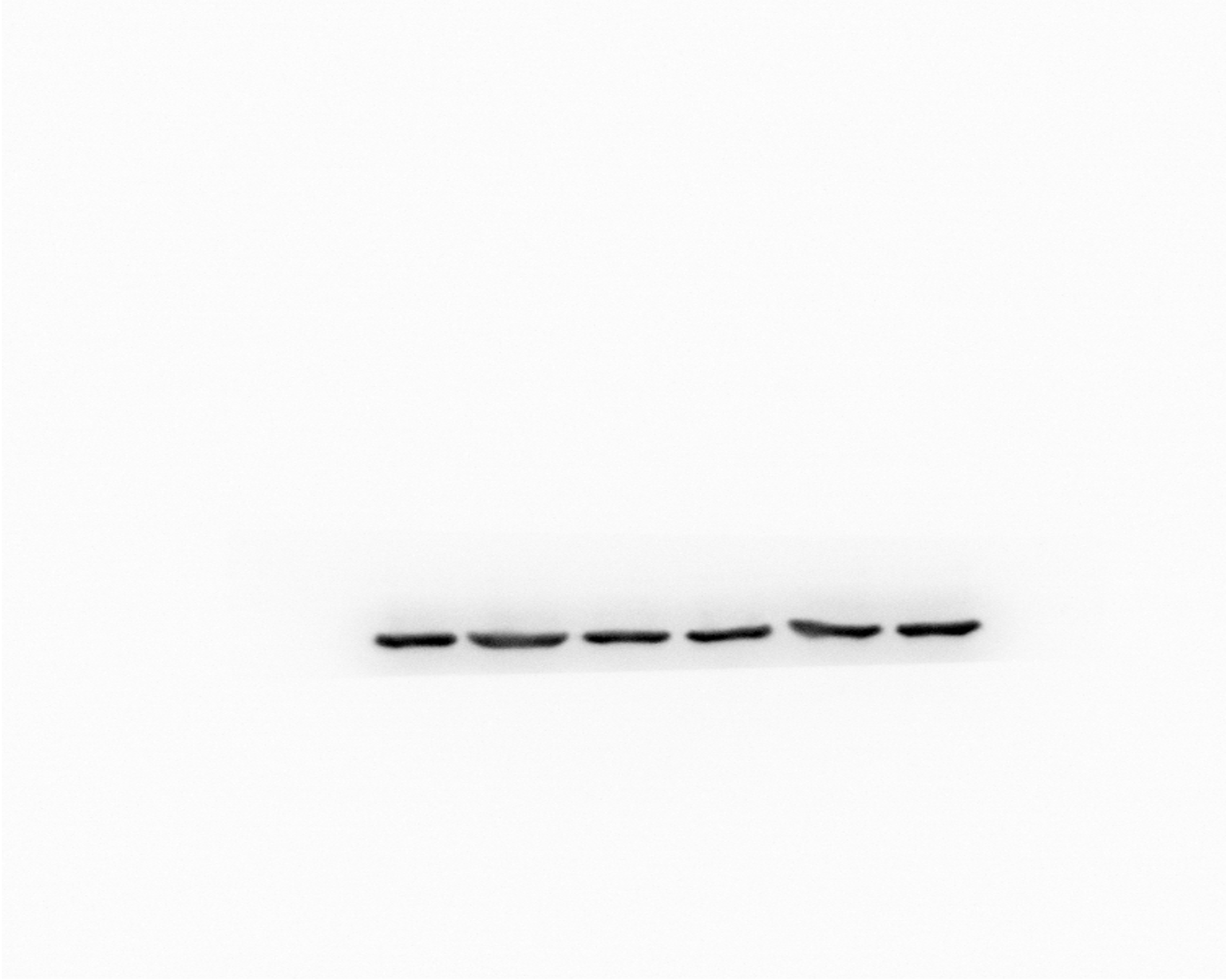

Supplement: Supplementary file 1 — Source Data [file 44321_2026_439_MOESM1_ESM.zip › Source Data/Corrected Figure EV1p actin.tif]
